# Global biosphere productivity response to Atlantic Meridional Overturning Circulation collapse during Heinrich Stadial 4

**DOI:** 10.1126/sciadv.aea0455

**Published:** 2026-07-15

**Authors:** Ji-Woong Yang, Jean-Baptiste Ladant, Thomas Blunier, Amaëlle Landais, Masa Kageyama, Didier Paillard, Nicolas Viovy, Pascale Braconnot, María Fernanda Sánchez Goñi, Frédéric Prié, Sébastien Nguyen, Louise Crinella-Morici

**Affiliations:** ^1^ Laboratoire des Sciences du Climat et de l’Environnement (LSCE, UMR 8212), CEA, CNRS, Université de Versailles Saint-Quentin-en-Yvelines, Université Paris-Saclay, 91191 Gif-sur-Yvette, France.; ^2^ Institut Pierre-Simon Laplace (IPSL), 78280 Guyancourt, France.; ^3^ Physics of Ice, Climate, and Earth, Niels Bohr Institute, University of Copenhagen, 2200 Copenhagen, Denmark.; ^4^ Environnements et Paléoenvironnements Océaniques et Continentaux, Université de Bordeaux, EPHE, Université PSL, CNRS, 33614 Pessac, France.

## Abstract

The slowdown of the Atlantic Meridional Overturning Circulation (AMOC) due to ongoing climate change raises concerns about its impact on the global carbon cycle. Understanding how global biosphere productivity may respond to such changes is essential for predicting the future carbon cycle, yet quantifying past global biosphere productivity remains challenging. We use triple isotope composition of air oxygen (^17^Δ) trapped in polar ice cores to reconstruct the global biosphere productivity over the period 40.5 to 38.6 thousand years before the present (ka B.P.), covering Heinrich Stadial 4, a period of weakened AMOC. Our data reveal a slight increase in productivity following a carbon dioxide (CO_2_) rise around 39.5 ka B.P. Freshwater hosing experiments using a global climate model indicate that the CO_2_ fertilization effect on the terrestrial biosphere offsets productivity decline caused by consequences of AMOC slowdown. Collectively, our results suggest that global biosphere productivity slightly increases in response to AMOC slowdown and accompanying changes.

## Teaser

Global biosphere productivity likely increases in response to abrupt climate change caused by slowdown of ocean circulation.

## INTRODUCTION

Oxygenic photosynthesis in both terrestrial and oceanic biospheres is fundamental to life on Earth as it converts light energy and inorganic carbon into organic matter essential for sustaining ecosystems [e.g., (*1*)]. As the largest flux of carbon between the atmosphere and the biosphere [e.g., (*2*)], photosynthesis has played a pivotal role in shaping Earth’s atmosphere and climate by fixing atmospheric carbon dioxide (CO_2_) and releasing molecular oxygen (O_2_) to the atmosphere [e.g., (*3*, *4*)]. However, global photosynthesis is itself affected by Earth’s climate. Field measurements and satellite observation over recent decades have shown that the global (land and ocean) rate of photosynthesis has varied along with global climate change because of various climatic and environmental factors [e.g., (*2*, *5*–*7*)]. Understanding mechanisms by which global photosynthesis responds to climate change will provide invaluable insight into the future dynamics of the global O_2_ and carbon cycles.

A frequently discussed outcome of ongoing climate change is a slowdown or eventual shut down of the Atlantic Meridional Overturning Circulation (AMOC) [e.g., (*8*, *9*)]. A weakened AMOC can cause substantial climatic and hydrological changes at global scale [e.g., (*10*–*13*)], likely affecting the biological O_2_ cycle through changes in both photosynthesis and respiration in global biosphere. Projections for a collapse of the AMOC are ranging from not before 2100 (*14*) to as early as the mid-21st century (*9*). Numerous studies—using global climate models (GCMs)—have been dedicated to projecting the response of the terrestrial and marine biosphere to the anticipated slowdown of AMOC [e.g., (*15*)]. Nevertheless, the ability of most GCMs to simulate the photosynthesis in global biosphere have not yet been validated under climate boundary conditions different from those of the preindustrial period.

Slowdowns of the AMOC have occurred on several occasions in the past (*16*, *17*), providing an opportunity to investigate the potential impacts of such changes in the future [e.g., (*14*)]. Water-stable isotope ratios measured from Greenland ice cores reveal multiple shifts between cold and warm intervals during the last glacial period, namely Dansgaard-Oeschger (DO) events [e.g., (*18*)], while Antarctic ice core records show gradual warming and cooling, respectively [e.g., (*19*, *20*)]. The asynchronous coupling between temperature evolutions at both poles has been explained by the bipolar-seesaw mechanism, invoking redistribution of heat between the Northern and Southern Hemispheres (NH and SH, respectively) due to changes in AMOC (*21*, *22*). According to this mechanism, during Greenland cold phases (stadials), reduced heat transport from the SH to the NH due to weakened AMOC leads to cooling around the North Atlantic Ocean and warming in the SH. In response to the NH high-latitude cooling, the Intertropical Convergence Zone (ITCZ) migrates south, modifying precipitation patterns in the tropics. Among the stadials, those associated with massive iceberg discharges into North Atlantic Ocean (i.e., Heinrich event) are classified as Heinrich stadials (HSs) (*23*) and characterized by more pronounced climate changes than other stadials. For example, geochemical tracers for past ocean circulation such as ratio of protactinium to thorium isotopes (^231^Pa/^230^Th) indicate a systematic reduction of deep-water formation in North Atlantic during stadials, of which most pronounced changes are observed during HSs [e.g., (*16*)].

The reorganization of the AMOC is also thought to drive substantial changes in the global carbon cycle. Antarctic ice-core measurements show that atmospheric CO_2_ concentrations increase during HSs but do not do so during short non-HSs (*24*, *25*). Although debated, the prevailing interpretation of these millennial-scale CO_2_ rises attributes them to enhanced ventilation of deep waters in the Southern Ocean, likely driven by intensified westerly winds [e.g., (*25*)]. Reduced aeolian dust and iron deposition to the Southern Ocean during stadials may further contribute to the CO_2_ increase by weakening iron fertilization, reducing export production, and increasing oceanic carbon outgassing [e.g., (*26*, *27*)]. More recently, ice-core measurements of methane (CH_4_) isotopic composition suggest widespread biomass burning as an additional source of carbon explaining the abrupt CO_2_ rise (*28*). With a CO_2_ increase of ∼14 parts per million (ppm), which is greater than those observed during the other HSs (*29*), the Heinrich Stadial 4 (HS4) thus provides an ideal case for examining the response of global biosphere to eventual AMOC weakening coupled with rising atmospheric CO_2_.

Scientists have made a huge effort to understand the terrestrial- and ocean-biosphere productivity during HS, relying either on fossil pollen assemblages or on geochemical tracers of export production measured from lacustrine- or marine-sediment cores. Nonetheless, estimating the global biosphere productivity remains challenging because most of previous records are based on indirect and qualitative proxies but also because they record local changes in biological productivity and hence demonstrate a large spatial heterogeneity. For example, a compilation of marine sediment records covering the last glacial period demonstrates a reduction of export productivity in the North Atlantic and North Pacific Oceans and an increase in the Southern Ocean and the Eastern Equatorial Pacific Ocean in response to Heinrich events [e.g., (*30*)]. Earlier modeling studies suggest that the weakening (or shutdown) of the AMOC—generally obtained by adding freshwater to the North Atlantic Ocean—leads to a reduced global terrestrial and marine primary productivity (*30*–*32*). More recent works examining the global carbon-cycle response to AMOC perturbation, while accounting for changes in dust deposition and the land biosphere, indicate reduced marine productivity but increased terrestrial productivity (*26*, *27*).

Triple isotopic composition (δ^17^O and δ^18^O) of air O_2_ offer the possibility to directly access the global biosphere productivity (*33*, *34*). This tracer approach is based on the deviation of ^17^O isotope from the mass-dependent fractionation and hence the triple isotopic composition of air O_2_ and is reported as ^17^Δ ≡ ln(δ^17^O + 1) – λ_ref_·ln(δ^18^O + 1) (*35*, *36*). λ_ref_ is the slope of ln(δ^17^O + 1) versus ln(δ^18^O + 1) by mass-dependent fractionation of O_2_ in the biosphere under steady state and equals to 0.516 (*36*). The O_2_ produced in the biosphere (by photosynthesis) exhibits a positive ^17^Δ value relative to modern air standard, in essence originating from the source water and is not much modified by other fractionation processes occurring in the biosphere [e.g., (*33*)]. On the other hand, the O_2_ circulating through the stratosphere becomes depleted in ^17^O by photochemical reactions that deplete ^17^O and ^18^O isotopes of residual O_2_ in a so-called mass-independent way. The relative contribution of O_2_ fluxes with distinct ^17^Δ signatures determines the ^17^Δ of tropospheric O_2_, which is enclosed in bubbles in polar ice cores. Therefore, the global biosphere O_2_ flux can be estimated by constraining the fractionations in the biosphere and the stratosphere as well as the stratospheric flux.

Note that the global biosphere flux obtained from ^17^Δ approach represents the gross photosynthetic O_2_ productivity (GOP hereafter), that is, the production rate of O_2_ via splitting water molecules in photosystem II, which provides the electrons and energy needed for carbon fixation. GOP differs from gross primary productivity in carbon unit (GPPC hereafter) in dynamic vegetation models in that GPPC refers to the net CO_2_ assimilation rate after subtracting CO_2_ release by photorespiration and day respiration [e.g., (*37*)]. Therefore, GOP provides an upper bound on the potential CO_2_ fixation rate, whereas GPPC is the realized net assimilation rate.

The ice-core measurements of ^17^Δ have so far been applied to reconstruct the GOP variability over glacial-interglacial cycles (*33*, *34*, *38*–*41*). However, the temporal resolution of previous data does not allow the estimation of GOP changes at submillennial timescale. In this study, we attempt for the first time to reconstruct the GOP evolution during HS4 from new high-resolution measurements of ^17^Δ from the North Greenland Eemian Ice Drilling (NEEM) ice cores. In the following, we first describe the calculation of GOP from ice-core ^17^Δ records and then explore potential mechanisms of GOP change during HS4 using specifically designed coupled climate-vegetation-biogeochemistry simulations (see Materials and Methods) performed with the IPSL-CM5A2-VLR GCM (*42*).

## RESULTS

### New ice-core measurements of ^17^Δ from the NEEM ice core and reconstruction of GOP

**Fig. 1.**
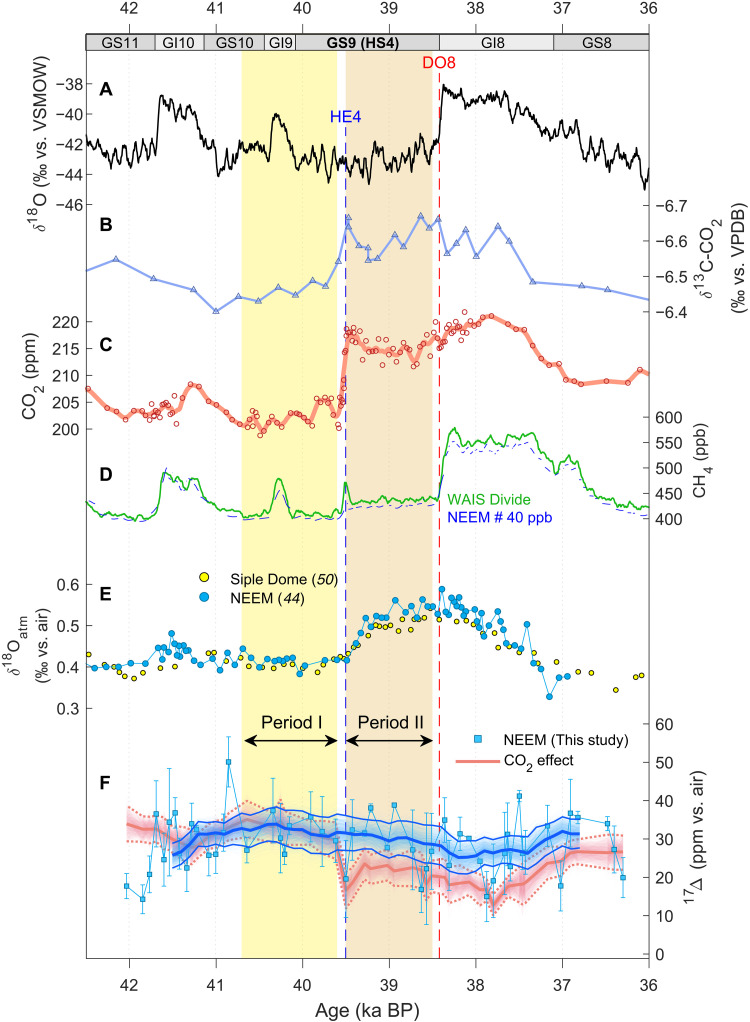
Triple isotope measurements of O_2_ from the NEEM ice core over HS4. (**A**) Water stable isotope records from the North Greenland Ice Core Project (NGRIP) ice core (*100*), smoothed by 50-year moving average. (**B**) Stable carbon isotope ratio of CO_2_ measured from Taylor Glacier (*47*). Following Wendt *et al.* (*29*), the δ^13^C-CO_2_ records are shifted by 160 years from its original chronology. (**C**) Atmospheric CO_2_ concentration measured from the WAIS Divide ice core, Antarctica [in circles; (*29*)] and smoothed curve with 50-year moving average (solid line). (**D**) Atmospheric CH_4_ concentration measured in WAIS Divide [green; (*46*)] and NEEM [blue; (*45*)] ice cores. To facilitate a visual comparison, a constant offset of −40 ppb is applied to the NEEM CH_4_ record. (**E**) δ^18^O of atmospheric O_2_ (δ^18^O_atm_) measured in NEEM [blue; (*48*)] and Siple Dome [yellow; (*49*)] ice cores. (**F**) ^17^Δ records from NEEM ice core (blue). Error bars indicate 1 SEM estimated from duplicate measurements. Also shown in pink are modeled ^17^Δ curve resulting from CO_2_ effect alone (see “New ice-core measurements of ^17^Δ from the NEEM ice core and reconstruction of GOP” section). All data are presented on the WD2014 timescale (see Materials and Methods). Yellow and orange shades indicate the 1000-year intervals before and after the abrupt CO_2_ jump, corresponding to periods I and II, respectively (see “New ice-core measurements of ^17^Δ from the NEEM ice core and reconstruction of GOP” section).

The new ^17^Δ data measured from the NEEM ice core are plotted in Fig. 1. The ^17^Δ data have been corrected for isotopic fractionation due to gravitational settling in the firn column, preferential gas loss during ice-core storage, and bubble close off (see Materials and Methods). Error bars on individual data points represent the SEM from replicate measurements. We smooth the ^17^Δ records by a 1000-year moving average given the turnover time of atmospheric O_2_ being ∼1000 years (*43*). The 95% confidence intervals of the smoothed ^17^Δ curve are estimated from 1000 Monte-Carlo simulations, in which randomly perturbed ^17^Δ time series are generated by adding zero-mean Gaussian noise to each datum (SD equal to the reported 1σ analytical uncertainty) before smoothing. We transferred the gas chronology of the NEEM ice core (*44*) to that of the West Antarctic Ice Sheet (WAIS) Divide (WD2014) by matching CH_4_ concentration records from both drilling sites (*45*, *46*).

The smoothed NEEM ^17^Δ record shows no significant millennial-scale variability from the onset of GS-10 [∼41 years before the present (ka B.P.)] to throughout DO-8 (∼38.5 ka B.P.). In contrast, atmospheric CO_2_ concentration exhibits an abrupt rise of ∼14 ppm at 39.5 ka B.P., remaining elevated through DO-8 (Fig. 1) (*29*). This CO_2_ increase is accompanied by a sharp depletion in the carbon isotopic composition of CO_2_ (δ^13^C-CO_2_), indicating a rapid injection of ^13^C-depleted carbon into the atmosphere (*47*). In addition, the δ^18^O of atmospheric O_2_ (δ^18^O_atm_) becomes progressively enriched following the CO_2_ rise, continuing until DO-8, likely reflecting low-latitude hydrological changes (*32*, *48*–*50*). To interpret our ^17^Δ data under distinct circumstances, we compare two intervals—period I (40.6 to 39.6 ka B.P.; before CO_2_ jump) and period II (39.5 to 38.5 ka B.P.; after CO_2_ jump).

The contribution of stratospheric O_2_ fluxes is attributed to mass-independent fractionation (MIF) of oxygen isotopes in the stratosphere and the flux of stratospheric O_2_ into the troposphere. To first order, both terms scale with atmospheric CO_2_. The stratospheric isotope effect due to oxygen isotope exchange reaction between O_2_ and CO_2_ [via O_3_ and electronically excited atomic oxygen O(^1^D)] depends on the abundance of CO_2_ (*51*, *52*). Furthermore, two-dimensional chemistry-climate model experiments show that rising CO_2_ concentrations accelerate Brewer-Dobson Circulation (BDC) (*53*). Scaling the stratospheric isotope effect and the flux of stratospheric O_2_ accordingly (see Materials and Methods), the ∼14 ppm increase in CO_2_ at 39.5 ka B.P. (between period I and period II in Fig. 1) depletes tropospheric ^17^Δ by 11 ± 5 ppm (1σ). For this calculation (Fig. 1, red line), all other potential influences—biological and hydrological processes—were held constant. This effect (hereafter referred to as the “CO_2_-effect”) must be compensated for, and in the following section, we discuss potential changes that could offset the CO_2_-effect. To assess the influence of each process, we use a box model that captures the major fluxes and associated fractionation processes [(*38*); see Materials and Methods].

**Fig. 2.**
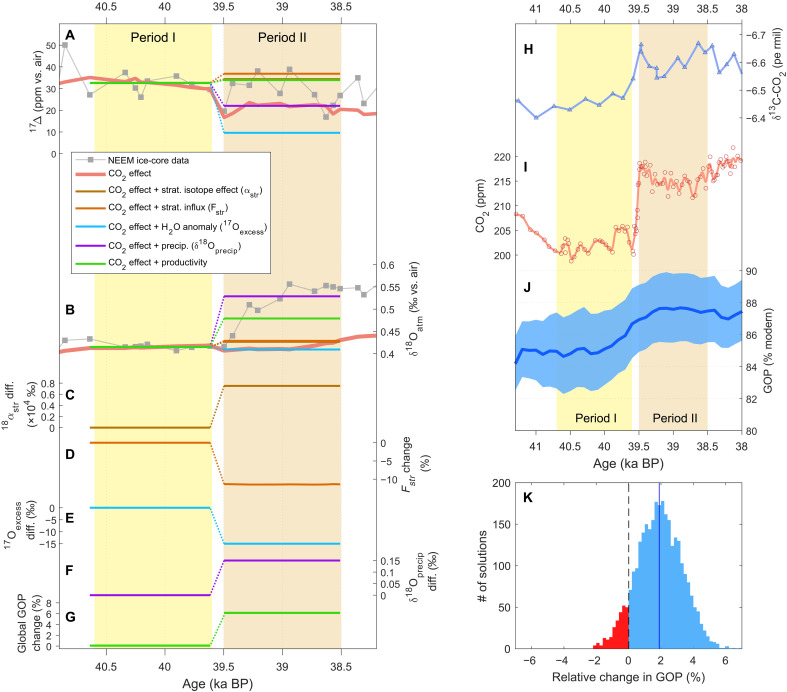
Modeled isotopic composition of O_2_ and global biosphere productivity reconstruction. (**A** and **B**) Modeled ^17^Δ and δ^18^O_atm_ for different scenarios compared with NEEM ^17^Δ and δ^18^O_atm_ records (in gray). (**C** to **G**) Hypothetical time series of forcing factors used to create the modeled ^17^Δ and δ^18^O_atm_: (C) change in the stratospheric isotope effect (^18^α_str_), (D) relative change in the flux of stratospheric O_2_, (E) change in the ^17^O-anomaly of precipitation water consumed by terrestrial plants, (F) change in the δ^18^O of precipitation water (δ^18^O_precip_) consumed by terrestrial plants, and (G) relative change in global biosphere O_2_ productivity (GOP). (**H**) δ^13^C-CO_2_ and (**I**) CO_2_ concentrations between 41 and 38 ka B.P. intervals. (**J**) Reconstructed GOP from NEEM ^17^Δ records, smoothed by 1000-year moving average. Blue envelops indicate 2.5 and 97.5 percentiles of 3000 Monte Carlo simulations. Yellow and orange shades indicate periods I and II, respectively. (**K**) Histogram of relative change of GOP between period II and period I from 3000 Monte Carlo simulations.

In addition to the CO_2_-effect, variability in the stratospheric effect (α_str_) and flux of stratospheric O_2_ into the troposphere (*F*_str_) influence the tropospheric ^17^Δ signal. Balancing the tropospheric ^17^Δ signal by either an increase in α_str_ or a decrease in *F*_str_ is easily achievable (Fig. 2). However, a substantial weakening stratospheric fractionation (i.e., an increase in α_str_) during period II is unlikely for three reasons. First, changing α_str_ to balance ^17^Δ would have a negligible effect on δ^18^O_atm_ as the ^18^ε_str_ = ^18^α_str_ − 1 is expected to be in the order of ∼10^−3^ per mil (‰) (*54*), and thus conflicting with the observed progressive enrichment of δ^18^O_atm_ following the CO_2_ rise, continuing until DO-8. Second, gross O_3_ formation, the primary source of MIF, is expected to change only weakly (see the Supplementary Materials). Our first-order estimate points to a slight acceleration, which would tend to strengthen depletion of stratospheric ^17^Δ during period II. Last, the rate constant of O(^1^D) quenching by CO_2_ is temperature dependent [e.g., (*55*)]. Radiative cooling of the upper stratosphere during period II, associated with rising greenhouse gas concentrations [e.g., (*56*)] would accelerate O(^1^D) quenching by CO_2_ and would likely enhance the ^17^Δ depletion of stratospheric O_2_. Together, these lines of evidence suggest stronger depletion of stratospheric ^17^Δ and therefore imply higher GOP than in our default scenario (see more details in the Supplementary Materials). Also, a reduction of *F*_str_ seems improbable. Independent constraints derived from triple oxygen isotope measurement in ice-core nitrate (NO_3_) suggest that the BDC, the global overturning circulation responsible for the mass transport between the troposphere and the stratosphere [e.g., (*57*)], likely accelerated in response to enhanced latitudinal sea surface temperature (SST) gradients (*58*). Freshwater input during HS4 (period II) likely induced substantial North Atlantic cooling and thus steepened the SST gradient as also indicated by a freshwater hosing experiments using the IPSL-CM5A2-VLR model (fig. S13).

We conclude that stratospheric processes are insufficient to explain the observed ^17^Δ and δ^18^O_atm_ records, which leaves us with processes related to biological productivity. The biogenic ^17^Δ signature is sensitive to hydrological processes affecting the isotopic composition of leaf water, ultimately transferred to that of photosynthetic O_2_. The aforementioned change in δ^18^O_atm_ has been interpreted as a signal of low-latitude hydrological changes linked to a southward shift of the ITCZ (*32*, *48*–*50*), which also affects the ^17^Δ signal in precipitation water (*48*).

Increase in the δ^18^O of precipitation water alone to match the observed δ^18^O_atm_ has a limited impact on ^17^Δ, provided that the ^17^O anomaly of precipitation water (^17^O_excess_) is kept constant, as in the default scenario (Fig. 2). By contrast, any change in ^17^O_excess_ affects our GOP calculation as it alters the ^17^Δ signature of photosynthetic O_2_. The relevant quantity is the GOP-weighted average of ^17^O_excess_. In a sensitivity test, a 15-ppm decrease in ^17^O_excess_ produces an additional ∼20-ppm depletion in ^17^Δ (Fig. 2). This model sensitivity implies that an enrichment of ^17^O_excess_ by ∼8 ppm in period II (relative to period I) could offset the CO_2_-effect without requiring a change in GOP. However, our analysis of modern seasonal variability in precipitation ^17^O_excess_ indicates that plausible changes in GPP-weighted ^17^O_excess_ are lower than 0.4 ppm (see details in the Supplementary Materials). We therefore consider an ∼8-ppm enrichment in period II unlikely, so we favor the constant ^17^O_excess_ default scenario over the study interval.

Together, we deem it likely that an increase in GOP counterbalances the CO_2_-effect, increasing the release of enriched ^17^Δ to the atmosphere. A scenario with enhanced GOP effectively counterbalances the CO_2_-effect but cannot fully account for the enrichment of δ^18^O_atm_ (Fig. 2). The slight enrichment in δ^18^O_atm_ under the enhanced GOP scenario results from changes in global respiration under steady-state conditions (see Materials and Methods). These results suggest that the ^17^Δ variations across HS4 can be mostly explained by increase in GOP, while the δ^18^O_atm_ variations are primarily driven by changes in the hydrological cycle. We therefore consider a combined scenario involving changes in GOP and the hydrological cycle to be the most plausible explanation.

On the basis of the above principle, we estimate the GOP variations from ice-core ^17^Δ and δ^18^O_atm_ records by solving inversely our box model (see Materials and Methods). The reconstructed GOP from our “best-guess” scenario is plotted in Fig. 2. Monte Carlo simulations (*n* = 3000) incorporating random perturbations with analytical uncertainty of ice-core ^17^Δ show that the mean GOP during period II is about 2% higher than in period I (Fig. 2K). Less than 9% of 3000 solutions indicate a decrease in GOP in period II.

Our finding contrasts with previous modeling-based studies that suggest a decline in terrestrial biosphere GOP (*32*) and marine primary productivity (*30*) in response to AMOC slowdown. We attribute this discrepancy to the fact that those studies primarily focused on the impacts of freshwater hosing into the North Atlantic Ocean and did not account for additional factors, such as rising atmospheric CO_2_ concentrations and changes in dust deposition, that may modulate or counterbalance the effects of AMOC weakening.

### GCM experiments

To gain insight on the mechanisms that could drive GOP changes across HS4, we carried out five experiments using IPSL-CM5A2-VLR GCM with different set of forcing factors (see Materials and Methods for additional details). On a control experiment with boundary conditions of 40 ka B.P. (Ctrl_40ka), we introduce perturbations that are added incrementally on the preceding simulations (Table 1): freshwater hosing in the North Atlantic (Exp_FWF), dust forcing characteristic of an HS (Exp_DUST), and CO_2_ change associated with HS4 (Exp_CO2). The final simulation (Exp_VFIX) is identical to Exp_CO2 but the vegetation cover is fixed to that obtained at the end of Ctrl_40ka. All but Exp_VFIX IPSL-CM5A2-VLR coupled model simulations are run in dynamic vegetation mode, thereby allowing plant functional types (PFTs) to evolve interactively in response to the simulated climate. We prescribe the simulated Last Glacial Maximum (LGM, 21 ka B.P.) dust deposition field (*59*) for Ctrl_40 ka and Exp_FWF experiments and H1 (16 ka B.P.) dust deposition field (*60*) for Exp_DUST, Exp_CO2, and Exp_VFIX because our GCM simulation does not include an interactive aerosol module.

**Table 1.** List and description of the experiments conducted in this study.

Experiment	Hosing	Dust deposition	CO_2_	Vegetation
Ctrl_40ka	No	LGM (21 ka B.P.)	200 ppm	Dynamic
Exp_FWF	Yes	LGM (21 ka B.P.)	200 ppm	Dynamic
Exp_DUST	Yes	H1 (16 ka B.P.)	200 ppm	Dynamic
Exp_CO2	Yes	H1 (16 ka B.P.)	210 ppm	Dynamic
Exp_VFIX	Yes	H1 (16 ka B.P.)	210 ppm	Fixed to Ctrl_40ka

**Fig. 3.**
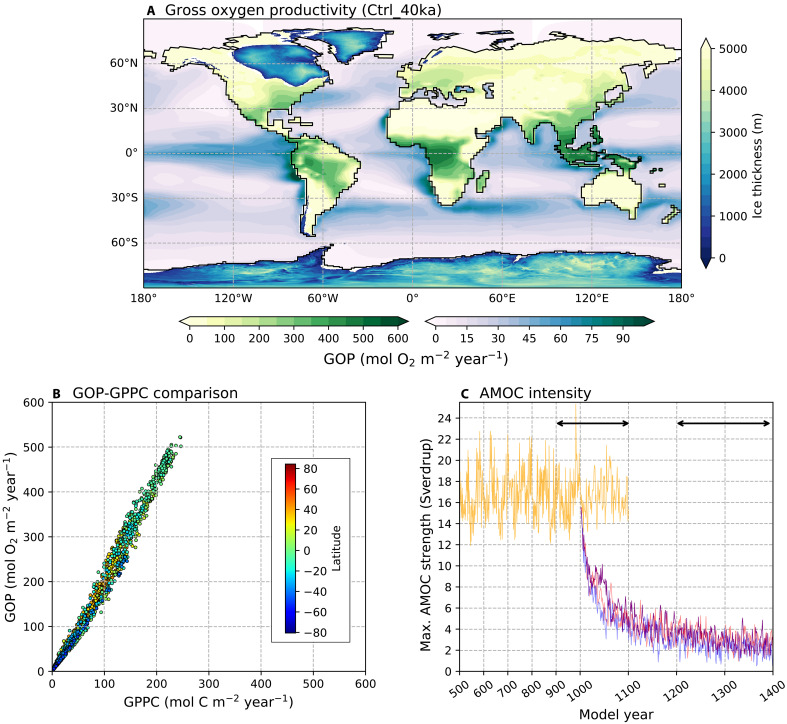
GOP and AMOC for 40-ka control simulation. (**A**) Global distribution of terrestrial and marine GOP obtained in the Ctrl_40ka experiment. (**B**) Scatter plot for GOP versus GPPC (moles of carbon per square meter per year) obtained in ORCHIDEE land surface model. GOP represents the gross O_2_ production rate by water splitting at photosystem II, while GPPC is computed as net CO_2_ assimilation rate that accounts for CO_2_ release by photorespiration and other daytime respiration [e.g., (*80*)]. (**C**) Time series of annual maximum strength of AMOC (in sverdrup) for Ctrl_40ka experiment (yellow) and perturbation experiments (purple: Exp_CO2, red: Exp_DUST, blue: Exp_VFIX). The black arrows indicate the period of averaging for comparisons.

The Ctrl_40ka experiment results in a global terrestrial and marine GOP of 20.8 and 8.8 pmol of O_2_ year^−1^, respectively, yielding a global GOP of 29.6 pmol of O_2_ year^−1^ (Fig. 3). The 40-ka GOP obtained in our control experiment is ∼84.5% of the value from the preindustrial experiment performed with identical version of GCM (IPSL-CM5A2-VLR) (*42*). This result is in good agreement with the value obtained from ice-core ^17^Δ records.

### Effect of freshwater hosing on global biosphere productivity

**Fig. 4.**
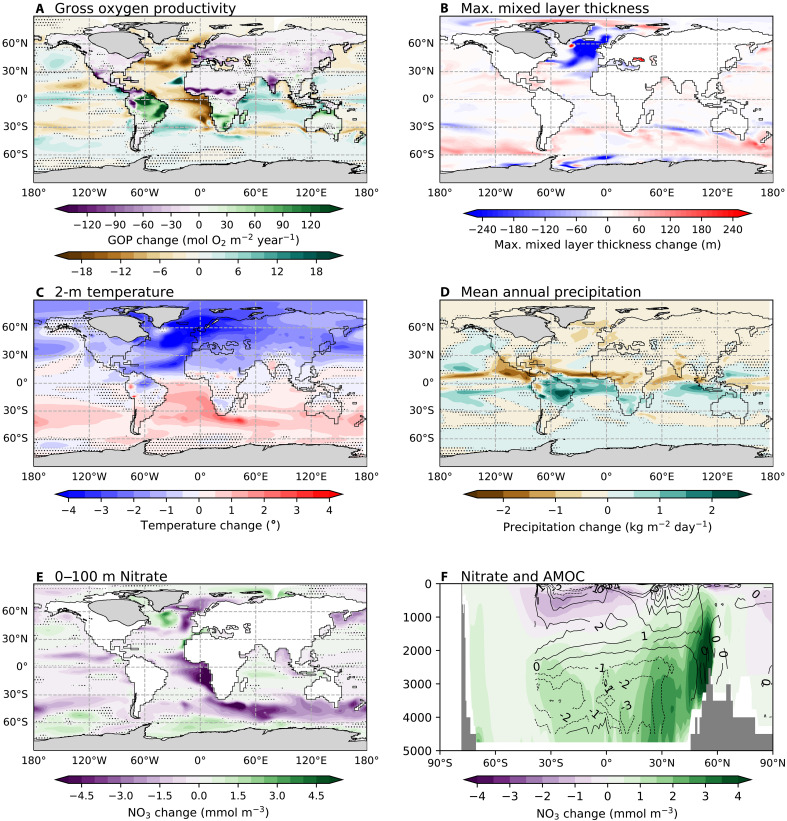
Effects of freshwater hosing in the North Atlantic (Exp_FWF minus Ctrl_40ka). Figures show changes in (**A**) global terrestrial and marine gross oxygen productivity, (**B**) maximum mixed layer thickness, (**C**) 2-m temperature, (**D**) mean annual precipitation, (**E**) surface ocean nitrate concentration, and (**F**) vertical distribution of nitrate concentration in the Atlantic basin. Black contours on (F) indicate zonal mean stream function (in sverdrup) of AMOC in our hosing-only experiment (Exp_FWF). The results are obtained by averaging the last 200 years of integration for both experiments. Where available, stippled regions are not significant at 95% confidence level, according to two-sided Student’s *t* test.

Our hosing-only experiment (Exp_FWF) results in a slowdown of the AMOC from ∼17 sverdrup in the control experiment (Ctrl_40ka) to ∼2 sverdrup. The maximum intensity of AMOC becomes lower than ∼4 sverdrup after 200 years of hosing (Fig. 3). The reorganization of AMOC leads to a decreased GOP in both the terrestrial and marine biosphere compared to the control experiment (Fig. 4A). Globally, the Exp_FWF experiment yields a GOP of 28.4 ± 0.4 pmol of O_2_ year^−1^ or 96 ± 2% of the GOP in Ctrl_40ka simulation (table S2).

More specifically, the reduction in land GOP is associated with changes in near-surface temperature and precipitation. Consistent with paleoclimate reconstructions [e.g., (*23*)], Exp_FWF experiment exhibits a strong cooling over the North Atlantic and Western Europe and an overall warming in the SH. Mean annual precipitation decreases in the northern tropics and increases in the southern tropics, indicating a southward shift of ITCZ in response to abrupt cooling in the NH [e.g., (*61*)] (Fig. 4, C and D). The land GOP is principally limited by temperature and precipitation. As shown by Chen *et al.* (*62*), precipitation (and hence water availability) is the dominant limiting factor for low-latitude GOP, whereas in mid- to high-latitude regions, GOP is more likely limited by temperature. As a result, land GOP declines widely across the NH, particularly in Europe, northernmost South America, northern tropical Africa, and India. This decline is not fully offset by increases in land GOP found in limited regions of the Amazon, South Africa, and northern Australia (Fig. 4A).

Marine productivity is primarily limited by nutrient availability. The freshwater hosing suppresses convection and significantly reduces mixed-layer thickness in the North Atlantic Ocean (Fig. 4B). A thinner mixed layer limits the upward transport of nutrients from nutrient-rich deep waters (*30*, *63*). This is directly responsible for the reduction of marine productivity in North Atlantic Ocean. In the Eastern Equatorial Atlantic (Bay of Guinea), the strong decrease in surface ocean nutrient availability and the associated reduction in marine productivity are likely driven by reduced nutrient supply from Antarctic Intermediate Water and Sub-Antarctic Mode Water (Fig. 4, E and F).

### Effect of dust deposition change on marine productivity

**Fig. 5.**
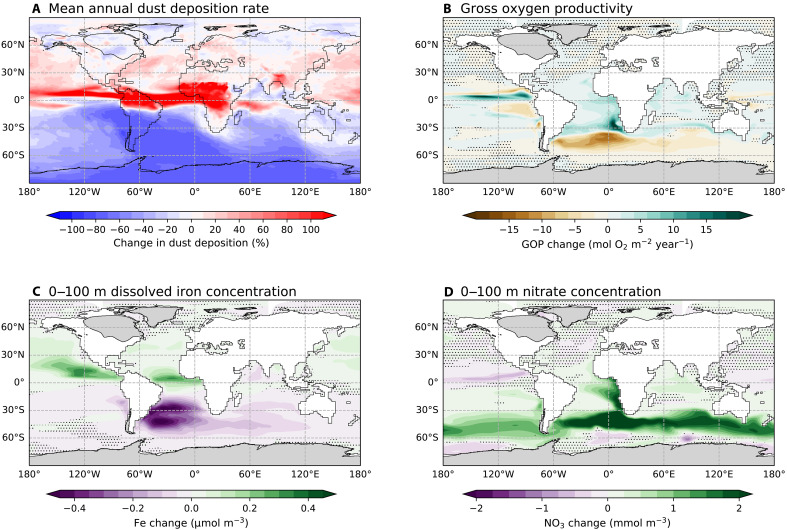
The effects of aeolian dust deposition change on marine productivity (Exp_DUST minus Exp_FWF). (**A**) Mean-annual dust deposition change between 16 ka B.P. (H1) and 21 ka B.P. (LGM) scenarios (*59*, *60*). (**B**) Changes in gross oxygen productivity. (**C**) Changes in surface (1- to 100-m depth) dissolved iron concentration and (**D**) surface nitrate concentration. Terrestrial GOP change is not shown because dust deposition field is only used in PISCES (see “Effect of dust deposition change on marine productivity” section). Where available, stippled regions are not significant at 95% confidence level, according to two-sided Student’s *t* test.

Our dust-forcing experiment (Exp_DUST) has a minor effect on the global GOP. This is because, in our simulation setup, dust deposition affects marine GOP only, and local changes in marine GOP largely cancel out (Fig. 5B). As a consequence, Exp_DUST experiment leads to an increase in global marine GOP by only 0.1 pmol of O_2_ year^−1^ (∼1.2% relative to the Exp_FWF simulation), which is insufficient to offset the global GOP decline caused by freshwater hosing (table S2).

The H1 dust deposition rate exhibits a strong increase along the Equatorial Pacific and Atlantic, by more than a factor of 2 in some regions, with an overall increase in the NH but a significant reduction in the SH compared to the LGM field (Fig. 5A). Accordingly, the simulated marine GOP shows an increase along the Eastern Equatorial Pacific (Fig. 5B), where the marine productivity is limited by iron. The response of marine GOP is less pronounced in the Equatorial Atlantic (Bay of Guinea) as the marine productivity in this region is less limited by iron. Meanwhile, south of ∼40°S, Exp_DUST experiment shows a significant reduction in marine GOP in the Atlantic sector of the Southern Ocean because productivity is primarily limited by iron in the Southern Ocean and the reduced aeolian dust supply lowers iron input (Fig. 5A). The lower productivity (i.e., lower nutrient consumption) leaves the Southern Ocean water masses enriched in other nutrients (e.g., nitrate) (Fig. 5D). The subsequent upwelling of these water masses off eastern South Africa brings nutrient-enriched waters to the surface and enhance productivity (Fig. 5B), which explains the strong simulated increase in GOP in the western coast of South Africa (Southern Benguela coast) (Fig. 5B).

### Effects of CO_2_ forcing

**Fig. 6.**
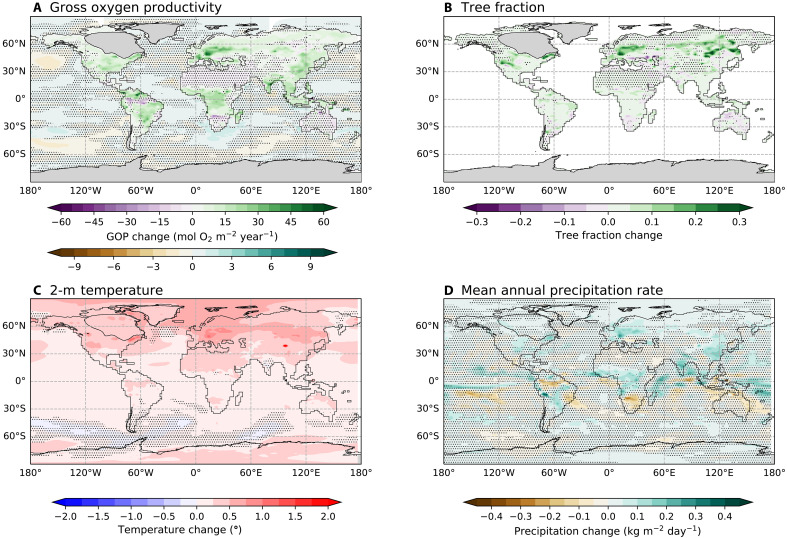
The effects of CO_2_ on global productivity (Exp_CO2 minus Exp_DUST). (**A**) Changes in global terrestrial and marine biosphere gross oxygen productivity. (**B**) Changes in tree fraction, (**C**) changes in 2-m temperature, and (**D**) mean annual precipitation rate. Where available, stippled regions are not significant at 95% confidence level, according to two-sided Student’s *t* test.

Our CO_2_-forcing experiment (Exp_CO2) results in an enhancement of the global terrestrial GOP by ∼1.1 pmol of O_2_ year^−1^ (∼5.5%) relative to that from Exp_DUST due to the CO_2_ fertilization effect and associated climate feedbacks (Fig. 6A). In contrast, the CO_2_ forcing has negligible influence on marine GOP (Fig. 6A) as the phytoplankton growth is not limited by CO_2_ (or by inorganic carbon) in the version of PISCES used here (*64*). Overall, the CO_2_-driven increase in terrestrial GOP in Exp_CO2 raises the simulated global GOP to 29.7 pmol of O_2_ year^−1^, bringing it close to the value in the control experiment (table S2).

The increase in land GOP by the prescribed 10-ppm rise in CO_2_ concentration mostly occurs in the tropics (between 30°S and 30°N) and the NH mid to high latitudes (between 30°N and 90°N), contributing ∼0.5 and 0.4 pmol of O_2_ year^−1^, respectively. The respective changes in GOP for tropical (PFTs 2 and 3), temperate (PFTs 4, 5, and 6), and boreal (PFTs 7, 8, and 9) trees are +0.68, +0.20, and +0.26 pmol of O_2_ year^−1^, corresponding to relative changes of +8, +6, and +16% compared to Exp_DUST experiment. Our results indicate a stronger sensitivity of boreal trees to CO_2_ change compared to tropical and temperate PFTs. This appears inconsistent with the results of Chen *et al.* (*62*), where the ORCHIDEE-MICT dynamic global vegetation model (DGVM) is run off-line with prescribed climate and CO_2_. The authors found that tropical trees are more sensitive to CO_2_ fertilization effect than temperate or boreal type PFTs (*62*). Their results are obtained by single forcing experiments by CO_2_, while ours represent comprehensive results including feedbacks by climate and vegetation changes. The pronounced response of boreal forests is also seen in tree fraction change that shows an increase in NH high-latitude vegetation in response to 10-ppm rise of CO_2_ (Fig. 6B). If the low sensitivity of boreal trees to CO_2_ change is held in our model, the seemingly stronger sensitivity to CO_2_ in our experiments can be explained by climate effect, which is amplified by climate-vegetation feedback. Exp_CO2 simulation leads to an increase in mean annual temperature of ∼0.5° to 1°C over most of NH continental high latitudes compared to Exp_DUST (Fig. 6C) as well as an increase in mean annual precipitation in Europe, East Asia, and Africa (Fig. 6D). Therefore, an initial increase in GOP stimulates the growth of boreal forest biomass and hence reduces surface albedo, which in turn increases temperature and further enhances GPP. Conversely, the limited change in mean annual surface temperature and precipitation (Fig. 6, C and D), and tree fraction (Fig. 6B), in the tropics suggest that the GOP in this region is essentially driven by the change in the CO_2_ fertilization effect (Fig. 6A).

The response of marine GOP to 10-ppm increase in CO_2_ in our model is negligible, with 8.6 ± 0.1 pmol of O_2_ year^−1^ in Exp_DUST and 8.7 ± 0.1 pmol of O_2_ year^−1^ in Exp_CO2 (Fig. 6A and table S2). Temperature changes may alter marine primary productivity but the increase in surface ocean temperatures is small, except in the Arctic and in the highest latitudes of the North Atlantic Ocean (Fig. 6C), which are less productive regions because of limited light and nutrient availability (Fig. 3A).

### Effect of dynamic vegetation

**Fig. 7.**
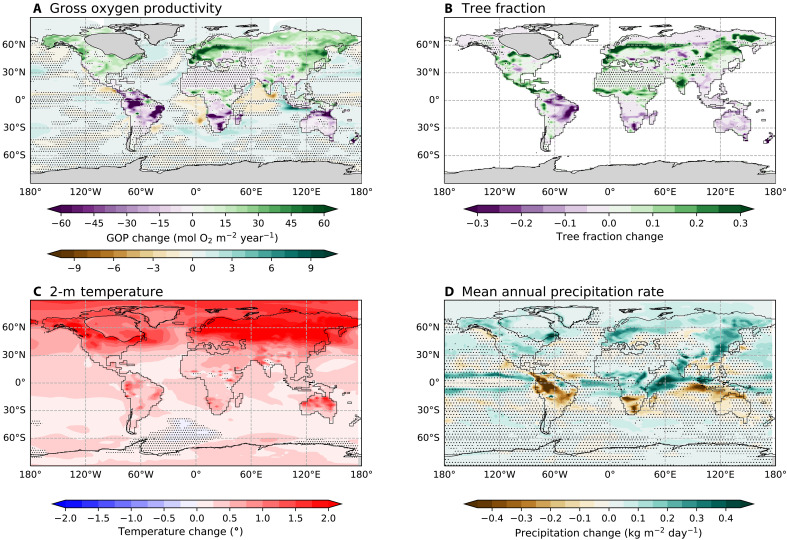
Effects of vegetation change (Exp_VFIX minus Exp_CO2). (**A**) Changes in global terrestrial and marine biosphere gross oxygen productivity. (**B**) Changes in tree fraction, (**C**) changes in 2-m temperature, and (**D**) in mean annual precipitation rate. Where available, stippled regions are not significant at 95% confidence level, according to two-sided Student’s *t* test.

**Fig. 8.**
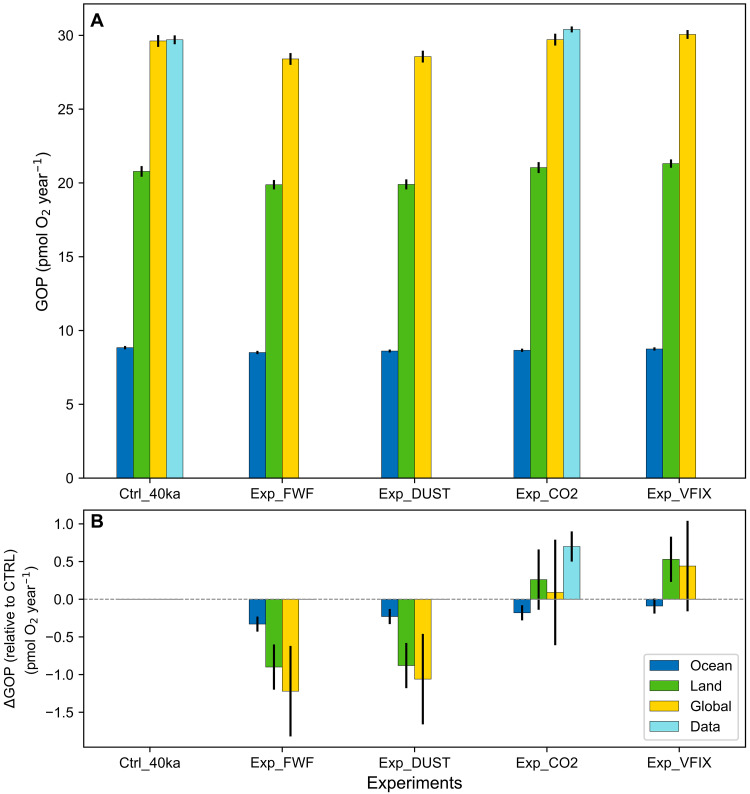
Comparison of GOP obtained in this study. (**A**) The marine (blue), terrestrial (green), and global (yellow) GOP obtained in IPSL-CM5A2-VLR experiments, compared with results from ice-core ^17^Δ data (cyan) for periods I (corresponds to Ctrl_40ka) and II (Exp_CO2). The black error bars represent the 1-sigma variation of model outputs over the integrated period. The GOP values from ice-core data are calculated by multiplying the preindustrial GOP obtained from IPSL-CM5A2-VLR experiments. (**B**) Identical to top panel but shows differences relative to Ctrl_40ka experiments.

Changes in vegetation cover introduce additional feedback mechanisms that can ultimately influence global biosphere productivity. Our fixed vegetation experiment (Exp_VFIX) results in a 0.3 pmol of O_2_ year^−1^ (∼1.3%) terrestrial GOP increase compared to Exp_CO2 (Figs. 7A and 8 and table S2). Fixed vegetation simulation demonstrates a slight increase in marine GOP by ∼1% compared to that of Exp_CO2; however, the increment in marine GOP (∼0.09 pmol of O_2_ year^−1^) is of similar order of magnitude as the interannual variability of marine GOP of ∼0.11 pmol of O_2_ year^−1^ (SD of marine GOP over averaged period), and, hence, we consider that the effects of vegetation evolution on marine productivity is limited (Fig. 8 and table S2).

The global distribution of GOP changes shows an overall decrease in GOP in SH and increase in NH (Fig. 7A). The terrestrial GOP enhancement in NH (0.86 pmol of O_2_ year^−1^) outweighs the reduction in the SH (0.62 pmol of O_2_ year^−1^). These spatial GOP changes strongly correlate with shifts in tree fraction, highlighting a substantial advance of boreal forests in NH and a retreat of tropical forests at lower latitudes (Fig. 7B). The advance of boreal forests at high NH latitudes is associated with additional warming and increased precipitation (Fig. 7, C and D).

As shown in Fig. 7, change in vegetation cover has substantial impact on terrestrial GOP (Fig. 7A), highlighting the importance of the ability of the DGVM to correctly simulate these vegetation changes in response to climate forcing. Comparison with available palynological data shows that our model experiments reasonably capture similar changes in vegetation cover (see the Supplementary Materials). For instance, in Europe, pollen records demonstrate a retreat of boreal and temperate forests during Greenland Stadial (GS) 8/9 (HS4) compared to Greenland Interstadial (GI) 8 (*65*). Compared with the vegetation cover prescribed from Ctrl_40ka in Exp_VFIX, the Exp_CO2 simulation run in DGVM mode captures the retreat of boreal and temperate forests in Europe, which is consistent with palynological records, but it does not reproduce the dominance of herbaceous taxa indicated by the pollen assemblages. The Exp_CO2 experiment also leads to an expansion of bare soil fraction (fig. S19), although such an increase in the proportion of bare soil cannot be recorded by pollen assemblage data. In addition, this discrepancy in the extent of vegetation change between the pollen data and our model may stem in part from differences in the magnitude of climate forcing between the periods compared—namely, between 40 ka and HS4, and between GI8 and GS8/9.

The comparison of Exp_VFIX and Exp_CO2 hints at the role of vegetation during AMOC reorganization. The initial cooling triggered by AMOC slowdown reduces both GOP and boreal forest extent, leading to increased surface albedo and decreased evapotranspiration—both of which further suppress GOP. Similarly, the expansion of tropical and temperate forests in the Amazon and South Africa corresponds to regions of higher mean annual precipitation, potentially enhancing vegetation GOP.

## DISCUSSION

Here, we present a new ^17^Δ record, a unique tracer of past global GOP, from the NEEM ice core covering the HS4 interval. The new ^17^Δ records and box model experiments point to an increase in GOP following the abrupt CO_2_ jump at ∼39.5 ka B.P. (in WD2014 age scale). Our GCM experiments of the idealized Heinrich-like forcing (hosing + dust + CO_2_ + dynamic vegetation) demonstrate a nonsignificant change in GOP, contrary to previous studies [e.g., (*32*)].

Past modeling studies have suggested a decline in GOP in their idealized Heinrich-like GCM experiments as the AMOC collapse leads to NH cooling and drying, along with ocean circulation changes that reduce terrestrial (*32*) and marine (*30*) productivity. We observe similar results in our freshwater hosing-only experiment (Exp_FWF), suggesting that the physical consequences (i.e., cooling and drying of the NH and warming and moistening of the SH) of a collapse of the AMOC on global biosphere production is robust. However, the inclusion in the model of additional climatic and environmental changes documented in the proxy record across HS, in particular, the atmospheric CO_2_ concentration and aeolian dust inputs, allows it to counterbalance the GOP decline resulting from the collapse of the AMOC (Exp_CO2 experiment). In particular, the fertilization effect and the modest warming associated with the imposed CO_2_ increase stimulate terrestrial productivity, thereby allowing global biosphere productivity to rebound to a level similar (within uncertainties) to that of the Ctrl_40ka experiment. Although our new ^17^∆ measurements indicate an increase in GOP, the findings from our GCM simulations are informative because they provide potential feedback mechanisms for compensating the effects of AMOC weakening on global biosphere productivity.

Several factors may help explain the remaining mismatch between the ice-core ^17^Δ record and our numerical experiments using IPSL-CM5A2-VLR GCM. First, we prescribed a constant freshwater flux of 0.2 sverdrup, which results in an almost complete collapse of the AMOC. Recent work instead suggests that the AMOC may have survived at a state of intermediate intensity during HS (*66*). This may reduce the magnitude of GOP decrease driven by the HS4 climate perturbation and, combined with the effects of CO_2_ and dust, enhance global productivity in our HS4-like experiments. Second, unlike the marine productivity, the terrestrial productivity does not rely on nutrient supply in our vegetation model. This means that any changes in aeolian nutrient supply over continental areas do not affect the simulated terrestrial GOP. Yet, Heinrich-like simulations exhibit large changes in dust supply [e.g., (*60*)] (Fig. 5), including more than a doubling over tropical Africa and Amazonia, which correspond to highly productive regions. This could have stimulated terrestrial productivity during HS4 and could be investigated with DGVM including aeolian nutrient deposition [e.g., (*67*, *68*)]. Third, our Exp_CO2 simulation underlines the impact of a prescribed CO_2_ rise of 10 ppmv on global biosphere productivity, whereas WAIS Divide records indicate a 13-ppmv rise between the averages of period I and period II (*29*), which could further enhance terrestrial productivity. Fourth, marine biogeochemical feedbacks may also affect the global productivity. In our simulations, the riverine inputs in nutrients are fixed in time and phytoplankton growth is not limited by dissolved inorganic carbon in PISCES despite laboratory evidence for possible fertilization effects of inorganic carbon or CO_2_ (*69*, *70*). In addition, changes in seawater iron solubility during glacial periods [e.g., (*71*)] may have modulated the global GOP response to variations in dust inputs and AMOC slowdown [e.g., (*72*)], but the magnitude and sign of this effect remain poorly constrained. Addressing the abovementioned limitations may allow better reconciliation of the GOP reconstruction from the ice-core ^17^Δ records and GCM experiments.

The interpretation of ice-core ^17^Δ records could also be improved using isotope-enabled Earth system and/or a coupled climate-chemistry model. Such models would better represent spatial variability in source-water isotopic composition, vegetation-related fractionation, the isotopic composition of dissolved O_2_ in the ocean, and the sensitivity of the stratospheric isotopic effect to CO_2_ and other potential perturbations of the stratosphere [e.g., (*73*)]. These advances however would require substantial future model development work.

Our interpretation of ice-core ^17^Δ records is consistent with the hypothesis of rapid vegetation regrowth following the extended biomass burning (*28*). This hypothesis is proposed by experiments with a carbon-cycle box model (*25*) incorporating the abrupt terrestrial carbon release by biomass burning, which require an enhanced net primary productivity to stabilize CO_2_ and δ^13^C-CO_2_ following the abrupt rise (*28*). Our results thus provide an independent evidence for the increase in global terrestrial biosphere productivity during this interval. The extended biomass burning may result in a sharp decline in GOP at ∼39.5 ka B.P. followed by a rapid increase over a few hundreds of years. However, temporal resolution of current dataset does not allow proper resolution of these even finer-scale temporal variations. Higher-precision, subcentennial-scale measurements of ^17^Δ will be necessary to capture submillennial changes during HS4, as indicated by various paleoclimatic proxy records from this interval [e.g., (*48*, *74*)].

Overall, from our new ice-core measurements of ^17^Δ from NEEM ice core and climate model experiments, we conclude that GOP more likely increases during HS4, following the abrupt CO_2_ jump at 39.5 ka B.P. Although our GCM simulations do not show a clear increase in global GOP, we demonstrate that the abrupt CO_2_ emission and its fertilization effect offsets the decline in biosphere productivity caused by abrupt climate change associated with AMOC slowdown.

If future AMOC perturbation occurs alongside abrupt CO_2_ emission—either via deep ocean ventilation or enhanced biomass burning—the global biosphere will likely contribute to dampen the CO_2_ rise. To better understand this potential feedback, a dedicated study combining δ^13^C-CO_2_ and ^17^Δ would be valuable in shedding light on interactions between biosphere productivity and change in carbon reservoirs.

## MATERIALS AND METHODS

### Triple oxygen isotope analysis of air O_2_ from ice-core samples

We measured the δ^17^O and δ^18^O of air O_2_ enclosed in the NEEM ice-core samples over 36.7– to 42.1–ka B.P. interval. The NEEM ice-core samples used in this study were granted upon approval of the Steering Committee. The ice-core measurements were made at Laboratoire des Sciences du Climat et de l’Environnement (LSCE, Saclay, France) using the semiautomated gas extraction and purification system similar to that described in Barkan and Luz (*75*). In brief, ∼20 g of ice samples is melted in preevacuated glass flasks submerged in a tap water bath at ambient temperature (20°C) for 40 min. The extracted air is passed through H_2_O (liquid nitrogen) and CO_2_ trap (liquid N_2_ and ethanol mixture) and is then gas chromatographed at 0°C to separate O_2_ and Ar from N_2_. The O_2_-Ar mixture is cryogenically trapped by stainless steel tubes submerged in liquid helium reservoir. The samples tubes rest in ambient temperature for ∼3 hours for untrapping and homogenization before injection to Thermo Fisher Scientific Finnigan MAT 253 isotope-ratio mass spectrometer.

The ^17^Δ data have been corrected for gravitational enrichment, pore close-off, and gas loss during sample storage. The gravitational settling effect in the firn column is corrected using published NEEM δ^15^N-N_2_ data (*48*). The correction for pore close-off is in the order of 13 ppm based on the ^17^Δ measurements from the firn-ice transition zone at the NEEM drilling site (*40*). The gas-loss effect has been quantified by parallel analysis of O_2_-Ar ratio (*40*, *41*).

The standard of choice is contemporary air collected in Saclay, France, whose triple isotopic composition of O_2_ and molecular ratio of O_2_ to Ar are determined by regular measurements. The contemporary air is collected in early morning at each day of ice-core measurements. At least two contemporary air samples are measured to trace daily blank effect and bias among the different sample tubes. The first sample is used for priming the gas purification line and whose result is not used in our analysis. Daily measurements of modern air allow us to trace daily variations of blank effect of the extraction system.

### Synchronization of the ice-core records to the WD2014 timescale

We synchronize the ice-core records used in the analysis to the WAIS Divide age scale (WD2014). The NEEM δ^18^O_atm_ and ^17^Δ data are transferred to WD2014 timescale by matching the continuous CH_4_ records from both drilling sites (*45*, *46*). The Siple Dome δ^18^O_atm_ record is synchronized to the NEEM CH_4_ record on WD2014 timescale using the tie points determined by Guillevic *et al.* (*48*). The North Greenland Ice Core Project water isotope record is transferred to WD2014 scale by multiplying a constant factor of 1.0063 to its original age following Buizert *et al.* (*76*).

### The ^17^Δ box model

We use the box model of triple isotope composition of O_2_ developed by Blunier *et al.* (*38*), which accounts for major fluxes and fractionation effects of O_2_ in the biosphere, hydrosphere, and stratosphere. The time derivative of the mass of ^33^O_2_ (^17^O^16^O) and ^34^O_2_ (^18^O^16^O) are given as follows

ddt(MRatmx)=FopαopxRswx−ForαorxRatmx+FlpαlpxRswx−FlrαlrxRatmx+FstrαstrxRatmx−FstrRatmx (1)

where superscript x indicates rare oxygen isotopes of 17 and 18, M the mass of troposphere, Rx the isotopic ratio, F the oxygen flux, and α the isotopic fractionation factor. To calculate total masses of rare isotopologs, we assume that the masses of rare isotopologs are negligible so that MO18O16=MO18O2. The subscript of o and l represents ocean and land processes; p and r the photosynthesis and respiration; and atm, sw, and str the troposphere, seawater, and the stratosphere, respectively. We assume that *M* is constant, and isotopic ratios of air O_2_ are in steady state [i.e., ddt(MRatmx)≈0]. The latter is justified by relatively long atmospheric lifetime of O_2_ [e.g., (*43*)].

The above system is underconstrained, with six unknowns (*F*_op_, *F*_lp_, *F*_or_, *F*_lr_, *h*, and α_str_) but only two equations for ^33^O_2_ and ^34^O_2_. To reduce the number of unknowns, we assume that the terrestrial and ocean biospheres are at equilibrium (i.e., *F*_op_ = *F*_or_ and *F*_lp_ = *F*_lr_). We determine the preindustrial *h* and α_str_ values during the model initialization using preindustrial boundary conditions (i.e., δ^18^O_atm_ = 0‰, ^17^Δ = 0 ppm, *F*_lp_ = 2.43 pmol year^−1^ and *F*_op_ = 1.08 pmol year^−1^). We obtain *h* = 0.6274 and ^18^ε_str_ = ^18^α_str_ − 1 = −1.013 × 10^−6^. To calculate the past variations of ^18^α_str_, we assume that ^18^ε_str_ varies proportionally with atmospheric CO_2_, based on the fact that CO_2_ plays important role in depleting heavy oxygen isotopes in O_2_ (*51*). The *h* is kept constant in our model experiments.

The isotopic composition of leaf water ($\alpha_{\text{lp}}$) is calculated using the following formulation (*77*)

$$\alpha_{\text{lp}}=\left[\alpha_{S}\alpha_{k}\left(1-h\right)+\alpha_{V}h\right]\alpha_{\text{eq}}$$ (2)

where $\alpha_{k}$ and $\alpha_{\text{eq}}$ are the kinetic and equilibrium fractionation factors, and h indicates the relative humidity. The $\alpha_{S}$ and $\alpha_{V}$ are the isotopic composition of (GPP-weighted) precipitation water and (GPP-weighted) water vapor, respectively, expressed in fractionation factor relative to *R*_sw_. The values of these fractionation factors are adopted from Blunier *et al.* (*38*).

The terrestrial respiratory fractionation factor ($\alpha_{\text{lr}}$) is calculated as a weighted average of isotope fractionation during O_2_ consumption via dark respiration in soil and in leaves, photorespiration, Mehler reaction, and respiration via alternative oxidase. The values of these fractionation factors are taken from Blunier *et al.* (*38*). The fraction of photorespiration is calculated as the fraction of C4 photosynthesis as we assume no photorespiration in C4-type vegetation. The fraction of C4 photosynthesis is inversely scaled to the CO_2_ concentrations between 0.275 (PI) and 0.39 (LGM) following Blunier *et al.* (*38*). The fraction of respiration via Mehler reaction and alternative oxidase are assumed to be constant at 10 and 2%, respectively.

The marine photosynthetic fractionation factor ($\alpha_{\text{op}}$) and the ln(δ^17^O + 1) / ln(δ^18^O + 1) slope ($\theta^{17/18}$) are prescribed to be 1.004 and 0.524, respectively. These values are obtained from the biological chamber experiments using different phytoplankton species [(*78*) and references therein]. The marine respiratory fractionation factor ($\alpha_{\text{or}}$) and the associated $\theta^{17/18}$ is taken as 0.9785 and 0.518, respectively. The $\alpha_{\text{or}}$ value is obtained by weighted average of respiratory fractionation factor in the surface ocean $\left(\alpha_{\text{ors}}=0.978\right)$ and that in the deep ocean ($\alpha_{\text{ord}}=0.988$). The fraction of marine respiration in the surface ocean is assumed to be 95% of the total respiration.

Following Blunier *et al.* (*38*), we prescribe the land productivity (*F*_lp_) using a polynomial regression curve relating GPP (in carbon) to CO_2_ from the transient simulation of LPJ-DGVM (*79*). The modeled GPP is converted to GOP using the relationship between the actual electron transport rate and the CO_2_ assimilation rate (*80*), following Eq. 4 of Blunier *et al.* (*38*).

Given the prescribed land productivity, the ocean productivity (*F*_op_) can be calculated using the Eq. 1

Fop=Flp[αlpxRswx−αlrxRatmxαorxRatmx−αopxRswx]+FstrRatmx(αstrx−1)αorxRatmx−αopxRswx (3)

where the superscript x denotes the rare oxygen isotopes, 17 or 18. The global productivity is then calculated as the sum of land and ocean productivity.

The input data for the ^17^Δ box model are taken from ice-core measurements and the published literature. For δ^18^O_atm_, we use measurements from the NEEM ice core (*48*). CO_2_ records are from the WAIS Divide ice core on its WD2014 timescale (*29*). For δ^18^O of seawater (δ^18^O_sw_), we scaled the absolutely dated relative sea-level reconstruction data from Red Sea (*81*) to 0 (modern) to 1‰ (LGM) to obtain high-resolution δ^18^O_sw_ record. All the data are linearly interpolated to each time step of ^17^Δ data. The ^17^Δ and δ^18^O_atm_ data from NEEM ice core were transferred to WD2014 timescale by CH_4_ correlation.

We obtain the modeled ^17^Δ and δ^18^O_atm_ curves (Fig. 2) using the following equation derived by rearranging Eq. 1

Ratmx=FopαopxRswx+FlpαlpxRswxForαorx+Flrαlrx+(1−αstrx)Fstr (4)

where superscript x represents 17 and 18, and we assume *F*_op_ = *F*_or_ and *F*_lp_ = *F*_lr_. The CO_2_-effect–only scenario is calculated by prescribing biosphere fluxes (*F*_lp_, *F*_op_), fractionation factors (α_lp_, α_lr_, α_op_, α_or_), and water isotopic ratios (*R*_sw_) to the values of period I.

### GCM experiments

The IPSL-CM5A2-VLR GCM (*42*) is composed of the LMDZ atmosphere model (*82*), the ORCHIDEE land surface and vegetation model (*83*), and the NEMO ocean model (*84*). NEMO (version 3.6) itself consists of the OPA dynamic ocean model, the LIM2 sea-ice model (*85*), and the PISCES-v2 marine biogeochemistry model (*64*). OASIS (*86*) is used to couple the models, and XIOS (*87*) handles input/output processing. LMDZ and ORCHIDEE share the same horizontal resolution of 3.75° × 1.875° (longitude × latitude) and LMDZ is discretized into 39 uneven levels in the vertical. NEMO has a nominal horizontal resolution of 2°, enhanced to 0.5° at the equator, and 31 vertical levels whose thickness varies from 10 m at the surface to 500 m at the bottom. NEMO uses a tripolar grid to overcome the North Pole singularity (*88*). This version of the IPSL model is essentially a retuned version of the IPSL-CM5A model (*89*) with reduced biases and better numerical performances (*42*). It has been applied extensively in the past years to simulate modern [e.g., (*90*)] and past climates [e.g., (*91*)], including Quaternary climates [e.g., (*92*)].

Here, we design new experiments to simulate the effects of the HS4 perturbation on the global biosphere productivity. Our control experiment features boundary conditions for 40 ka B.P. to provide a reference for global biosphere productivity just before the onset of HS4. To this end, we used the 40-ka land-sea mask and ice-sheet topography provided by the recent reconstruction of Gowan *et al.* (*93*). This new ice-sheet reconstruction is characterized by a smaller ice-sheet extent and hence higher global sea level compared to the 16-ka ice-sheet geometry that was used as an analog of Marine Isotope Stage 3 (MIS3) and MIS4 ice sheets in previous model simulations [e.g., (*94*)]. Other important boundary conditions for the control experiment include atmospheric CO_2_, CH_4_, and N_2_O concentrations of 200 ppmv, 450 ppbv, and 240 ppbv, respectively, and the LGM dust deposition from Albani *et al.* (*59*). The control simulation is run for 1100 years from the end of the previous MIS3 simulation under 46-ka boundary condition (*95*). A brief evaluation of the mean climate state of this new 40-ka simulation and its comparison to a reference IPSL-CM5A2-VLR preindustrial simulation is presented in the next section.

From our control simulation, we branch four 400-year perturbation experiments that are designed to investigate the effect of a Heinrich event on different known drivers of biosphere productivity changes. To this end, we sequentially cumulate the drivers affected by a Heinrich-like state: In the first experiment (Exp_FWF), a constant, uniformly distributed freshwater flux of 0.2 sverdrup is added to the North Atlantic Ocean between 40° and 80°N for a duration of 400 years to perturb the AMOC. In the second experiment (Exp_DUST), besides the same freshwater flux, we impose the H1 (16 ka B.P.) dust deposition field (*59*, *60*) instead of the LGM (21 ka B.P.) one (*59*, *60*). In our simulation, aeolian dust refers to atmospheric deposition of iron (Fe), silicate (Si), and phosphorous (P) minerals (*65*). The solubility of dust-borne Si and P is fixed at constant 7.5% (*96*) and 10% (*97*), respectively, whereas Fe solubility in the surface ocean is prescribed as spatially heterogeneous (fig. S20) (*98*). These solubility fields are held constant across all simulations. The difference between Exp_DUST and Exp_FWF therefore indicate the influence of dust deposition change on the global GOP. The third experiment (Exp_CO2) adds the effect of a slightly increased atmospheric CO_2_ (to 210 ppmv), mimicking the CO_2_ increase documented in the ice core record of Wendt *et al.* (*29*) across HS4. The difference between Exp_CO2 and Exp_DUST represents the effect of CO_2_ rise. Last, to examine the effect of vegetation dynamics, the last experiment (Exp_VFIX) is set to identical to Exp_CO2, but the vegetation cover is fixed to that obtained at the end of the control (Ctrl_40ka) simulation. Results presented and discussed here are the climatological average of years 201 to 400, unless otherwise specified.

In each simulation, terrestrial GOP is calculated directly by ORCHIDEE. We assume that GOP equals to the flux of O_2_ produced at photosystem II by splitting water molecules, before partially consumed by photorespiration during PCO cycle (*80*). Resulting GOP values show a near-linear relationship with GPPC with a slope of ∼2 (Fig. 3). The GOP-GPPC slope is insensitive to latitudes. The slope is greater than the typical value of photosynthetic quotient of ∼1.1 (moles O_2_) (moles C)^−1^. The GOP-GPPC difference reflects the photorespiratory O_2_ consumption. Marine GOP is calculated using the new and regenerated primary productivity obtained in PISCES by multiplying by the photosynthetic quotient and a factor of 2.3 to convert to GOP.

### 40-ka mean climate state

Surface diagnostics of the 40-ka simulation are presented in fig. S14. The mean annual 2-m temperature (T2m) varies from lower than 214 to greater than 297 K, over the interior east Antarctica and the tropical regions, respectively (fig. S14A). Globally averaged T2m in our Ctrl_40ka is 282.9 ± 0.1 K, which is 3.4° ± 0.1°C cooler than that obtained from PREIND experiment (fig. S14B), and thereby lying between the global mean surface temperature of the LGM and the PI. This is expected as the prescribed CO_2_ concentration and ice sheet cover are intermediate states between those of the LGM and the PI. Accordingly, regions of largest temperature differences in our 40-ka simulation compared to the PI are those that become covered by perennial ice sheets (northern North America), as is the case in the PMIP4 LGM-PI temperature difference (*92*).

The Ctrl_40ka experiment also results in a drier climate compared to PREIND, although an increase in precipitation rate is observed in several regions (fig. S14, C and D). The precipitation patterns in our Ctrl_40ka are also similar to the LGM (*92*) with an overall decrease in the high latitudes and the equatorial band (amplified over Indonesia) compared to PREIND (fig. S14D) (*92*). The global mean yearly total (liquid + solid) precipitation rate is reduced by 0.22 ± 0.01 kg m^−2^ day^−1^.

The globally averaged simulated global biosphere productivity (GOP) in Ctrl_40ka is 85% of that obtained in PREIND experiment. As expected from the large decrease in prescribed atmospheric CO_2_ and cooler and drier climate in Ctrl_40ka simulation, the terrestrial productivity declines substantially compared to PI (85% compared to PREIND; fig. S14, E and F). The decrease in terrestrial GOP occurs in NH high latitudes and tropical regions, where the model predicts substantial cooling and/or drying (fig. S14F). The marine productivity (82% compared to PREIND) exhibits an overall decline between 30°S and 30°N but increases in the Atlantic and Indian sectors of the Southern Ocean as well as in the North Atlantic and North Pacific gyres. In the low latitudes, the decrease in productivity is likely linked to reduced low-latitude upwelling under colder climates [e.g., (*87*)]. In the northern high latitudes, productivity increases in the North Atlantic because of the increased AMOC intensity and penetration depth in our Ctrl_40ka simulation compared to PI (fig. S15). These two characteristics of the AMOC are a known consequence of glacial climate forcing in many Earth System Models, in particular, IPSL-CM5A (*92*). In our Ctrl_40ka simulation, the more vigorous and deeper AMOC (fig. S15) is associated with deeper mixed-layer depth, which increases the vertical mixing of the upper ocean and remobilizes subsurface nutrients, enhancing productivity there. In the Atlantic and Indian sectors of the Southern Ocean, the large increase in dust deposition prescribed in Ctrl_40ka simulation is likely the dominant driver of the increased productivity in these iron-limited zones (*99*). Overall, the simulated 40-ka climate resembles an intermediate state between the full LGM climate conditions and the PI.

## Supplementary Materials

### This PDF file includes:

Supplementary Text Figs. S1 to S20 Tables S1 and S2 Data S1 References
