# Supplementary material for: Global biosphere productivity response to Atlantic Meridional Overturning Circulation collapse during Heinrich Stadial 4
Source: Sci Adv. 2026 Jul 15;12(29):eaea0455. doi: 10.1126/sciadv.aea0455 (PMC13440228; doi:10.1126/sciadv.aea0455)
Supplement: Supplementary file 1 [file sciadv.aea0455_sm.pdf]

Science Advances
AAAS

Supplementary Materials for

Global biosphere productivity response to Atlantic Meridional Overturning Circulation collapse during Heinrich Stadial 4

Ji-Woong Yang *et al.*

Corresponding author: Ji-Woong Yang, ji-woong.yang@lsce.ipsl.fr

Sci. Adv. **12**, eaea0455 (2026)
DOI: 10.1126/sciadv.aea0455

The PDF file includes:

Supplementary Text
Figs. S1 to S20
Tables S1 and S2
Data S1
References

Supplementary Text

Isotopic composition of source water in the box model

The modern fractionation factors for precipitation water ($^{18}\alpha_s$; equal to that of stem) and water vapor ($^{18}\alpha_v$) are 0.9921 and 0.9818, respectively (*101*, *102*). To account for the past variability of the isotopic composition of the source water, we introduce a scaling factor (γ), which is expressed as a form of fractionation factor. In our box model, the isotopic composition of source water (R_s) and water vapor (R_v) are calculated as follows where x stands for s or v and $\theta = 0.528$ (*102*):

$$^{18}R_x = {}^{18}\alpha_x \cdot \gamma(t) \cdot {}^{18}R_{sw}(t)$$

$$^{17}R_x = \left({}^{18}\alpha_x \cdot \gamma(t)\right)^{\theta} \cdot {}^{17}R_{sw}(t) = {}^{17}\alpha_x \cdot \gamma(t)^{\theta} \cdot {}^{17}R_{sw}(t)$$

As a consequence, the isotopic composition of the leaf water R_{lp} (*101*) is expressed as a function of time (t) and γ:

$$\begin{aligned} {}^{18}R_{lp}(t) &= \left[{}^{18}\alpha_s \cdot \gamma(t) \cdot {}^{18}\alpha_k \cdot (1-h) + {}^{18}\alpha_v \cdot \gamma(t) \cdot h\right] \cdot {}^{18}\alpha_{eq} \cdot {}^{18}R_{sw}(t) \\ &= {}^{18}\alpha_{lp}\gamma(t) \cdot {}^{18}R_{sw}(t) \end{aligned}$$

$$\begin{aligned} {}^{17}R_{lp}(t) &= \left[{}^{17}\alpha_s \cdot \gamma(t)^{0.528} \cdot {}^{17}\alpha_k \cdot (1-h) + {}^{17}\alpha_v \cdot \gamma(t)^{0.528} \cdot h\right] \cdot {}^{17}\alpha_{eq} \cdot {}^{17}R_{sw}(t) \\ &= {}^{17}\alpha_{lp}\gamma(t)^{\theta} \cdot {}^{17}R_{sw}(t) \end{aligned}$$

A box model can be formulated (*38*) for the isotopic composition of the troposphere which in steady state, $\frac{d}{dt}(M\,{}^{x}R_{atm}) = 0$, and modified for the time dependence of R_s and R_v writes as:

$$^{17}R_{atm}(t) = \frac{F_{lp}(t) \cdot {}^{17}\alpha_{lp}\left(\gamma(t)^{\theta}\right) \cdot {}^{17}R_{sw}(t) + F_{op}(t) \cdot {}^{17}\alpha_{op} \cdot {}^{17}R_{sw}(t)}{F_{lp}(t) \cdot {}^{17}\alpha_{lr}(t) + F_{op}(t) \cdot {}^{17}\alpha_{or} \cdot {}^{17}\alpha_{ge} + F_{str}(t) \cdot \left(1 - {}^{17}\alpha_{str}\right)}$$

$$^{18}R_{atm}(t) = \frac{F_{lp}(t) \cdot {}^{18}\alpha_{lp}(\gamma(t)) \cdot {}^{18}R_{sw}(t) + F_{op}(t) \cdot {}^{18}\alpha_{op} \cdot {}^{18}R_{sw}(t)}{F_{lp}(t) \cdot {}^{18}\alpha_{lr}(t) + F_{op}(t) \cdot {}^{18}\alpha_{or} \cdot {}^{18}\alpha_{ge} + F_{str}(t) \cdot \left(1 - {}^{18}\alpha_{str}\right)}$$

where $F_{lp}(t)$ and $\alpha_{lr}(t)$ are functions of CO_2 concentrations. The ocean productivity $F_{op}(t)$ is then derived from both $^{17}R_{atm}$ and $^{18}R_{atm}$:

$$\begin{aligned} F_{op}(t) &= \frac{\left[F_{lp}(t) \cdot {}^{17}\alpha_{lr}(t) + F_{str}(t) \cdot \left(1 - {}^{17}\alpha_{str}\right)\right] \cdot {}^{17}R_{atm}(t) - F_{lp}(t) \cdot {}^{17}\alpha_{lp}\left(\gamma(t)^{\theta}\right) \cdot {}^{17}R_{sw}(t)}{{}^{17}\alpha_{op} \cdot {}^{17}R_{sw}(t) - {}^{17}\alpha_{or} \cdot {}^{17}\alpha_{ge} \cdot {}^{17}R_{atm}(t)} \\ &= \frac{\left[F_{lp}(t) \cdot {}^{18}\alpha_{lr}(t) + F_{str}(t) \cdot \left(1 - {}^{18}\alpha_{str}\right)\right] \cdot {}^{18}R_{atm}(t) - F_{lp}(t) \cdot {}^{18}\alpha_{lp}(\gamma(t)) \cdot {}^{18}R_{sw}(t)}{{}^{18}\alpha_{op} \cdot {}^{18}R_{sw}(t) - {}^{18}\alpha_{or} \cdot {}^{18}\alpha_{ge} \cdot {}^{18}R_{atm}(t)} \end{aligned}$$

The F_{op} calculated from $^{17}R_{atm}$ and $^{18}R_{atm}$ are equal. At each time step t, the above equation can be solved in two ways either by keeping $\gamma = 1$ and solving for h equaling similar meteoric conditions as today or by keeping h constant and solving for γ. These scenarios are not mutually exclusive. A solution in which both h and γ vary is possible, and perhaps even likely. To solve the equation, we prescribe F_{lp} and F_{str}, both of which are linked to the CO_2 concentration as described in Blunier *et al*. (*38*) and Yang *et al*. (*41*), respectively. With the solution for h or γ the isotopic composition of leaf water and F_{op} are calculated.

In previous studies (*38*, *41*) gross oxygen productivity (GOP)-weighted relative humidity was varied while keeping the isotopic composition of source water constant. Here we favor the variable $\gamma(t)$ case. While no observational evidence is available so far, freshwater hosing experiments under Last Glacial Maximum (LGM) boundary conditions using IPSL-CM4 Global Climate Model (GCM) show almost constant relative humidity (*32*). Also, our new experiments under 40 ka boundary conditions with the fully-coupled IPSL-CM5A2-VLR GCM indicate no significant change in relative humidity. Given those results, we keep relative humidity constant and turn to a time-dependent fractionation of precipitation water and of water vapor.

Figure S1 shows the resulting $^{18}\varepsilon_{stem}$, and $\delta^{18}O$ of photosynthetic water ($\delta^{18}O_p$). The $^{18}\varepsilon_{stem}$ represents the isotopic fractionation effect of the photosynthetic source water from the global seawater. What is relevant to our box model approach is the GOP-weighted average value (temporally and spatially). Our box model predicts a gradual enrichment of $\delta^{18}O_p$ through the Period II in parallel to the ice-core $\delta^{18}O_{atm}$ records. This is consistent with Reutenauer *et al*.'s study (*32*) showing that the $\delta^{18}O_{atm}$ enrichment during HS is almost entirely explained by changes in $\delta^{18}O$ of precipitation water.

Figure S2 compares the global GOPs calculated by the two approaches. Both approaches result in an elevated GOP in Period II compared to Period I. Varying relative humidity shows a slightly drier Period II. Monte-Carlo simulations calculating GOP from the CO_2-effect-corrected $^{17}\Delta$ curve and its probability distribution (red line and shaded area) yield less than 2% and 9% of solutions showing equal or lower GOP in Period II compared to Period I when varying humidity or gamma, respectively. This consistency reinforces the robustness of our finding that global biosphere productivity increases in Period II relative to Period I.

<u>Sensitivity to biosphere disequilibrium</u>

Our box model presumes terrestrial and marine biospheres in steady state, such that $F_{lp} = F_{lr}$ and $F_{op} = F_{or}$. However, the terrestrial and marine biosphere need not be in exact steady state. Below we show that plausible departures from equilibrium are small in magnitude and thus have a negligible effect on the atmospheric $^{17}\Delta$ budget.

For the modern era (1990 – 2020), published estimates of global terrestrial GPP range from 108 to 190 PgC $year^{-1}$ (*103* and references therein), while global net ecosystem exchange (NEE = ecosystem respiration minus GPP) spans from -1.6 to -3.3 PgC $year^{-1}$ (*104*, *105*). These estimates imply that the ecosystem respiration differs from GPP by only a few percent, corresponding to a terrestrial O_2-flux ratio F_{lp}/F_{lr} of about 97 to 99%. Using the box-model equations and modern gross O_2 productivity in terrestrial- and marine biosphere (2.34 and 1.08 Pmol O_2 $year^{-1}$ respectively), the resulting perturbation in modelled $^{17}\Delta$ remains below 1 ppm. This magnitude lies well below the analytical uncertainty and detection limit for the NEEM $^{17}\Delta$

record. Because modern fluxes include anthropogenic perturbations, we consider this calculation as a conservative upper bound. Independent evidence from deconvolution of ice-core CO_2 records yields net land-atmosphere carbon flux < 0.5 PgC $year^{-1}$ over the year 1400 and 1700 (*106*), indicating an even closer equilibrium under pre-industrial conditions.

The calculation above also assumes that atmospheric O_2 is in equilibrium, such that $F_{lp} + F_{op} = F_{lr} + F_{or}$. Atmospheric O_2 (in partial pressure) does show a secular decline over the last 800,000 years, with a trend of about -0.2% per million years (*107*). This rate leads to a negligible change on millennial timescales. This justifies our assumption that treats atmospheric O_2 as steady state.

Sensitivity to isotope fractionation in the stratosphere

Our box model does not include photochemical reactions of oxygen species in the stratosphere. The isotope fractionation effect in the stratosphere ($\epsilon_{str} = \alpha_{str} - 1$) is parameterized, scaling linearly with the past atmospheric CO_2 concentration. However, the ε_{str} value in the past is unknown and may not strictly follow CO_2 scaling. A sensitivity test with constant ε_{str} over periods I and II results in no GOP change (fig. S3). This result is not surprising since no compensation for CO_2-effect is needed by the global biosphere, implying that changes in ε_{str} between Period I and Period II play a key role in the interpretation of the ice-core $^{17}\Delta$ record as a GOP proxy. Below we discuss potential change in ε_{str} (other than CO_2 effect) and how likely it is.

The primary source of mass-independent fractionation (MIF) of oxygen isotopes is O_3 formation (*e.g., 108-110*). Any change in gross O_3 formation rate would therefore influence the $^{17}\Delta$ signal of stratospheric O_2. Below, we estimate a potential change in the gross O_3 formation during the studied interval. The estimated effect is small, and would slightly increase the global biosphere productivity during Period II to compensate the stronger depletion of $^{17}\Delta$ of O_2 in the stratosphere. Therefore, our main conclusions are unchanged. Below we explain in more detail.

To quantify this effect, we evaluate the gross O_3 formation rate (P_{O3}) in the sunlit upper stratosphere (~40 km), using an oxygen-only Chapman cycle:

R1 (O_2 photolysis): $O_2 + hv \rightarrow O + O$ (rate = $2J_{O_2}[O_2]$)
R2 (O_3 formation): $O + O_2 + M \rightarrow O_3 + M$ (rate = $k_1[O][O_2][M]$)
R3 (O_3 photolysis): $O_3 + hv \rightarrow O_2 + O$ (rate = $J_{O_3}[O_3]$)
R4 (O_3 destruction): $O + O_3 \rightarrow 2O_2$ (rate = $k_2[O][O_3]$)

where mass balance of the atomic oxygen is:

$$\frac{d[O]}{dt} = 2J_{O_2}[O_2] + J_{O_3}[O_3] - k_1[O][O_2][M] - k_2[O][O_3]).$$

The steady-state solution for the atomic-oxygen concentration is:

$$[O] = \frac{2J_{O_2}[O_2] + J_{O_3}[O_3]}{k_1[O_2][M] + k_2[O_3]}.$$

The steady-state assumption is valid in the upper stratosphere, where odd-oxygen photochemistry is rapidly equilibrated (*e.g., 111*). We focus on this region because the production of energetically excited oxygen ($O(^1D)$), which transfers the MIF signal of O_3 to CO_2, becomes important at higher altitudes since the $O(^1D)$ channel of O_3 photolysis requires shorter-wavelength UV (*112*, *113*).

In the sunlit upper stratosphere (~40 km), the O-atom production term from O_3 photolysis dominates that from the O_2 photolysis, *i.e.,* $J_{O_3}[O_3] \gg 2J_{O_2}[O_2]$, because O_2 photolysis is restricted to shorter wavelength (< 240 nm) than O_3 photolysis. In addition, $k_1[O_2][M]$ term dominates $k_2[O_3]$ due to larger abundances of O_2 and M than ozone. Below we evaluate these terms to verify our assumption.

At 40 km, the TUV model (*114*) yields $J_{O_2} \approx 4.61 \times 10^{-10}\text{s}^{-1}$ and $J_{O_3} \approx 2.88 \times 10^{-3}\text{s}^{-1}$ for a solar zenith angle of 0° and an overhead ozone column of 10 DU. The numerical experiments using the Whole Atmosphere Community Climate Model 6 (WACCM6) with preindustrial (PI) and LGM boundary conditions yield the respective O_3 mixing ratio of 6.89 and 6.63 ppm at 40 km (*73*). We assume the O_2 mixing ratio is constant at 21%. Using a typical number density of $8.3 \times 10^{16}\text{cm}^{-3}$, $[O_2]$ and $[O_3]$ are calculated as $1.74 \times 10^{16}\text{cm}^{-3}$ and $5.5 - 5.7 \times 10^{11}\text{cm}^{-3}$. Taken together, $J_{O_3}[O_3] \approx 1.6 \times 10^{9}\text{cm}^{-3}\text{s}^{-1}$ is greater than $2J_{O_2}[O_2] \approx 1.6 \times 10^{7}\text{cm}^{-3}\text{s}^{-1}$ by two orders of magnitude.

The relevant reaction rate constants are temperature dependent (*55*):

$$k_1(T) = (6.1 \times 10^{-34}) \times \left(\frac{T}{298\text{ K}}\right)^{-2.4} \text{cm}^6\text{ molecule}^{-2}\text{ s}^{-1}$$

$$k_2(T) = (8.0 \times 10^{-12}) \times \exp\left(\frac{-2060}{T}\right) \text{cm}^3\text{ molecule}^{-1}\text{ s}^{-1}$$

At $T = 250$ K, $k_1 = 9.3 \times 10^{-34}\text{ cm}^6\text{ molecule}^{-2}\text{ s}^{-1}$ and $k_2 = 2.1 \times 10^{-15}\text{ cm}^3\text{ molecule}^{-1}\text{ s}^{-1}$, resulting in a ratio of $k_1[O_2][M]/k_2[O_3] \approx 8 \times 10^2$.

Using these two approximations, the atomic-oxygen concentration can be simplified to:

$$[O] \approx \frac{J_{O_3}[O_3]}{k_1[O_2][M]},$$

and substituting this expression into the gross O_3 formation rate yields:

$$P_{O_3} = k_1[O][O_2][M] \cong J_{O_3}[O_3].$$

The gross O_3 production can be approximated as the product of O_3 photolysis rate constant and O_3 concentration. Neither term is necessarily constant over the studied interval; for example, the photolysis rate constant is sensitive to temperature and overhead O_3 column, and the O_3 concentration varies with changes in photochemical equilibrium. A relative difference between Period I and Period II of P_{O_3} can be written as follows (for small perturbations):

$$\frac{\Delta P_{O_3}}{P_{O_3}} \approx \frac{\Delta J_{O_3}}{J_{O_3}} + \frac{\Delta[O_3]}{[O_3]}.$$

Assuming that temperature and overhead O_3 column are the primary controls on J_{O3}, the fractional changes in J_{O3} can be written as:

$$\frac{\Delta J_{O_3}}{J_{O_3}} \approx \left(\frac{\partial \ln J_{O_3}}{\partial T}\right) \Delta T + \left(\frac{\partial \ln J_{O_3}}{\partial O_3^{col}}\right) \Delta O_3^{col}.$$

The sensitivities of J_{O3} to the temperature and overhead ozone column are computed with the TUV model (fig. S4). Changes in temperature and overhead ozone column between Period I and Period II are estimated by linearly scaling the PI – LGM anomalies from the WACCM6 simulations (*73*):

$$\Delta T \approx \left(\frac{14 \text{ ppm}}{90 \text{ ppm}}\right) \times (252.9 \text{ K} - 258.7 \text{ K}) = -0.9 \text{ K, and}$$

$$\Delta O_3^{col} \approx \left(\frac{14 \text{ ppm}}{90 \text{ ppm}}\right) \times (11.14 \text{ DU} - 10.53 \text{ DU}) = 0.09 \text{ DU}.$$

Similarly, the relative change in $[O_3]$ at 40 km is calculated by linearly scaling WACCM6 outputs by CO_2 changes. The resulting relative change in P_{O3} is then computed as follows:

$$\frac{\Delta P_{O_3}}{P_{O_3}} \approx (3.7 \times 10^{-4} \text{ K}^{-1}) \times (-0.9 \text{ K}) + (-1.3 \times 10^{-2} \text{ DU}^{-1}) \times (0.09 \text{ DU}) + \left(\frac{14 \text{ ppm}}{90 \text{ ppm}}\right) \times \left(\frac{6.9-6.6 \text{ ppm}}{6.6 \text{ ppm}}\right) = 0.006.$$

The calculation above indicates that gross O_3 formation is accelerated by ~0.6% in Period II relative to Period I. Because O_2 becomes depleted in $^{17}\Delta$ during O_3 formation, a faster O_3 formation implies that the stratospheric O_2 would become more depleted (*i.e.,* a smaller α_{str} since $\alpha_{str} < 1$) solely due to faster O_3 formation in Period II. Based on mass-balance of tropospheric $^{17}\Delta$ of O_2, this would imply a further increase in global biosphere productivity in Period II compared to our default scenario.

We therefore conclude that the slight change in O_3 formation and associated MIF would marginally increase the inferred global biosphere productivity in Period II relative to our default scenario, and therefore does not affect our main conclusions. Obviously, we note that this estimate relies on several assumptions and carries substantial uncertainty. A more comprehensive study using isotope-enabled climate-chemistry model is required to elucidate changes in oxygen-isotope MIF during abrupt climate events.

In addition, because $O(^1D)$ is the principal conduit through which the ^{17}O anomaly generated during O_3 formation is transferred out of the Chapman cycle, its lifetime is a key control on the $^{17}\Delta$ signal of stratospheric O_2. In the upper stratosphere, $O(^1D)$ is produced primarily by O_3 photolysis and removed by collisional deactivation (quenching) with ambient air molecules.

Below we briefly discuss how these production and loss terms may respond to the expected upper-stratospheric changes during Period II.

The rate constant for the O_3 destruction reaction (reaction R4) is temperature dependent (see k_2 above). Cooling in the upper stratosphere would slow this reaction, increasing the abundance of O_3. Higher O_3 availability would, in turn, increase the production of $O(^1D)$ via O_3 photolysis and hence enhance the quenching rate of $O(^1D)$ with CO_2.

$O(^1D)$ produced by O_3 photolysis is quenched to $O(^3P)$ by collisions with surrounding molecules. The major collisional partners are N_2, O_2 and CO_2 (*e.g.*, *54*), but only the quenching by CO_2 enables O-isotope exchange and thus "extracts" the ^{17}O anomaly from the Chapman cycle. The $CO_2 + O(^1D)$ rate depends on temperature (*55*):

$$k(T) = (7.5 \times 10^{-11}) \times \exp\left(\frac{115}{T}\right) \text{ cm}^3 \text{ molecule}^{-1} \text{ s}^{-1}$$

A slight cooling (see above) in the upper stratosphere therefore increases the rate constant, accelerating $O(^1D)$ transfer to CO_2 and likely enhancing the ^{17}O depletion in the O_2 reservoir. Any additional ^{17}O depletion of stratospheric O_2 would require a stronger biosphere productivity during Period II to match the observed ice-core $^{17}\Delta$ record, so it does not alter our conclusion. Of course, our argument depends on upper-stratosphere temperature during HS4, which is poorly constrained. Nevertheless, the rise in greenhouse gases (CO_2 and CH_4) during Period II, demonstrated by ice-core records, is more consistent with upper-stratospheric cooling than warming.

Ice-core measurements further suggest a long-term decrease in atmospheric O_2 over the last 800,000 years, with a rate of about -0.2% per million years (*107*). To first order, a smaller stratospheric O_2 pool would reduce O_3 production by limiting the source of atomic oxygen from O_2 photolysis. It would also reduce quenching of $O(^1D)$ by O_2, which could increase the chance of quenching by CO_2. These two effects act in opposite directions on the net isotopic imprint in stratospheric O_2, and the combined response is difficult to assess without more concrete constraints on the stratospheric state during the study interval. However, the expected change in O_2 during HS4 is small, and therefore we prefer our default assumption of steady-state O_2.

<u>Sensitivity to stratosphere-to-troposphere influx</u>
Another unknown is past changes in the influx of stratospheric O_2 into the troposphere (F_{str}). A previous modelling study shows that with rising atmospheric CO_2 concentrations over the historical period the age of air (at 25 km altitude) decreases, implying an acceleration of Brewer-Dobson Circulation (BDC) (*53*). We therefore scale F_{str} to CO_2 changes between 84.7% (CO_2 = 190 ppm) and 100% (CO_2 = 280 ppm) based on a climate-chemistry model simulation under LGM boundary conditions (*115*). Doing so, the abrupt CO_2 jump during HS4 leads to an increased influx of stratospheric O_2 with depleted $^{17}\Delta$ to the troposphere, implying that GOP reconstruction could be altered if the sensitivity of F_{str} to CO_2 changes. A sensitivity test of a constant F_{str} (fixed to the average value over Period I) leads to a weaker GOP rise in Period II compared to the default scenario. On average the observed relative change in GOP is only 1.2% (fig. S3). However, the triple oxygen isotopic ratio measurements in ice-core NO_3 suggest that

BDC is accelerated under cold climate due to enhanced latitudinal gradient of sea-surface temperature (SST) (*e.g., 58*). If this is the case, a stronger BDC is more likely during HS4 because weakening AMOC causes a severe cooling around the North Atlantic and hence greater latitudinal SST gradient, and is qualitatively consistent with our initial assumption. We therefore consider our default scenario more likely.

<u>Sensitivity to prescribed land productivity</u>

In our default scenario, we prescribe the land productivity in the same manner as Blunier *et al.* (*38*), where the authors use a polynomial regression of simulated GPPC (*79*) to CO_2 concentration. The regression curve yields an increase in land GOP by ~2% in response to a CO_2 increase from 200 to 210 ppm. Our simulations using IPSL-CM5A2-VLR show an even stronger increase in land GOP of ~5% for a CO_2 increase from 200 to 210 ppm (experiments Exp_DUST and Exp_CO2). In order to examine the sensitivity of our box model calculating ocean GOP to prescribed land GOP, we carried out experiments using three different scenarios of prescribed land GOP. The double-sensitivity curve is obtained using the following equation:

$$\text{Land biosphere GPPC (mol C yr}^{-1}\text{)} = -2.34 \times 10^{11} \times [\mathrm{CO_2}]^2 + 1.55 \times 10^{14} \times [\mathrm{CO_2}] - 1.26 \times 10^{16}$$

where $[\mathrm{CO_2}]$ represents the CO_2 concentration in ppm. The land GOP curves used for sensitivity tests are shown in Figure S5. Given $^{17}R_{atm}$ and $^{18}R_{atm}$ (from ice-core $^{17}\Delta$ and $\delta^{18}O_{atm}$), the box model solutions yield a reduction of marine GOP in response to an enhancement in prescribed land GOP (fig. S5).

An increase in prescribed land GOP and concomitant decrease in marine GOP reduces the ocean-to-land GOP ratio (GOP_{oce}/GOP_{land}) and hence the $^{17}\Delta$ signature of photosynthetic O_2 produced in the global biosphere (fig. S5). A lower global biosphere $^{17}\Delta$ is compensated by a stronger GOP. The double-sensitivity scenario yields a 2.4% increase in global GOP in Period II (relative to Period I) compared to 1.9% in the default scenario (fig. S5).

In steady state, the reduced GOP_{oce}/GOP_{land} also affects the respiratory fractionation of ^{18}O, because of weaker terrestrial respiratory fractionation effect (~18‰) than that of marine biosphere (~21.5‰) prescribed in the box model. Given ice-core $\delta^{18}O_{atm}$ record, this 'deficit' of ^{18}O enrichment is compensated by an enrichment of ^{18}O in the source water (fig. S5).

<u>Sensitivity to ^{17}O-anomaly of precipitation water</u>

Another factor affecting the calculation of GOP is the ^{17}O anomaly of precipitation water as it affects the ^{17}O anomaly of photosynthetic O_2. What is relevant here is the GOP-weighted average value. Due to the lack of direct observations, the GOP-weighted ^{17}O anomaly of precipitation water is kept constant in the default scenario. A sensitivity test in which the ^{17}O anomaly of precipitation water is reduced by 15 ppm over this interval results in even stronger biosphere productivity compared to the default scenario (fig. S6), implying that the CO_2-effect could be offset by an enrichment of the ^{17}O anomaly of precipitation by ~8 ppm without requiring a change in GOP. Our rationale for the adopted scenario for ^{17}O anomaly of precipitation is discussed in the following section.

Potential shift of ^{17}O-anomaly of precipitation water

The past global distribution of the ^{17}O anomaly of precipitation water ($^{17}O_{excess}$) remains poorly understood. The few available measurements from polar ice cores may not be representative of the global trend. We therefore use modern seasonal variability (December-January-February vs. June-July-August) as an empirical benchmark for the response of global $^{17}O_{excess}$ to a southward migration of the ITCZ.

We first analyse monthly outputs from the isotope-enabled general circulation model LMDZ-iso (*116*) to estimate seasonal (DJF vs. JJA) variability in the $^{17}O_{excess}$ of global precipitation (fig. S7). The model simulation follows the AMIP protocol for the year of 2005-2006 (*116*). Combined with gridded monthly-mean global GPP dataset (FLUXCOM; *117*), we compute the seasonal variations of GPP-weighted $^{17}O_{excess}$ (fig. S7). We then compare this to the centroid latitude of GPP, also termed the terrestrial oxygenesis equator (TOE), calculated following the definition given in Seltzer *et al*. (*50*). TOE reaches its northernmost position in July and its southernmost position in January (fig. S8A). In contrast, GPP-weighted $^{17}O_{excess}$ shows a distinct seasonal cycle, with a maximum value of 24.3 ppm in March and a minimum of 18.3 ppm in September (fig. S8A). A cross plot of GPP-weighted $^{17}O_{excess}$ versus TOE latitude reveals a moderate anti-correlation ($R^2 = 0.3$) with a slope of -0.08 ppm per degree latitude (fig. S8B). It seems that the GPP-weighted $^{17}O_{excess}$ is anti-correlated with TOE with a ~2-month delay. The regression improves when GPP-weighted $^{17}O_{excess}$ is shifted forward by 2 months, yielding $R^2 = 0.9$ and a slope of -0.14 ppm per degree latitude (fig. S8B). In addition, we compute GPP-weighted $^{17}O_{excess}$ using monthly $^{17}O_{excess}$ measurements from the Global Network of Isotopes in Precipitation (GNIP) dataset (*118*; fig. S9). Regression analysis that accounts for the reported analytical uncertainty of $^{17}O_{excess}$ yields a slope of -0.03 ± 0.02 ppm per degree latitude (fig. S10).

We use these regression slopes to estimate the expected change in $^{17}O_{excess}$ between Period I and Period II. The mean TOE latitude in the perturbation experiment (Exp_CO2) is ~2.6° farther south than in the control experiment (Ctrl_40ka) (fig. S11). A 2.6° southward displacement of TOE latitude therefore corresponds to an increase in GPP-weighted $^{17}O_{excess}$ of only ~0.2 to 0.4 ppm (LMDZ $^{17}O_{excess}$), or 0.07 ± 0.05 ppm (GNIP $^{17}O_{excess}$). These values are small and should have little influence on our box-model results, and do not affect the main conclusion of the manuscript. This evidence supports our choice of constant $^{17}O_{excess}$ as the default scenario.

Sensitivity to C4 photosynthesis fraction

The fraction of C4 photosynthesis also influences our GOP calculations by altering the fraction of photorespiration and, consequently, the terrestrial respiratory fractionation factor. In the default scenario, the C4 fraction is scaled to CO_2 concentrations between 0.39 (LGM) and 0.275 (preindustrial), following Blunier *et al*. (*38*). This assumption results in an abrupt decline in the C4 fraction following the CO_2 increase. Maintaining a constant C4 fraction leads to a slight reduction in GOP during Period II, with 17% of 3000 Monte Carlo simulations showing a GOP decline in this period (fig. S6). However, recent ice-core measurements of stable CH_4 isotopes suggest increased biomass burning associated with the Heinrich Event (*28*). Such a scenario could shift the balance between C3 and C4 photosynthesis, as C4 plants (mainly in the tropics) are likely more susceptible to wildfire. This would further reduce C4 photosynthesis. Thus, we consider a constant C4 contribution unlikely and conclude that any variations in C4 fraction between Periods I and II have a minor effect on GOP reconstruction.

Sensitivity to surface temperature

Soil- and marine respiratory isotopic effects can be altered by temperature change in response to HS4. According to our GCM simulations, the weighted (both in spatial and temporal) average of land surface temperature (Temp_sol) for land GOP differs by less than 1 degree between the control and freshwater perturbation experiments (slight warming). Similarly, the weighted average of the mean ocean temperature over the surface 100 meters for marine primary productivity differs by less than 1 degree between the two experiments (slight warming). Land- and ocean surface warming by 1 degree would result in a decrease in respiratory $^{18}\alpha_{lr}$ values to 0.98379 (dark soil) and 0.97789 (ocean), while increasing the lambda value of marine respiration to 0.51819 (*38*, *107*). These changes have little effect on GOP calculation. In addition, the air-ocean equilibrium fractionation factors ($^{17}\alpha_{eq}$ and $^{18}\alpha_{eq}$) are functions of water temperature. Following the relationship by Luz and Barkan (*119*) and Nicholson *et al*. (*120*), the ocean surface warming by 1 degree induces decreases in $^{17}\alpha_{eq}$ and $^{18}\alpha_{eq}$ by 2 and 5×10^{-6}, respectively, and has little effect on reconstructed GOP.

Sensitivity of modeled terrestrial GOP to prescribed CO_2

Our ESM simulations show that the CO_2 fertilization effect compensates the GOP reduction caused by AMOC slowdown and associated changes. However, the modelled terrestrial GOP is model dependent, and thus it can affect our conclusion. In a multi-model evaluation (*121*), ORCHIDEE indeed exhibits a relatively high sensitivity of GPP to rising CO_2 (β_{GPP}) compared to several other DGVMs (*e.g.,* LPJ, LPJ-GUESS, TRIFFID, SDGVM, OCN, HYLAND, CLM4C, CLM4CN, VEGAS). However, this does not mean that ORCHIDEE's GPP is unreliable. It mainly reflects differences among models in how key land processes and feedbacks are represented. ORCHIDEE is a process-based DGVM that includes detailed representations of photosynthesis and stomatal regulation, vegetation dynamics, and soil water and carbon cycling (*e.g., 83*). In ORCHIDEE, C3 photosynthesis follows the biochemical model of Farquhar *et al.* (*122*), and C4 photosynthesis is represented based on the formulation of Yin and Struik (*37*). ORCHIDEE GPP has also been evaluated against observation-based constraints across spatial scales from regional to global, and across temporal scales from diurnal variability to seasonal cycle and annual mean (*123*).

To test the sensitivity of our conclusions to uncertainty in the CO_2 response, we conducted an additional AMOC-perturbation experiment with CO_2 fixed at 214 ppm, instead of 210 ppm in Exp_CO2. Together with global terrestrial GOP values from Exp_DUST (CO_2 = 200 ppm) and Exp_CO2 (CO_2 = 210 ppm), we compute the response of GOP to rising CO_2, β_{GOP} (fig. S12). A linear regression gives β_{GOP} = 0.11 Pmol O_2 yr^{-1} ppm^{-1}. For a 10-ppm CO_2 increase, reducing β_{GOP} by 33% lowers terrestrial GOP by 0.4 Pmol O_2 yr^{-1} relative to Exp_CO2 and yields a global GOP of 29.3 ± 0.4 Pmol O_2 yr^{-1}. This value is slightly below Ctrl_40ka (29.6 ± 0.4 Pmol O_2 yr^{-1}), but the difference remains small. In addition, Exp_CO2 prescribes only a 10-ppm rise in CO_2, whereas recent ice-core records indicate a larger rise of ~14 ppm during HS4 (*29*). Scaling the reduced β_{GOP} to a 14-ppm CO_2 increase relative to Ctrl_40ka yields a global GOP of 29.6 ± 0.4 Pmol O_2 yr^{-1}, which is indistinguishable from Ctrl_40ka. Even when we reduce the beta-GOP, the conclusion remains the same. CO_2 fertilization offsets most of the simulated global GOP reduction under AMOC perturbation.

Validation of simulated vegetation

We conduct our GCM experiments using a dynamic global vegetation model (DGVM) and validate the simulated vegetation by comparing it with available palynological data (*e.g., 65*). To facilitate this comparison, we first converted 11 plant functional types (PFTs) into 9 mega-biomes. The classification criteria and corresponding mega-biomes are summarized in Table S1. We compare the simulated vegetation in our Ctrl_40ka experiment with the pollen assemblage at GI 8, as a comprehensive pollen data compilation for 40 ka BP is not available (*65*). Overall, the simulated vegetation cover in the Ctrl_40ka experiment aligns well with pollen data, except along the western coast of America and in central Europe.

In America, the simulated vegetation on the east coast agrees well with pollen data. On the west coast, the pollen records indicate a dispersion of boreal- and temperate forests, while the model simulates primarily 'Open Sclerophyll Forest/Woodlands' and 'Herbaceous and Shrublands', which are assigned a lower tree fraction than forest-type biomes (Table S1). This suggests that discrepancies between our DGVM and pollen data in this region arise from an underestimation of tree species in the model.

In East Asia, simulated vegetation cover closely matches pollen assemblages, except for one site in Southeast Asia where the model simulates 'Temperate Forest' instead of 'Warm Temperate Forest,' likely due to an underestimation of tropical tree species.

In Australia and New Zealand, the model simulates 'Bare Soil' and 'Herbaceous and Shrublands' in the interior of Australia, 'Tropical Savannah' and 'Open Sclerophyll Forest/Woodlands' along the northern coast, and 'Herbaceous and Shrublands' with 'Open Sclerophyll Forest/Woodlands' along the southern coast. However, it fails to capture tropical forest along the northern coast and tropical savannah in the south. Additionally, the model simulates 'Warm Tropical Forest' on the southern coast of Australia and in northern New Zealand, which is not reflected in pollen data.

In southern Europe, the model and pollen data are in good agreement, both showing a dominance of 'Open Sclerophyll Forest/Woodlands' and 'Herbaceous and Shrublands.' However, in central and eastern Europe, the model simulates 'Temperate Conifer and Broadleaf Forest,' whereas pollen data indicate a dominance of 'Herbaceous and Shrublands.'

Overall, we conclude that the model represents vegetation cover reasonably well in the Ctrl_40ka experiment, albeit with some spatial discrepancies. However, the pollen records are highly localized, limiting their ability to validate the simulation across the entire land surface. Notably, in South America—particularly in Amazonia—available pollen data provide little insight into this region. Our Ctrl_40ka simulation does not show well-developed tropical forest in the Amazon, likely due to a known bias in the IPSL-CM5A2-VLR model, which underestimates precipitation and forest cover over South America (*42*).

A comparison of vegetation simulated in the Exp_CO2 experiment with pollen records from GS 8/9 reveals several discrepancies. In western North America, the model fails to reproduce boreal and temperate forests indicated by pollen data, whereas the simulated temperate forest in eastern North America aligns well with observations (fig. S18). Similarly, along the western coast of

South America, the model does not develop tree species (temperate conifer and broadleaf forests), instead simulating a dominance of herbaceous species.

In Europe, all data indicate a prevalence of ‘Herbaceous and Shrublands,’ yet the model simulates a widespread distribution of ‘Open Sclerophyll Forest/Woodlands.’ In Africa, the model predicts a mixture of tropical and temperate forests, whereas pollen records suggest a dominance of ‘Open Sclerophyll Forest/Woodlands’ and ‘Herbaceous and Shrublands.’ This discrepancy is likely due to an overestimated tree fraction, possibly linked to excessive simulated precipitation, as noted in pre-industrial experiments (*42*).

In Australia, the model fails to simulate tropical and temperate forests in the northern and eastern coastal regions. In contrast, the simulated vegetation cover in East Asia closely matches pollen assemblages. Overall, the Exp_CO2 experiment produces less coherent vegetation patterns compared to the Ctrl_40ka experiment.

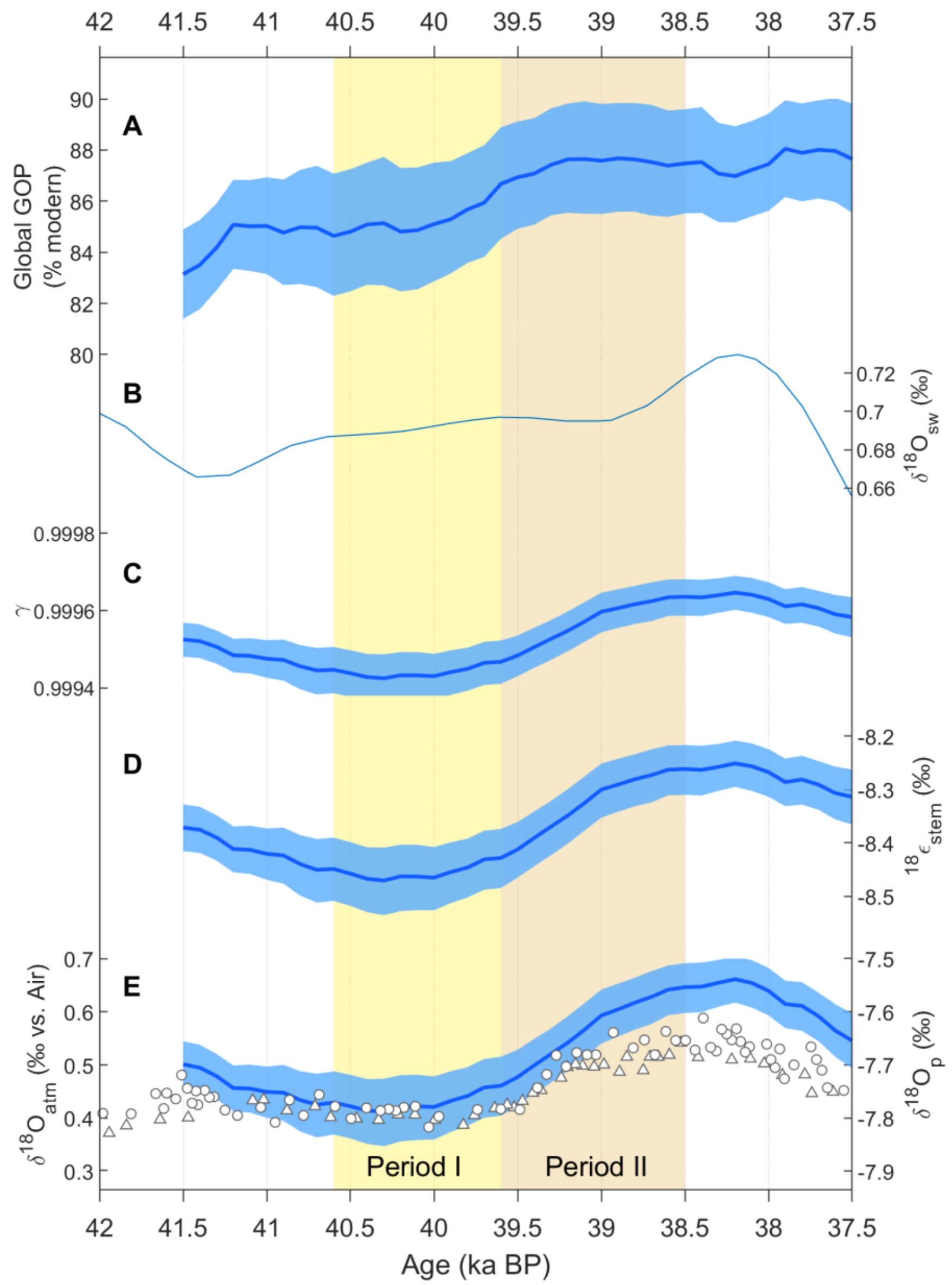

Fig. S1. Isotopic composition of the source water modelled in the box model. This figure shows the results of the "source-water approach" in our box model. (**A**) Reconstructed gross oxygen productivity (GOP) in the default scenario. (**B**) The prescribed $\delta^{18}O_{sw}$ curve obtained by scaling the Red Sea relative sea-level record (*81*). (**C**) The gamma (γ) values calculated in the box model. (**D**) The GOP-weighted isotopic fractionation effect (expressed as $\boldsymbol{\varepsilon_{stem}} = \boldsymbol{\alpha_{stem}} - \mathbf{1}$) of precipitation water consumed by terrestrial plants versus global ocean water. (**E**) The isotopic ratio of precipitation water calculated in the box model and ice-core $\delta^{18}O_{atm}$ records (circles: NEEM; triangles: Siple Dome).

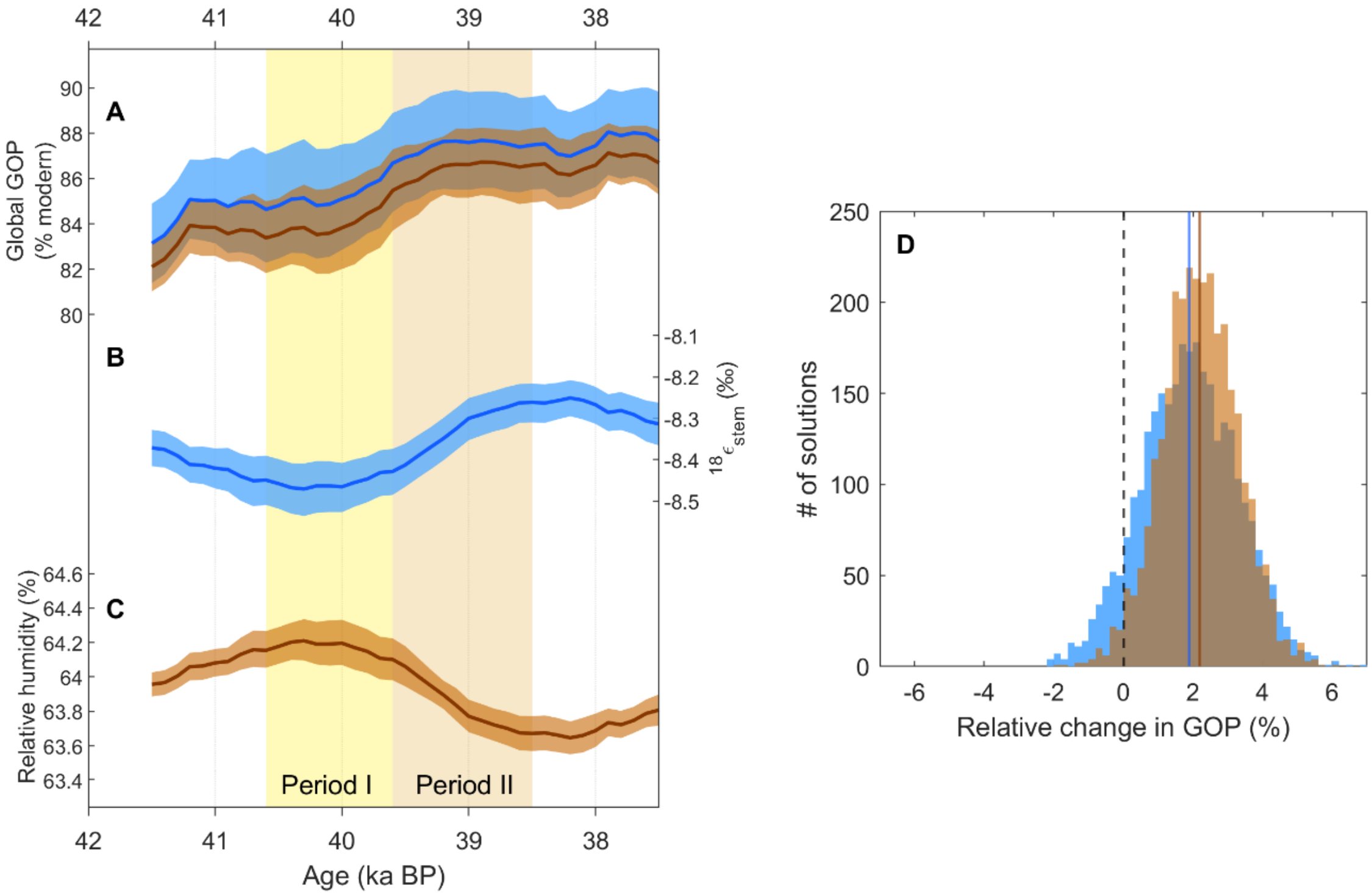

Fig. S2. Comparison of the source-water approach (blue) and the relative-humidity approach (brown). (**A**) gross oxygen productivity (GOP) records from both approaches. (**B**) The isotopic discrimination of GOP-weighted precipitation water. (**C**) The GOP-weighted relative humidity calculated in the box model. (**D**) The histogram of relative difference of GOP in Period II compared to that in Period I.

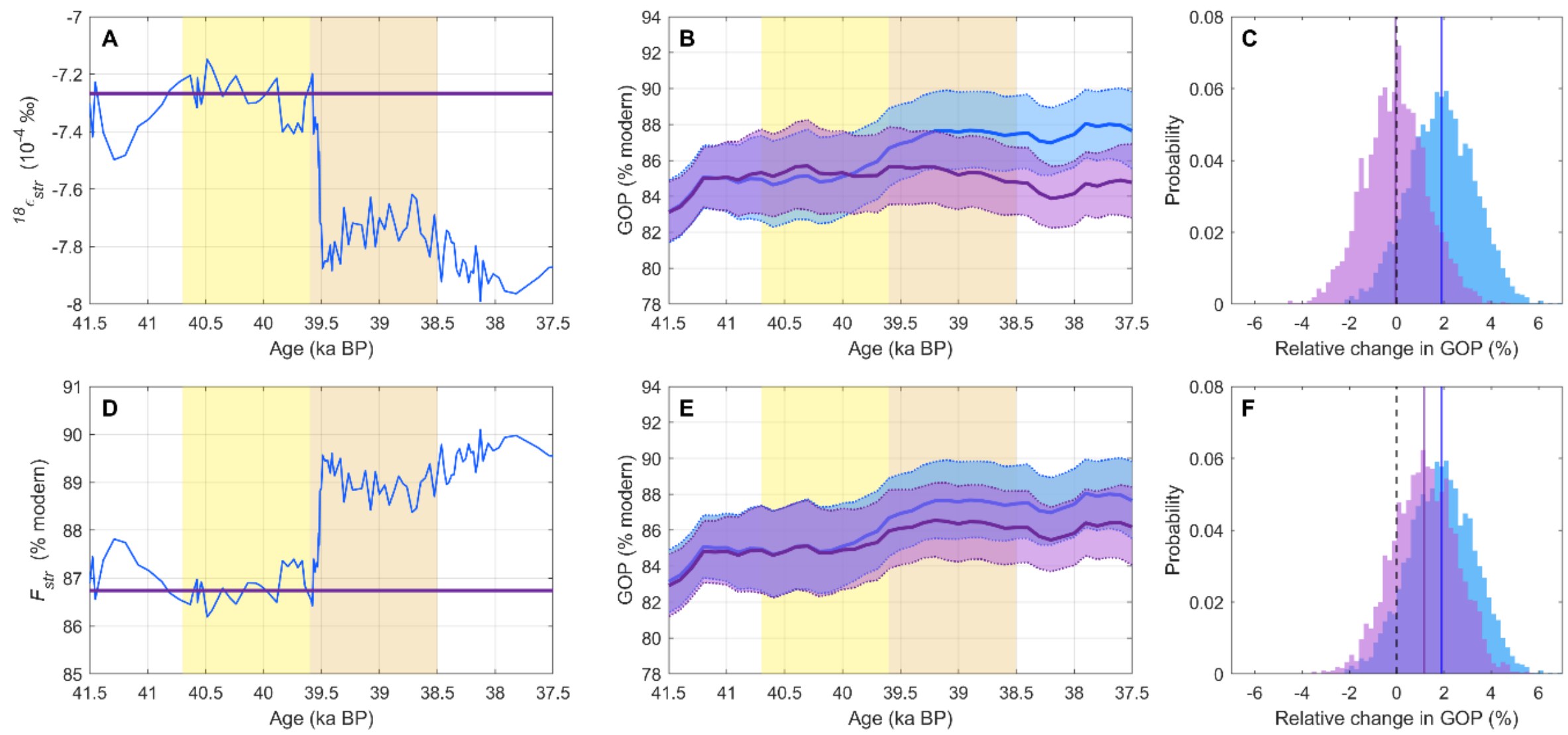

Fig. S3. Sensitivity tests of gross oxygen productivity (GOP) reconstruction for stratospheric processes. (**A** and **D**) The prescribed isotope fractionation effect in the stratosphere ($^{18}\varepsilon_{str}$) and the stratospheric influx to the troposphere (F_{str}). Blue curves indicate the default scenario and purple curves represent corresponding sensitivity experiment scenarios. (**B** and **E**) Comparison of resulting GOP. (**C** and **F**) Histogram of relative change of GOP between Period II and Period I. Yellow and orange shadings indicate Period I and Period II, respectively.

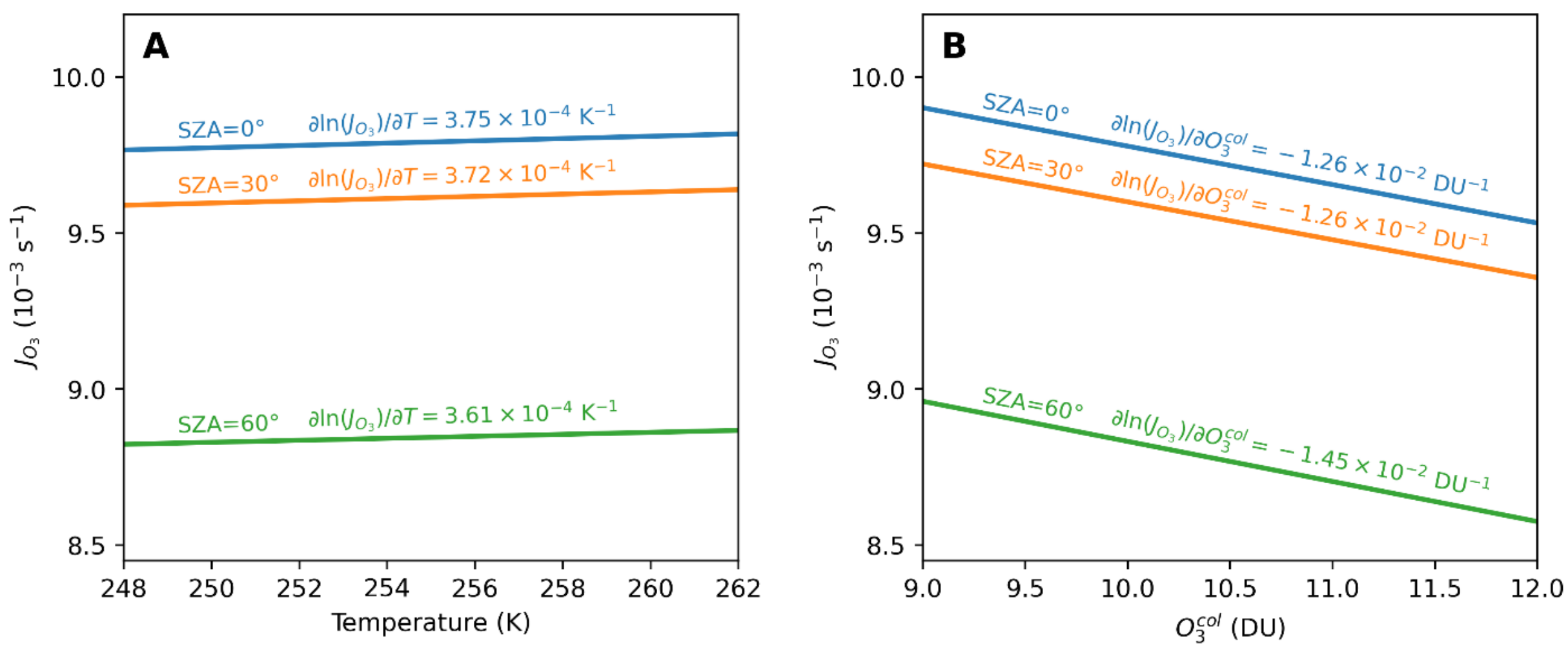

Fig. S4. Sensitivity of the ozone photolysis rate constant (J_{O_3}) to temperature and overhead ozone column. (**A**) Temperature dependence of J_{O_3} over 248 to 262 K at fixed overhead ozone column of 10 DU. (**B**) Overhead-ozone dependence of J_{O_3} over 9 to 12 DU at constant temperature of 250 K. Photolysis rates are computed with the TUV model for solar zenith angle of 0°, 30° and 60°. Annotations report the logarithmic sensitivities of J_{O_3} to temperature and overhead ozone column.

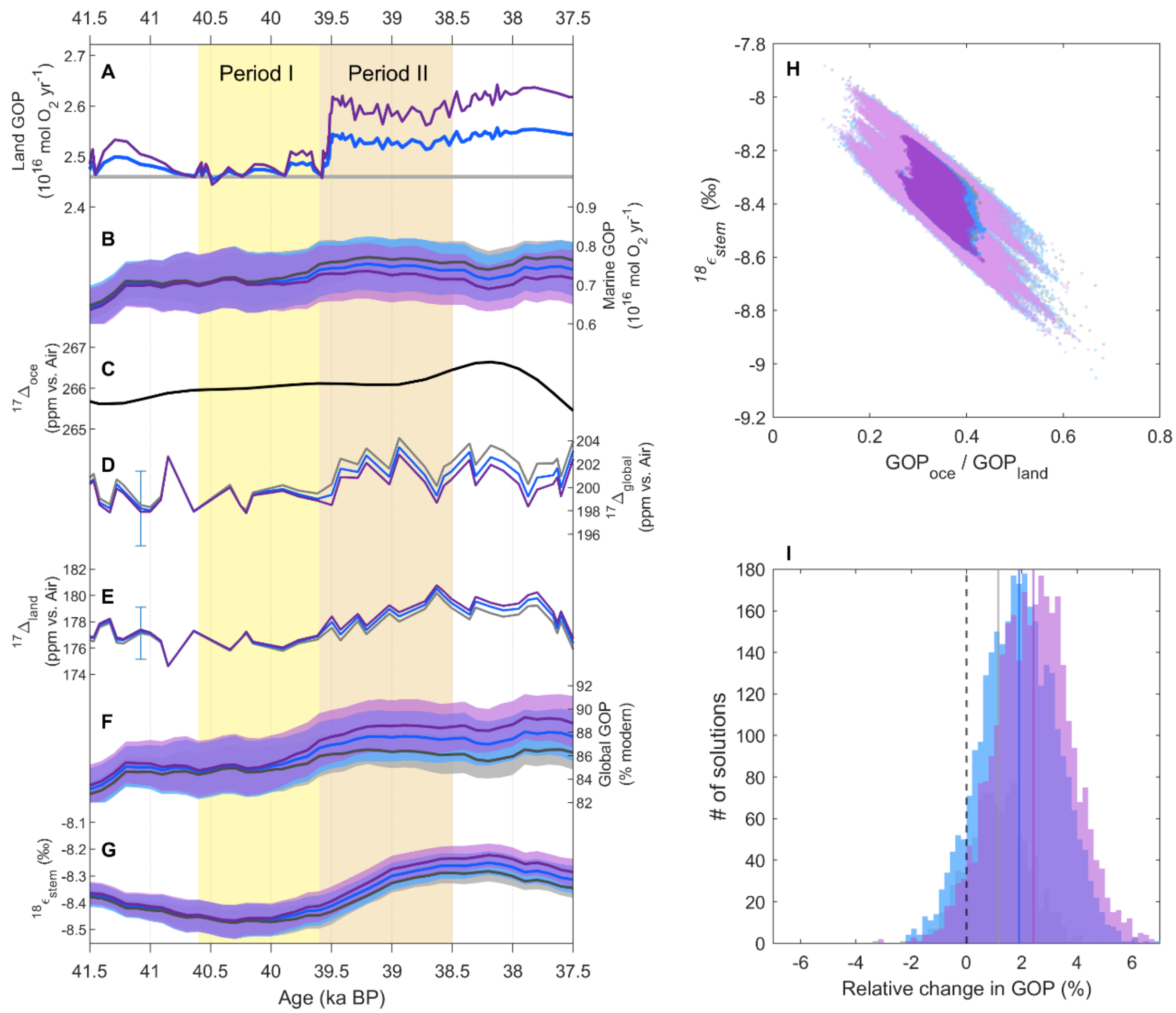

Fig. S5. Sensitivity tests of gross oxygen productivity (GOP) reconstruction for prescribed land productivity. (**A**) Different scenarios of prescribed land GOP. Blue curve is the default scenario, purple one represents the double-sensitivity case, and grey is constant land GOP scenario. (**B**) Calculated marine GOP in each scenario. (**C**) The $^{17}\Delta$ signature of marine photosynthetic O_2 ($^{17}\Delta_{oce}$). (**D**) The $^{17}\Delta$ signature of global photosynthetic O_2 ($^{17}\Delta_{global}$). (**E**) The $^{17}\Delta$ signature of terrestrial photosynthetic O_2 ($^{17}\Delta_{land}$). (**F**) Calculated global GOP and (**G**) isotopic composition of precipitation water. (**H**) The scatter plot of the ratio of oceanic- to terrestrial GOP to isotopic composition of precipitation water. (**I**) Comparison of relative change of GOP between Period II and Period I.

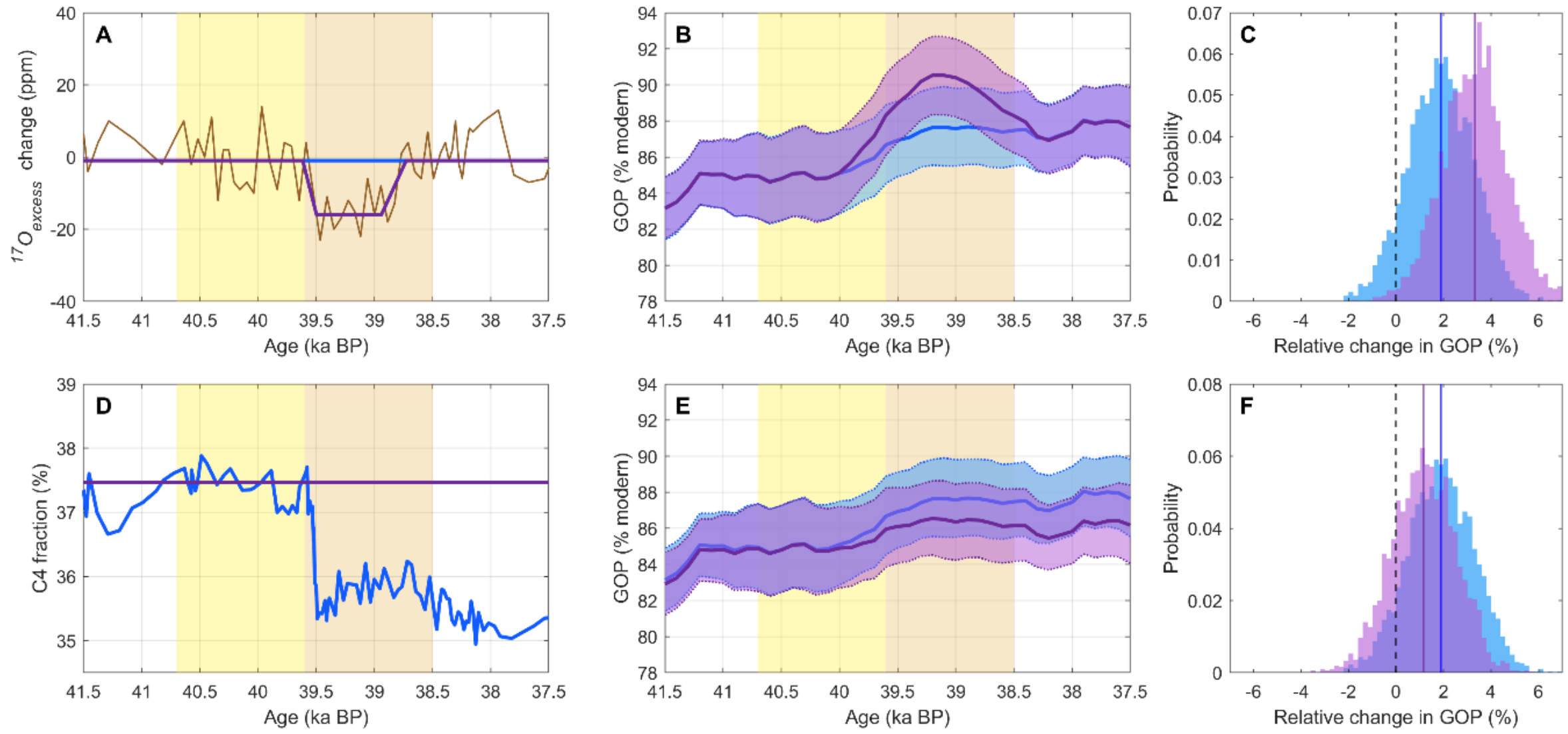

Fig. S6. Sensitivity tests of gross oxygen productivity (GOP) reconstruction for ^{17}O anomaly of precipitation water and fraction of C4 photosynthesis. (**A** and **D**) The prescribed ^{17}O anomaly ($^{17}O_{excess}$) and C4 photosynthesis fraction. Prescribed $^{17}O_{excess}$ are plotted as anomaly from the mean value over Period I. Blue curves indicate the default scenario and purple curves represent corresponding sensitivity experiment scenarios. Also plotted is the NEEM $^{17}O_{excess}$ record (*44*) expressed as anomaly from the mean value over the Period I. (**B** and **E**) Comparison of resulting GOP. (**C** and **F**) Histogram of relative change of GOP between Period II and Period I. Yellow and orange shadings indicate Period I and Period II, respectively.

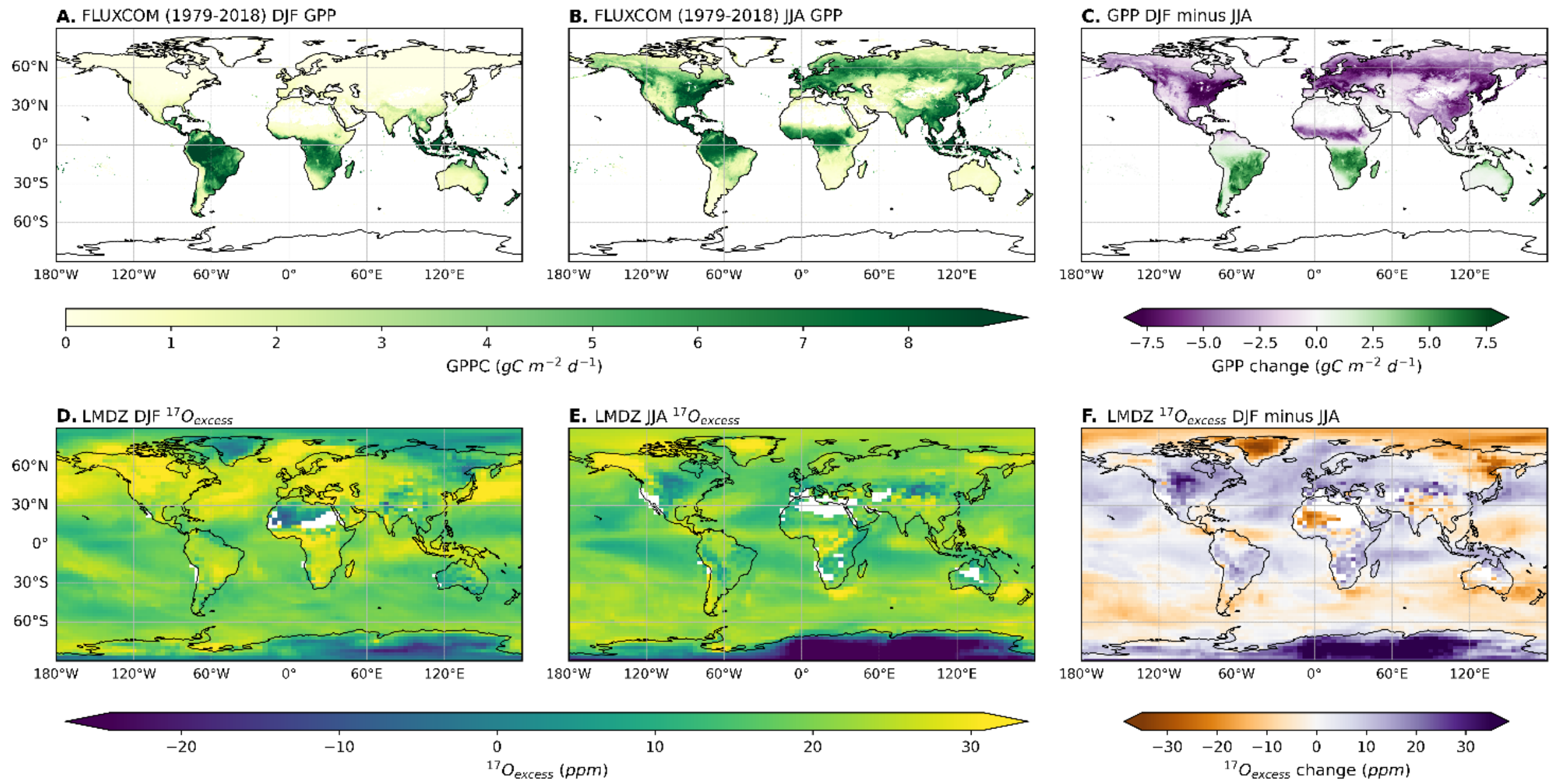

Fig. S7. Seasonal contrasts in terrestrial gross primary productivity (GPP) and precipitation ^{17}O$_{excess}$. (**A** and **B**) FLUXCOM GPP (1979 – 2018) for December-February (DJF) and June-August (JJA). (**C**) GPP change between DJF and JJA months. (**D** and **E**) Global precipitation ^{17}O$_{excess}$ simulated by LMDZ-iso for DJF and JJA. (**F**) ^{17}O$_{excess}$ change between DJF and JJA months.

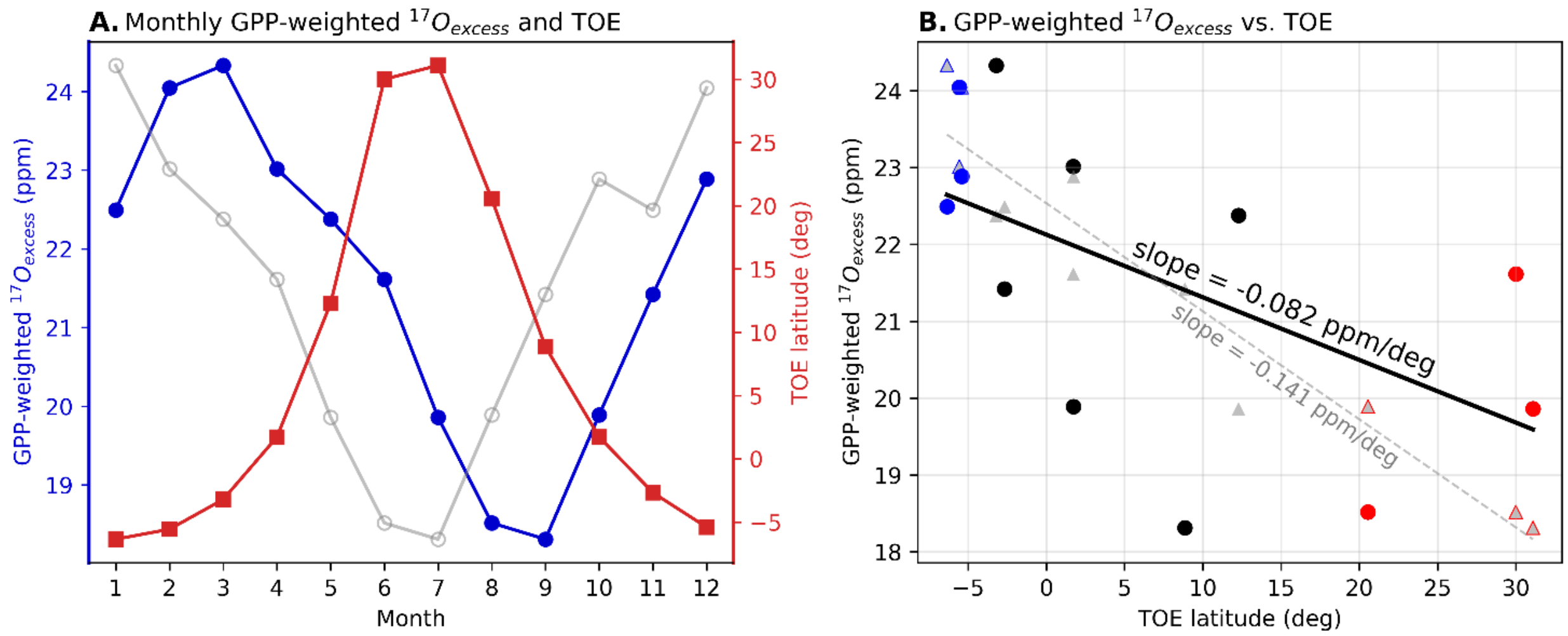

Fig. S8. Monthly variations of gross primary productivity (GPP)-weighted $^{17}O_{excess}$ and terrestrial oxygenesis equator (TOE). (**A**) Monthly climatology of GPP-weighted $^{17}O_{excess}$ (blue) and TOE latitude (red). The GPP-weighted $^{17}O_{excess}$ climatology shifted by 2 months is shown in grey. (**B**) Relationship between GPP-weighted $^{17}O_{excess}$ and TOE latitudes (solid circles) with corresponding linear regression (solid black line). Grey symbols and the dashed grey line show the relationship using the GPP-weighted $^{17}O_{excess}$ shifted forward by 2 months. Points corresponding to December-January-February and June-July-August are shown in blue and red, respectively.

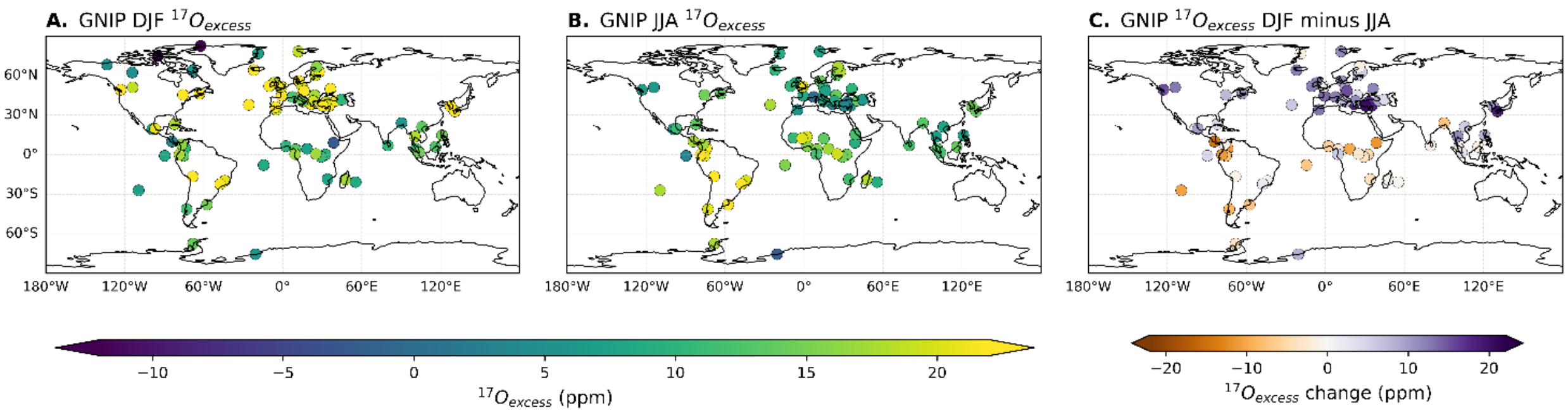

Fig. S9. Seasonal contrasts in precipitation $^{17}O_{excess}$ from the Global Network of Isotopes in Precipitation (GNIP) database. (**A** and **B**) GNIP $^{17}O_{excess}$ for December-February (DJF) and June-August (JJA). (**C**) $^{17}O_{excess}$ change between DJF and JJA months.

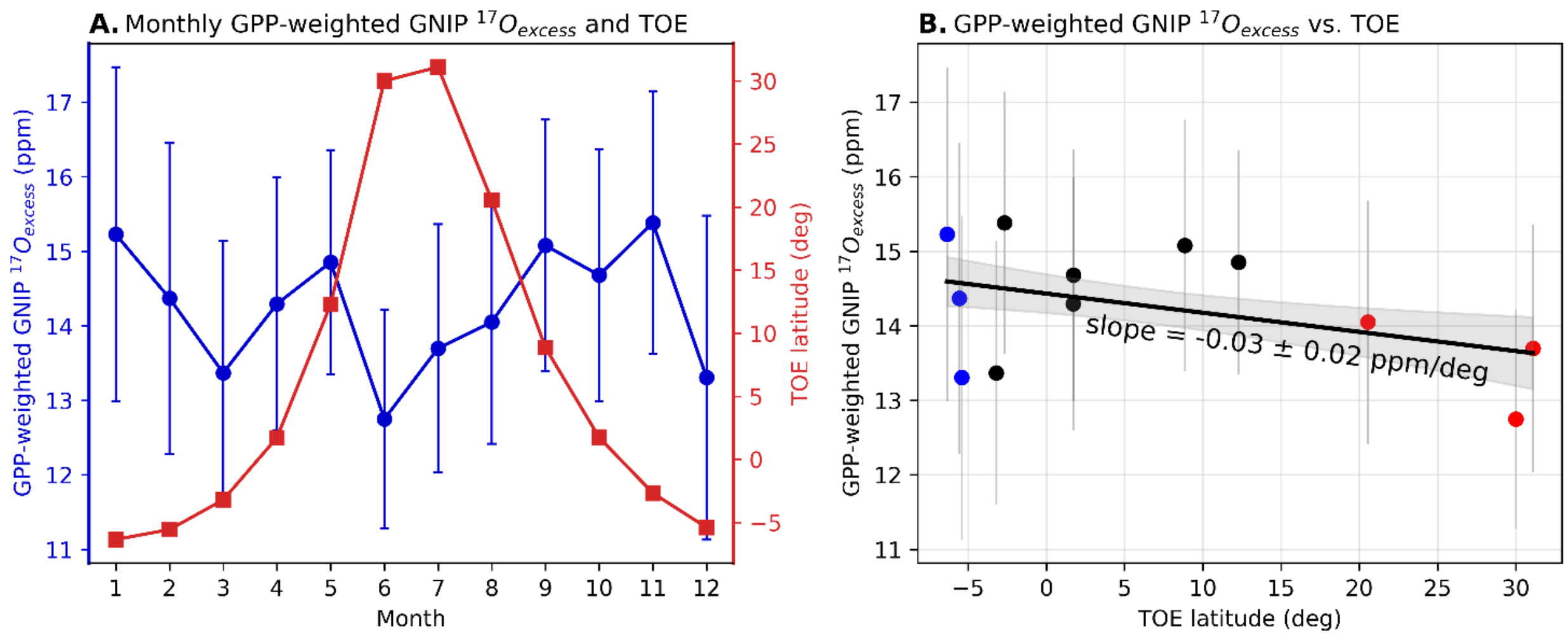

Fig. S10. Monthly variations of gross primary productivity (GPP)-weighted Global Network of Isotopes in Precipitation (GNIP) $^{17}O_{excess}$ and terrestrial oxygenesis equator (TOE). (**A**) Monthly climatology of GPP-weighted $^{17}O_{excess}$ from GNIP (blue) and TOE latitude (red). Error bars show the 1-sigma standard error of the GPP-weighted mean of $^{17}O_{excess}$. (**B**) Relationship between GPP-weighted $^{17}O_{excess}$ and TOE latitudes (solid circles) with corresponding linear regression (solid black line). Grey shading represents the 1-sigma envelope of the linear regression. Points corresponding to December-January-February and June-July-August are shown in blue and red, respectively.

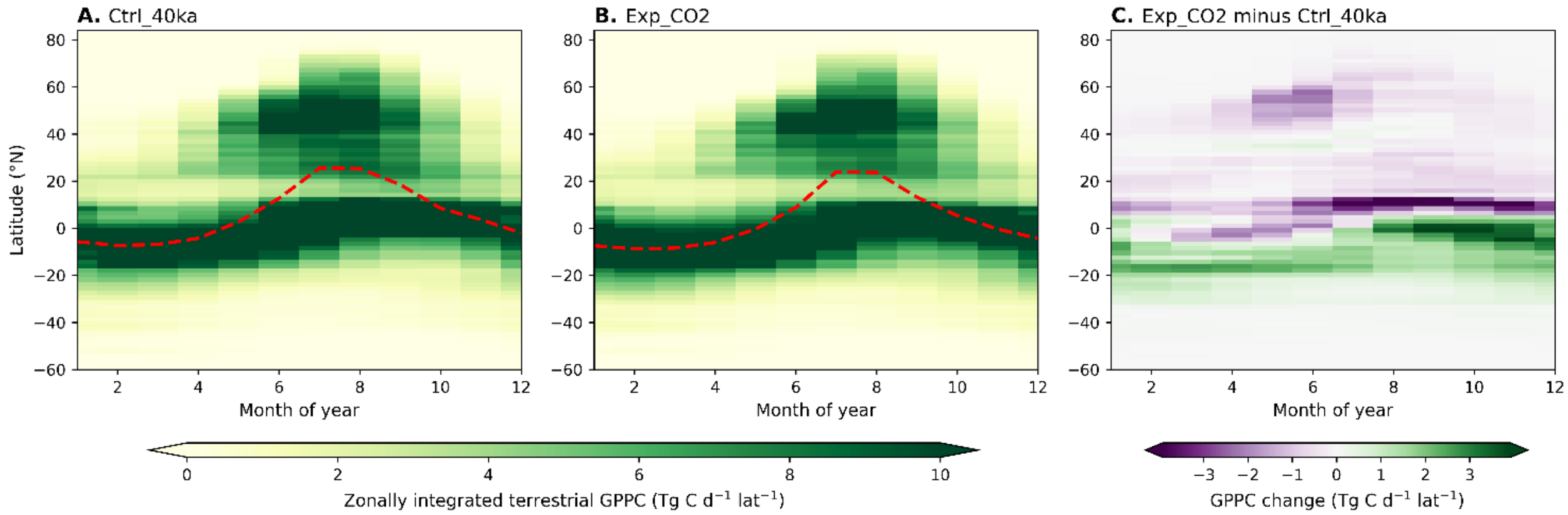

Fig. S11. Monthly climatology of zonally integrated terrestrial gross primary productivity (GPP) and terrestrial oxygenesis equator (TOE) latitudes in the IPSL-CM5A2-VLR simulations. (**A**) 200-year mean seasonal cycle of zonally integrated GPP (shading) and TOE latitudes (red dashed line) in Ctrl_40ka. (**B**) Same for (A) for Exp_CO2. (C) Difference in zonally integrated GPP between Exp_CO2 and Ctrl_40ka.

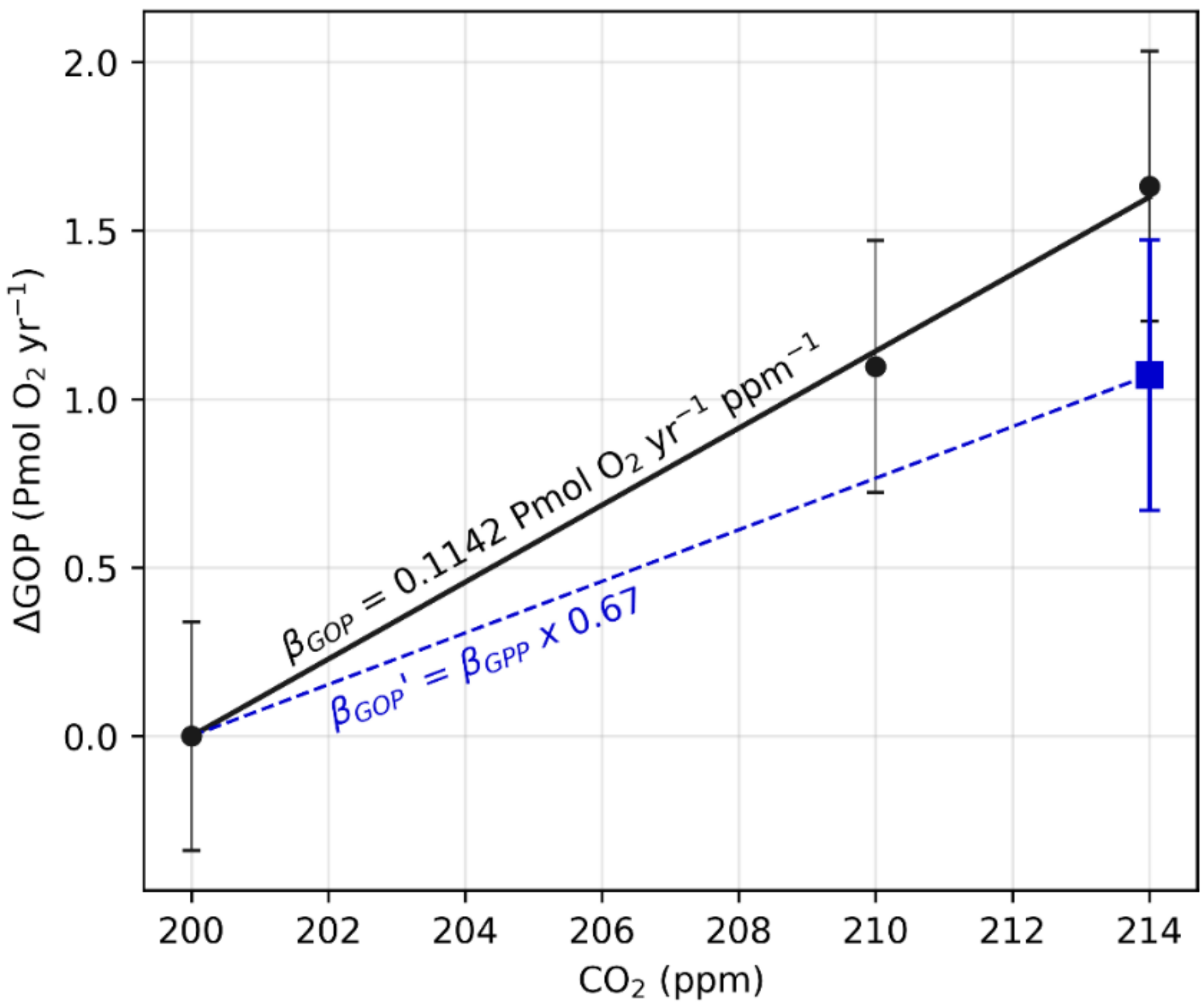

Fig. S12. Response of global terrestrial gross oxygen productivity (GOP) to prescribed CO_2 concentration. ΔGOP is the anomaly in global terrestrial GOP relative to that in Exp_DUST. β_{GOP} is estimated from a linear regression of ΔGOP in Exp_DUST, Exp_CO2, and Exp_214 (black circles and black solid line). A reduced sensitivity, β_{GOP}', is shown by blue dashed line. The blue square indicates the predicted ΔGOP for a 14-ppm CO_2 increase relative to Exp_DUST under the reduced sensitivity.

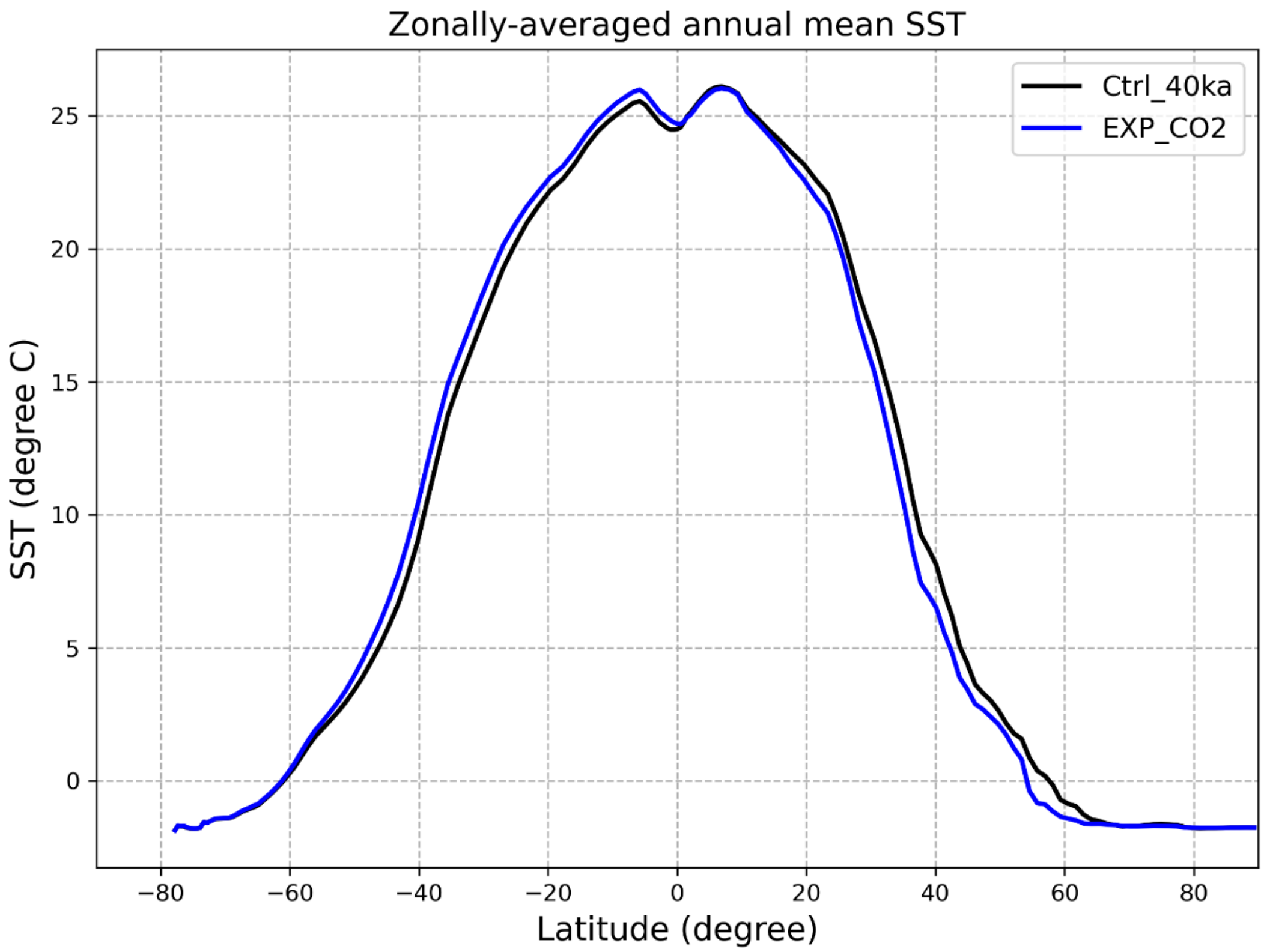

Fig. S13. Latitudinal profiles of zonally-averaged annual mean sea-surface temperature (SST) obtained from Ctrl_40ka (black) and Exp_CO2 (blue) experiments using IPSL-CM5A2-VLR model.

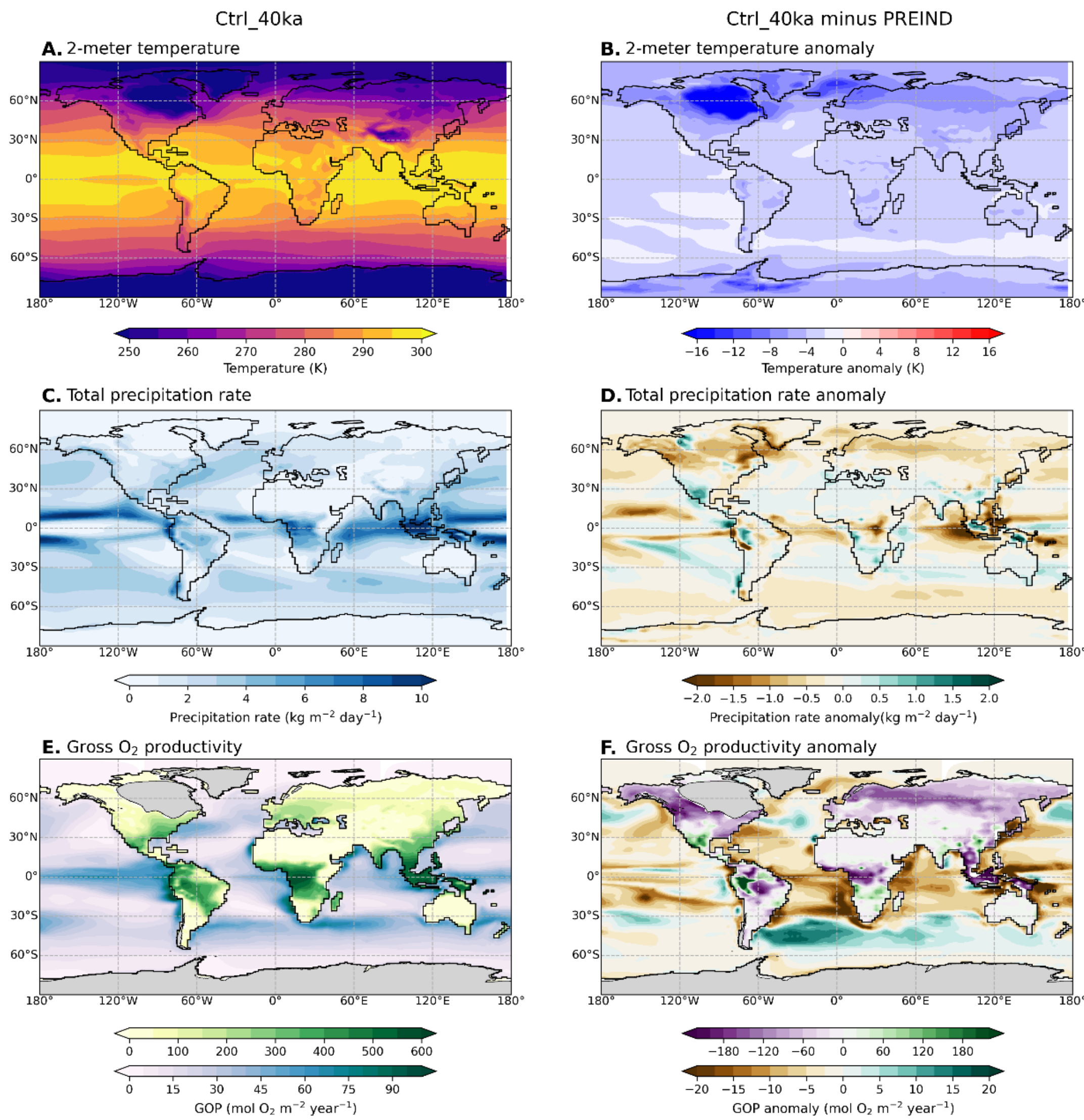

Fig. S14. Simulated climate in our control experiment (Ctrl_40ka) and comparison with pre-industrial experiment (PREIND). (A and B) 2-meter temperature, (**C** and **D**) total precipitation rate, and (**E** and **F**) terrestrial- and oceanic gross O_2 productivity (GOP).

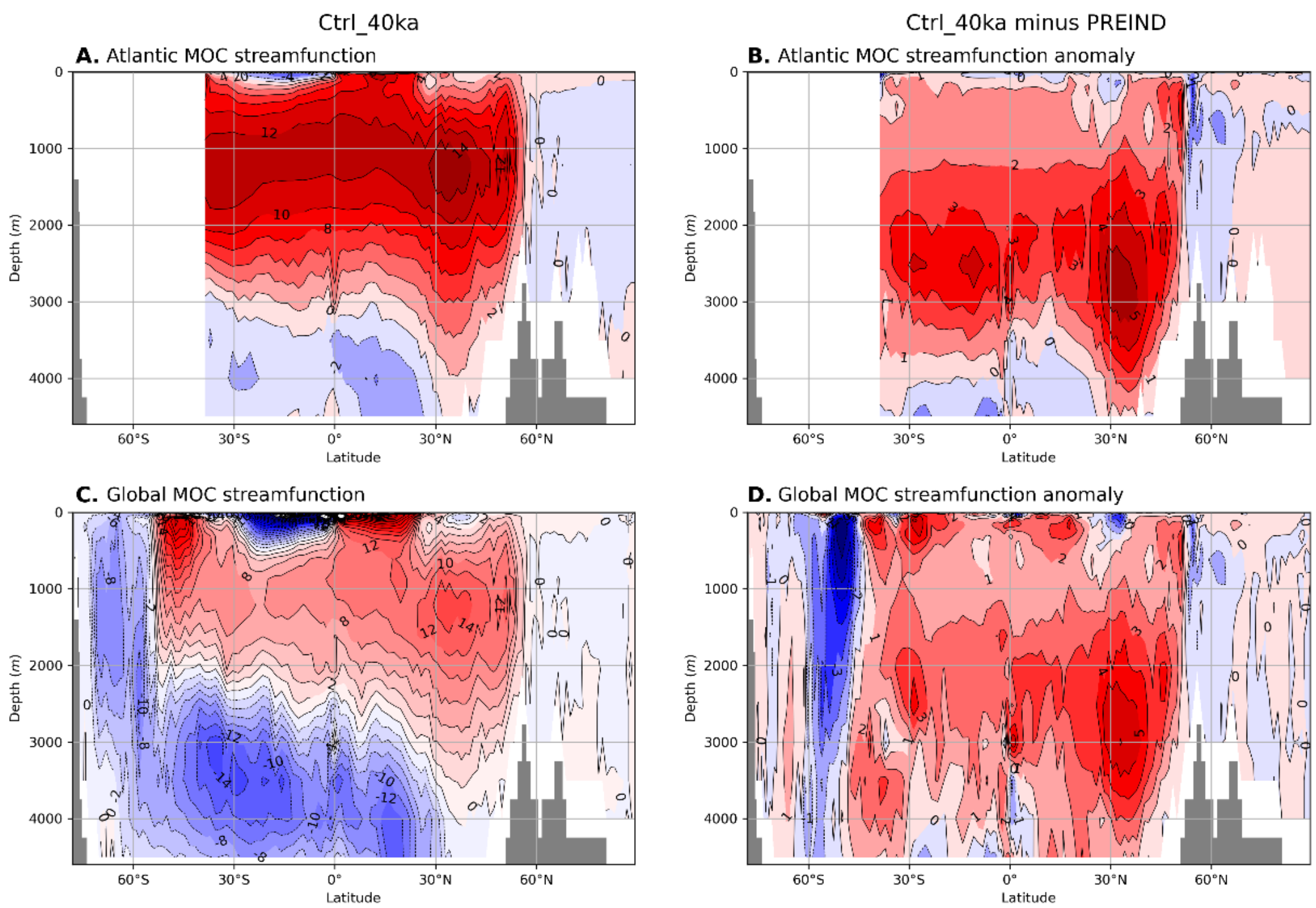

Fig. S15. Simulated meridional overturning circulation in our control experiment (Ctrl_40ka) and comparison with pre-industrial experiment (PREIND). (**A** and **B**) stream function of Atlantic MOC, and (**C** and **D**) stream function of global MOC.

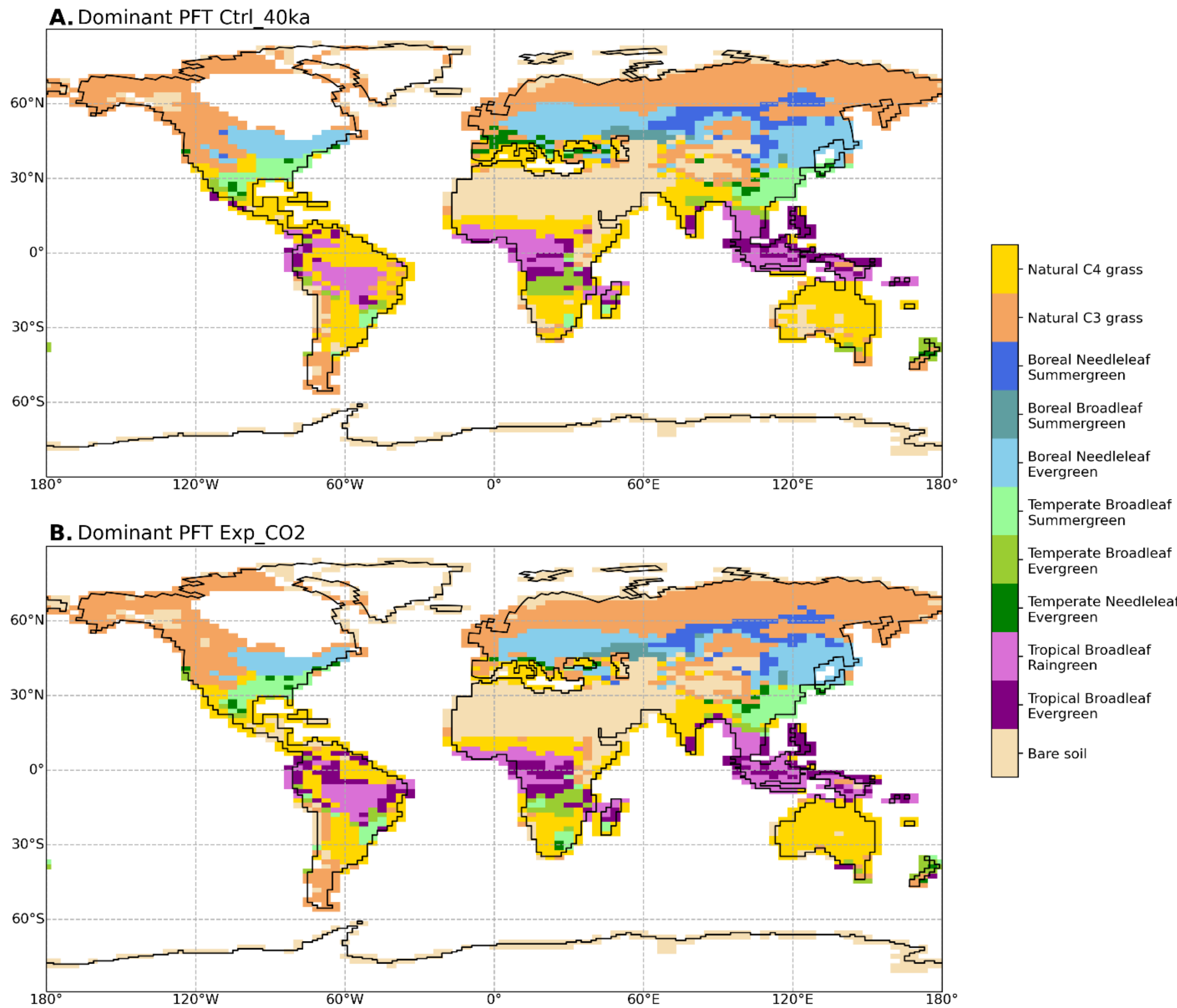

Fig. S16. Comparison of dominant plant functional types (PFTs) simulated in Ctrl_40ka (upper) and Exp_CO2 experiments (lower).

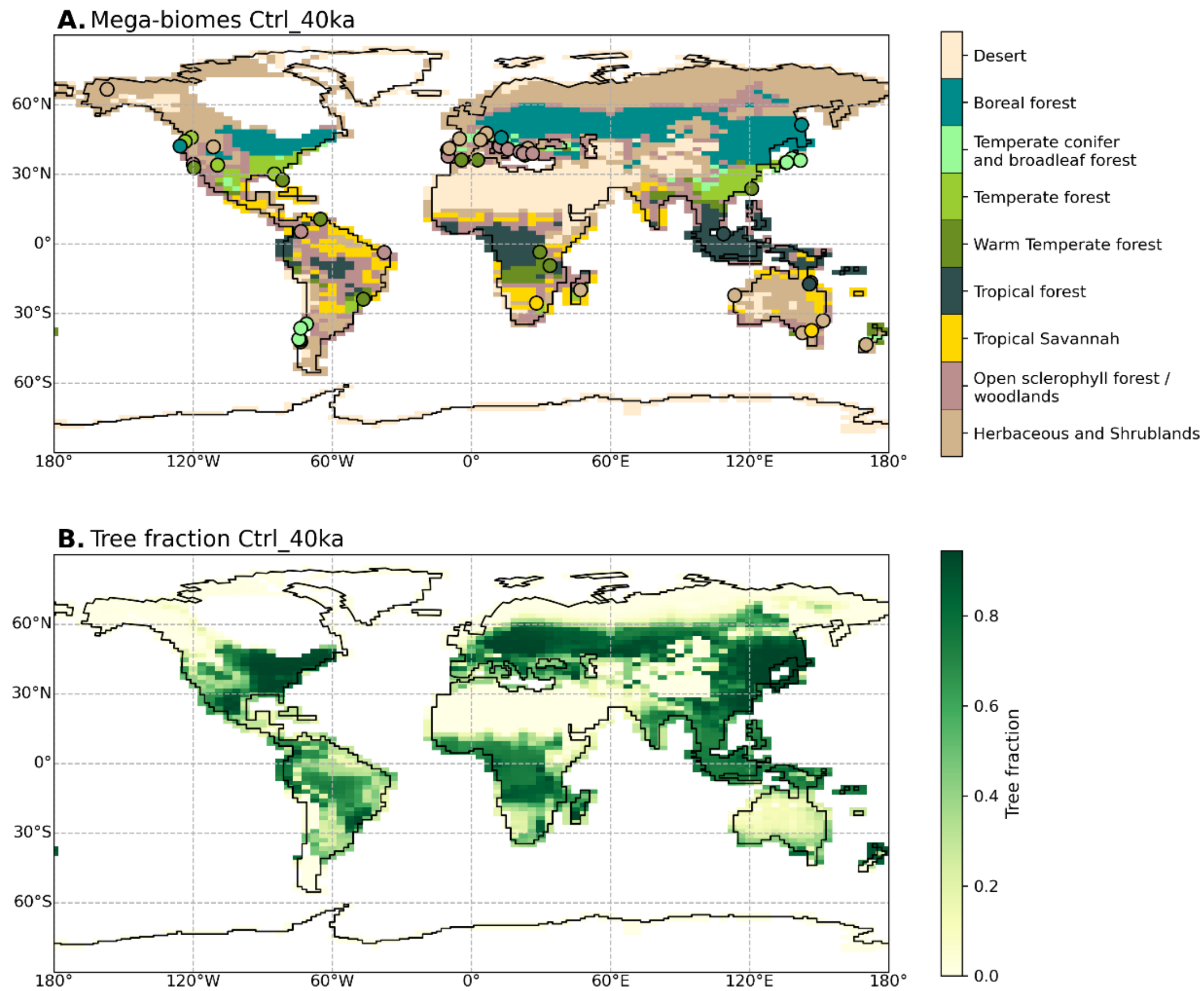

Fig. S17. Simulated mega-biomes (upper) and tree fraction (lower) in Ctrl_40ka experiment. Mega-biome distribution is obtained by transforming simulated plant functional types (PFTs) using criteria detailed in Table S1.

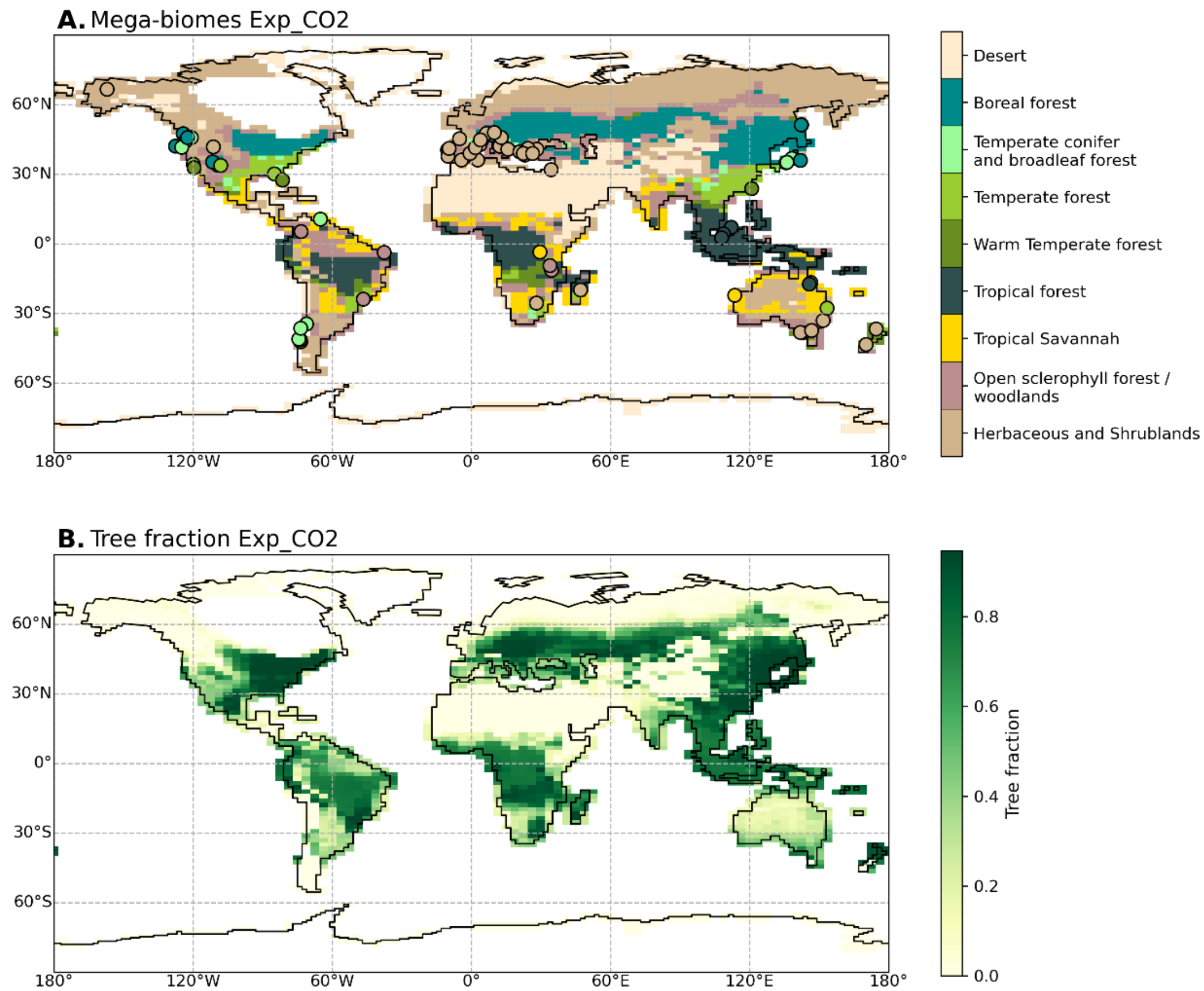

Fig. S18. Simulated mega-biomes (upper) and tree fraction (lower) in Exp_CO2 experiment. Mega-biome distribution is obtained by transforming simulated plant functional types (PFTs) using criteria detailed in Table S1.

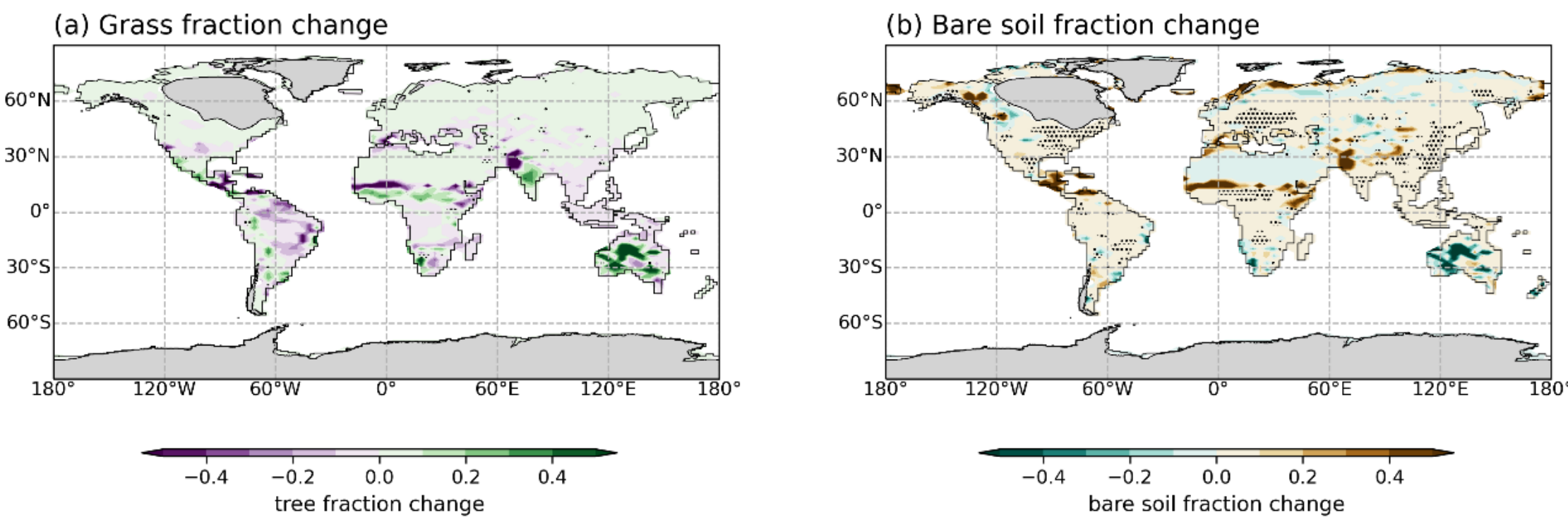

Fig. S19. Changes in simulated (a) grass fraction and (b) bare soil fraction between Exp_CO2 and Ctrl_40ka experiments.

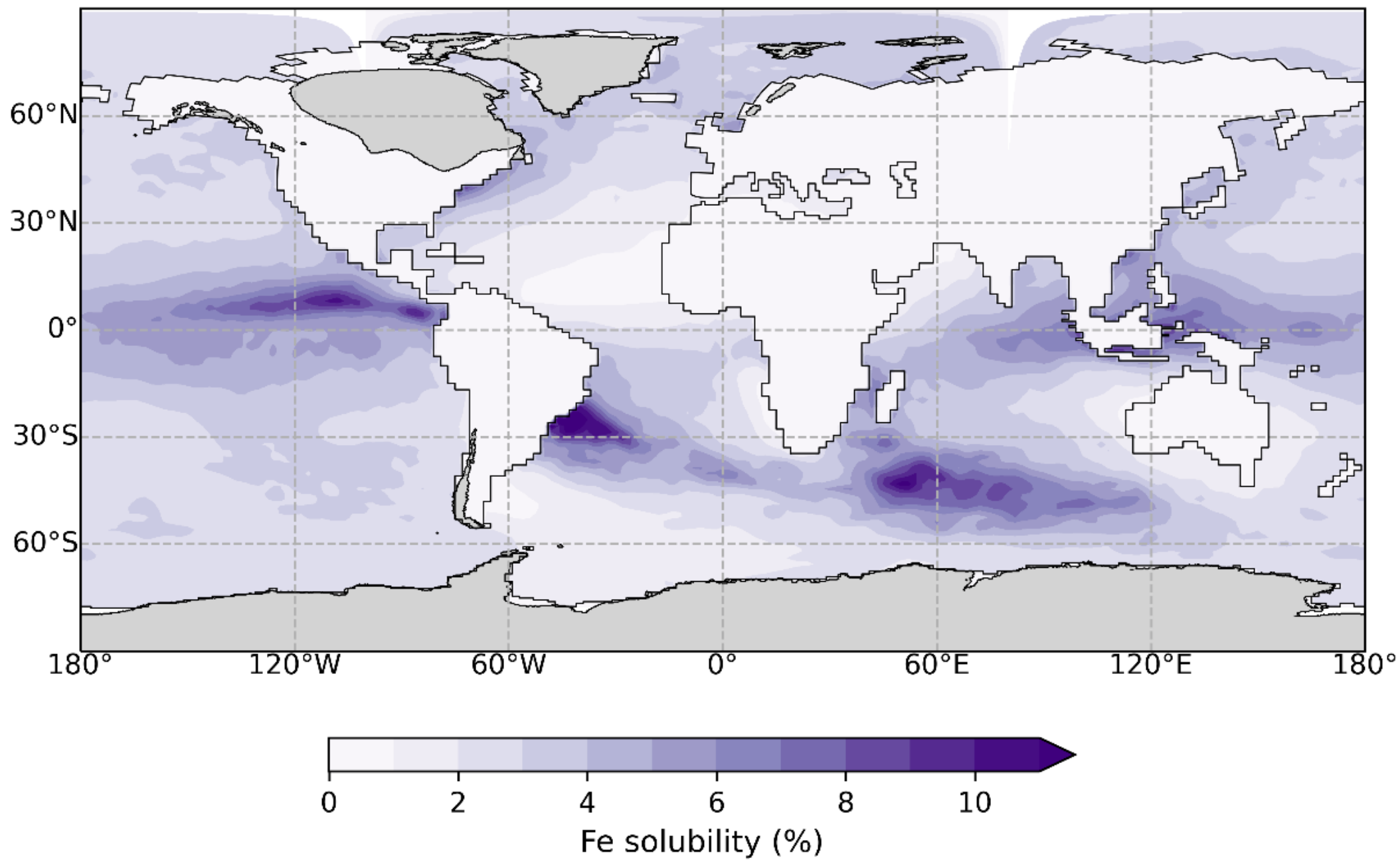

Fig. S20. Surface ocean Fe solubility field (*98*) prescribed in IPSL-CM5A2 simulations.

Table S1. List of mega-biomes and criteria of conversion of plant functional types (PFTs) to mega-biomes.

**Mega-biome**	**Criteria**
Herbaceous and Shrublands	Tree fraction < 20%<br>& Dominant PFT = Natural C3 grass or Natural C4 grass
	20% < Tree fraction < 40%<br>& Dominant PFT = Natural C3 grass or Natural C4 grass or bare soil<br>& Temperate or boreal region
Tropical savannah	20% < Tree fraction < 40%<br>& Dominant PFT = Natural C3 grass or Natural C4 grass or bare soil<br>& Tropical region
Open sclerophyll forest or woodlands	40% < Tree fraction < 70%
Tropical forest	Tree fraction > 70%<br>& Dominant PFT = Tropical Broadleaf Evergreen Trees or Tropical Broadleaf Raingreen Trees or Natural C4 grass (for CTRL) or Natural C3 grass (for FWF)<br>& Tropical region
Warm temperate forest	Tree fraction > 70%<br>& Dominant PFT = Tropical Broadleaf Evergreen Trees or Tropical Broadleaf Raingreen Trees or Natural C3 grass or Natural C4 grass<br>& Temperate region
	Tree fraction > 70%<br>& Dominant PFT = Temperate Broadleaf Evergreen Trees
Temperate forest	Tree fraction > 70%<br>& Temperate Broadleaf Summergreen Trees > 70%
Temperate conifer and Broadleaf forest	Tree fraction > 70%<br>& Temperate Needleleaf Evergreen Trees > 20%
Boreal forest	Tree fraction > 70%<br>& Dominant PFT = Boreal Needleleaf Evergreen Trees or Boreal Broadleaf Summergreen Trees or Boreal Needleleaf Summergreen Trees
Desert	Tree fraction < 20%<br>& Dominant PFT = Bare soil

Table S2. Global biosphere productivity obtained in this study (in Pmol O_2 year^{-1}). The GOP values inferred from ice-core data are obtained by multiplying the terrestrial and marine GOP ratios from the box model by their corresponding preindustrial values simulated by IPSL-CM5A2-VLR experiments. Relative changes in land, ocean, and global biosphere productivity with respect to Ctrl_40ka are given in parentheses.

		Gross O_2 productivity (Pmol O_2 year^{-1})				
		IPSL-CM5A2-VLR model			Ice-core data	
Experiment	Forcing	Land	Ocean	Global	Interval	Global
Ctrl_40ka	40ka	20.8 ± 0.4	8.8 ± 0.1	29.6 ± 0.4	Period I	29.7 ± 0.3
Exp_FWF	FWF	19.9 ± 0.3 (96 ± 2%)	8.5 ± 0.1 (96 ± 2%)	28.4 ± 0.4 (96 ± 2%)	-	-
Exp_DUST	FWF+dust	19.9± 0.3 (96 ± 2%)	8.6 ± 0.1 (97 ± 2%)	28.6 ± 0.4 (96 ± 2%)	-	-
Exp_CO2	FWF+dust+CO_2	21.0 ± 0.4 (101 ± 3%)	8.7 ± 0.1 (98 ± 2%)	29.7 ± 0.4 (100 ± 2%)	Period II	30.4 ± 0.2 (102 ± 1%)
Exp_VFIX	FWF+dust+CO_2 +fixed vegetation	21.3 ± 0.3 (103 ± 2%)	8.7 ± 0.1 (99 ± 2%)	30.1 ± 0.3 (102 ± 2%)	-	-

Data S1.

The summary of $^{17}\Delta$ measurements of air O_2 trapped in the NEEM ice-core samples. The $^{17}\Delta$ values are reported relative to the modern air standard collected in Saclay, France, whose isotopic ratios are determined by regular measurements. Bag numbers correspond to the 55-cm-long sections of the ice core. Sample depths indicate the midpoint depths of the samples taken from each bag and analyzed in this study.

Bag number	Sample depth	$^{17}\Delta$	1-sigma
	(meter)	(ppm vs. air)	(ppm)
NEEM 3166	1741.18	20	6
NEEM 3168	1741.98	27	6
NEEM 3169	1742.53	34	2
NEEM 3174	1745.28	36	2
NEEM 3176	1746.35	37	9
NEEM 3178	1747.68	18	9
NEEM 3181	1749.23	33	6
NEEM Gas-S 3187 B 51 INT	1752.60	27	6
NEEM Gas-S 3188 B 51	1753.15	26	4
NEEM Gas 3189 B INT	1753.70	41	2
NEEM Gas-S 3192 B INT	1755.33	23	4
NEEM 3193	1755.85	31	9
NEEM Gas-S 3194 B 51 INT	1756.45	26	11
NEEM 3198	1758.63	19	10
NEEM Gas-S 3200 C 17	1759.92	15	9
NEEM 3203	1761.45	24	0
NEEM Gas-S 3208 B 51 INT	1764.15	30	0
NEEM Gas 3212 B INT	1766.37	31	5
NEEM Gas-S 3216 C 21	1768.72	23	9
NEEM 3218	1769.49	35	6
NEEM 3221	1771.13	27	10
NEEM 3222	1771.68	22	15
NEEM 3223	1772.23	17	6
NEEM 3224	1773.08	27	11
NEEM 3228	1774.97	39	0
NEEM 3229	1775.83	28	0
NEEM 3232 GAS	1777.61	38	1
NEEM 3234	1778.28	31	1
NEEM 3236 C	1779.70	32	8
NEEM 3238	1780.48	20	11
NEEM 3240	1781.58	30	6
NEEM Gas-S 3242 B 51	1782.85	32	10
NEEM Gas-K 3244 C 21	1784.12	36	9
NEEM 3248 B	1786.19	33	2
NEEM 3249	1786.68	26	3
NEEM Gas-S 3250	1787.27	30	10
NEEM 3252	1788.18	37	9
NEEM 3255	1789.98	27	3

NEEM Gas-S 3257	1791.27	50	9
NEEM Gas-S 3258 B INT	1791.65	32	5
NEEM Gas-S 3259 B 47	1792.22	26	5
NEEM 3260	1792.73	26	6
NEEM 3263	1794.23	33	1
NEEM 3264 GAS	1795.10	34	6
NEEM 3266	1796.11	22	6
NEEM Gas-S 3269 B 25	1797.87	26	4
NEEM 3271	1798.93	37	4
NEEM 3273	1800.03	34	15
NEEM 3275	1800.98	25	7
NEEM 3277	1801.93	37	9
NEEM 3278	1802.48	21	5
NEEM 3279	1803.03	14	4
NEEM 3281	1804.28	18	3
NEEM 3282	1804.79	44	2

REFERENCES

1. P. G. Falkowski, J. A. Raven, *Aquatic Photosynthesis* (Princeton Univ. Press, 2013).

2. T. F. Keenan, X. Luo, B. D. Stocker, M. G. De Kauwe, B. E. Medlyn, I. C. Prentice, N. G. Smith, C. Terrer, H. Wang, Y. Zhang, S. Zhou, A constraint on historic growth in global photosynthesis due to rising CO_2. *Nat. Clim. Change* **13**, 1376–1381 (2023).

3. A. U. Igamberdiev, P. J. Lea, Land plants equilibrate O_2 and CO_2 concentrations in the atmosphere. *Photosynth. Res.* **87**, 177–194 (2006).

4. H. D. Holland, The oxygenation of the atmosphere and oceans. *Philos. Trans. R. Soc. B Biol. Sci.* **361**, 903–915 (2006).

5. C. B. Field, M. J. Behrenfeld, J. T. Randerson, P. Falkowski, Primary production of the biosphere: Integrating terrestrial and oceanic components. *Science* **281**, 237–240 (1998).

6. C. Beer, M. Reichstein, E. Tomelleri, P. Ciais, M. Jung, N. Carvalhais, C. Rödenbeck, M. A. Arain, D. Baldocchi, G. B. Bonan, A. Bondeau, A. Cescatti, G. Lasslop, A. Lindroth, M. Lomas, S. Luyssaert, H. Margolis, K. W. Oleson, O. Roupsard, E. Veenendaal, N. Viovy, C. Williams, F. I. Woodward, D. Papale, Terrestrial gross carbon dioxide uptake: Global distribution and covariation with climate. *Science* **329**, 834–838 (2010).

7. A. Anav, P. Friedlingstein, C. Beer, P. Ciais, A. Harper, C. Jones, G. Murray-Tortarolo, D. Papale, N. C. Parazoo, P. Peylin, S. Piao, S. Sitch, N. Viovy, A. Wiltshire, M. Zhao, Spatiotemporal patterns of terrestrial gross primary production: A review. *Rev. Geophys.* **53**, 785–818 (2015).

8. S. Rahmstorf, J. E. Box, G. Feulner, M. E. Mann, A. Robinson, S. Rutherford, E. J. Schaffernicht, Exceptional twentieth-century slowdown in Atlantic Ocean overturning circulation. *Nat. Clim. Change* **5**, 475–480 (2015).

9. P. Ditlevsen, S. Ditlevsen, Warning of a forthcoming collapse of the Atlantic meridional overturning circulation. *Nat. Commun.* **14**, 4254 (2023).

10. M. Kageyama, U. Merkel, B. Otto-Bliesner, M. Prange, A. Abe-Ouchi, G. Lohmann, R. Ohgaito, D. M. Roche, J. Singarayer, D. Swingedouw, X. Zhang, Climatic impacts of fresh water hosing under Last Glacial Maximum conditions: A multi-model study. *Clim. Past* **9**, 935–953 (2013).

11. L. C. Jackson, R. Kahana, T. Graham, M. A. Ringer, T. Woollings, J. V. Mecking, R. A. Wood, Global and European climate impacts of a slowdown of the AMOC in a high resolution GCM. *Climate Dynam.* **45**, 3299–3316 (2015).

12. W. Liu, A. V. Fedorov, S.-P. Xie, S. Hu, Climate impacts of a weakened Atlantic Meridional Overturning Circulation in a warming climate. *Sci. Adv.* **6**, eaaz4876 (2020).

13. H. Saini, G. Pontes, J. R. Brown, R. N. Drysdale, Y. Du, L. Menviel, Australasian hydroclimate response to the collapse of the atlantic meridional overturning circulation under pre-industrial and last interglacial climates. *Paleoceanogr. Paleoclimatol.* **40**, e2024PA004967 (2025).

14. B. Fox-Kemper, H. T. Hewitt, C. Xiao, G. Aðalgeirsdóttir, S. S. Drijfhout, T. L. Edwards, N. R. Golledge, M. Hemer, R. E. Kopp, G. Krinner, A. Mix, D. Notz, S. Nowicki, I. S. Nurhati, L. Ruiz, J.-B. Sallée, A. B. A. Slangen, Y. Yu, Ocean, cryosphere, and sea level change, in *Climate Change 2021: The Physical Science Basis. Contribution of Working Group I to the Sixth Assessment Report of the Intergovernmental Panel on Climate Change* (Cambridge Univ. Press, 2021), chap. 9.

15. R. Liebermann, M. Hofmann, G. Feulner, Shutdown of Atlantic overturning circulation could cause persistent increase of primary production in the Pacific. *Environ. Res. Lett.* **19**, 024005 (2024).

16. L. G. Henry, J. F. McManus, W. B. Curry, N. L. Roberts, A. M. Piotrowski, L. D. Keigwin, North Atlantic ocean circulation and abrupt climate change during the last glaciation. *Science* **353**, 470–474 (2016).

17. J. Lynch-Stieglitz, The Atlantic Meridional Overturning Circulation and abrupt climate change. *Ann. Rev. Mar. Sci.* **8**, 83–104 (2017).

18. W. Dansgaard, S. J. Johnsen, H. B. Clausen, D. Dahl-Jensen, N. S. Gunderstrup, C. U. Hammer, C. S. Hvidberg, J. P. Steffensen, A. E. Sveinbjörnsdottir, J. Jouzel, G. Bond, Evidence for general instability of past climate from a 250-kyr ice-core record. *Nature* **364**, 218–220 (1993).

19. T. Blunier, E. J. Brook, Timing of millennial-scale climate change in Antarctica and Greenland during the last glacial period. *Science* **291**, 109–112 (2001).

20. WAIS Divide Project Members, Precise interpolar phasing of abrupt climate change during the last ice age. *Nature* **520**, 661–665 (2015).

21. T. F. Stocker, S. J. Johnsen, A minimum thermodynamic model for the bipolar seesaw. *Paleoceanography* **18**, 1087 (2003).

22. J. B. Pedro, M. Jochum, C. Buizert, F. He, S. Barker, S. O. Rasmussen, Beyond the bipolar seesaw: Toward a process understanding of interhemispheric coupling. *Quat. Sci. Rev.* **192**, 27–46 (2018).

23. S. Barker, P. Diz, M. J. Vautravers, J. Pike, G. Knorr, I. R. Hall, W. S. Broecker, Interhemispheric Atlantic seesaw response during the last deglaciation. *Nature* **457**, 1097–1102 (2009).

24. J. Ahn, E. J. Brook, Siple Dome ice reveals two modes of millennial CO_2 change during the last ice age. *Nat. Commun.* **5**, 3723 (2014).

25. T. K. Bauska, S. A. Marcott, E. J. Brook, Abrupt changes in the global carbon cycle during the last glacial period. *Nat. Geosci.* **14**, 91–96 (2021).

26. M. Jochum, Z. Chase, R. Nuterman, J. Pedro, S. Rasmussen, G. Vettoretti, P. Zheng, Carbon fluxes during Dansgaard-Oeschger events as simulated by an Earth system model. *J. Climate* **35**, 5745–5758 (2022).

27. H. Saini, K. J. Meissner, L. Menviel, K. Kvale, Transient response of Southern Ocean ecosystems during Heinrich stadials. *Paleoceanogr. Paleoclimatol.* **39**, e2023PA004754 (2024).

28. B. Riddell-Young, J. E. Lee, E. J. Brook, J. Schmitt, H. Fischer, T. K. Bauska, J. A. Menking, R. Iseli, J. R. Clark, Abrupt changes in biomass burning during the last glacial period. *Nature* **637**, 91–96 (2025).

29. K. A. Wendt, E. J. Brook, J. A. Higgins, J. E. Lee, J. A. Menking, T. S. Bauska, V. Gkinis, J. P. Severinghaus, Southern Ocean drives multidecadal atmospheric CO_2 rise during Heinrich stadials. *Proc. Natl. Acad. Sci. U.S.A.* **121**, e2319652121 (2024).

30. V. Mariotti, L. Bopp, A. Tagliabue, M. Kageyama, D. Swingedouw, Marine productivity response to Heinrich events: A model-data comparison. *Clim. Past* **8**, 1581–1598 (2012).

31. L. Menviel, A. Timmermann, A. Mouchet, O. Timm, Meridional reorganizations of marine and terrestrial productivity during Heinrich events. *Paleoceanography* **23**, PA1203 (2008).

32. C. Reutenauer, A. Landais, T. Blunier, C. Breant, M. Kageyama, M.-N. Woillez, C. Risi, V. Mariotti, P. Braconnot, Quantifying molecular oxygen isotope variations during a Heinrich stadial. *Clim. Past* **11**, 1527–1551 (2015).

33. B. Luz, E. Barkan, M. L. Bender, M. H. Thiemens, K. A. Boering, Triple-isotope composition of atmospheric oxygen as a tracer of biosphere productivity. *Nature* **400**, 547–550 (1999).

34. T. Blunier, B. Barnett, M. L. Bender, M. B. Hendricks, Biological oxygen productivity during the last 60,000 years from triple oxygen isotope measurements. *Global Biogeochem. Cycles* **16**, 1029 (2002).

35. M. F. Miller, Isotopic fractionation and the quantification of ^{17}O anomalies in the oxygen three-isotope system: An appraisal and geochemical significance. *Geochim. Cosmochim. Acta* **66**, 1881–1889 (2002).

36. B. Luz, E. Barkan, The isotopic ratios $^{17}O/^{16}O$ and $^{18}O/^{16}O$ in molecular oxygen and their significance in biogeochemistry. *Geochim. Cosmochim. Acta* **69**, 1099–1110 (2005).

37. X. Yin, P. C. Struik, C3 and C4 photosynthesis models: An overview from the perspective of crop modelling. *NJAS Wageningen J. Life Sci.* **57**, 27–38 (2009).

38. T. Blunier, M. L. Bender, B. Barnett, J. C. von Fischer, Planetary fertility during the past 400 ka based on the triple isotope composition of O_2 in trapped gases from the Vostok ice core. *Clim. Past* **8**, 1509–1526 (2012).

39. A. Landais, J. Lathiere, E. Barkan, B. Luz, Reconsidering the change in global biosphere productivity between the Last Glacial Maximum and present day from the triple oxygen isotopic composition of air trapped in ice cores. *Global Biogeochem. Cycles* **21**, GB1025 (2007).

40. M. Brandon, A. Landais, S. Duchamp-Alphonse, V. Favre, L. Schmitz, H. Abrial, F. Prié, T. Extier, T. Blunier, Exceptionally high biosphere productivity at the beginning of Marine Isotopic Stage 11. *Nat. Commun.* **11**, 2112 (2020).

41. J.-W. Yang, M. Brandon, A. Landais, S. Duchamp-Alphonse, T. Blunier, F. Prié, T. Extier, Global biosphere primary productivity changes during the past eight glacial cycles. *Science* **375**, 1145–1151 (2022).

42. P. Sepulchre, A. Caubel, J.-B. Ladant, L. Bopp, O. Boucher, P. Braconnot, P. Brockmann, A. Cozic, Y. Donnadieu, J.-L. Dufresne, V. Estella-Pèrez, C. Ethé, F. Fluteau, M.-A. Foujols, G. Gastineau, J. Ghattas, D. Hauglustaine, F. Hourdin, M. Kageyama, M. Khodri, O. Marti, Y. Meurdesoif, J. Mignot, A.-C. Sarr, J. Servonnat, D. Swingedouw, S. Szopa, D. Tardif, IPSL-CM5A2 – An Earth system model designed for multi-millennial climate simulations. *Geosci. Model Dev.* **13**, 3011–3053 (2020).

43. M. L. Bender, T. Sowers, L. Labeyrie, The Dole effect and its variations during the last 130,000 years as measured in the Vostok ice core. *Global Biogeochem. Cycles* **8**, 363–376 (1994).

44. S. O. Rasmussen, P. M. Abbott, T. Blunier, A. J. Bourne, E. J. Brook, S. L. Buchardt, C. Buizert, J. Chappellaz, H. B. Clausen, E. Cook, D. Dahl-Jensen, S. M. Davies, M. Guillevic, S. Kopfstuhl, T. Laepple, I. K. Seierstad, J. P. Severinghaus, J. P. Steffensen, C. Stowasser, A. Svensson, P. Vallelonga, B. M. Vinther, F. Wilhelms, M. Winstrup, A first chronology for the North Greenland Eemian Ice Drilling (NEEM) ice core. *Clim. Past* **9**, 2713–2730 (2013).

45. J. Chappellaz, C. Stowasser, T. Blunier, D. Baslev-Clausen, E. J. Brook, R. Dallmayr, X. Faïn, J. E. Lee, L. E. Mitchell, O. Pascual, D. Romanini, J. Rosen, S. Schüpbach, High-resolution glacial and deglacial record of atmospheric methane by continuous-flow and laser spectrometer analysis along the NEEM ice core. *Clim. Past* **9**, 2579–2593 (2013).

46. R. H. Rhodes, E. J. Brook, J. C. H. Chiang, T. Blunier, O. J. Maselli, J. R. McConnell, D. Romanini, J. P. Severinghaus, Enhanced tropical methane production in response to iceberg discharge in the North Atlantic. *Science* **348**, 1016–1019 (2015).

47. T. K. Bauska, E. J. Brook, S. A. Marcott, D. Baggenstos, S. Shackleton, J. P. Severinghaus, V. V. Petrenko, Controls on millennial-scale atmospheric CO_2 variability during the Last Glacial Period. *Geophys. Res. Lett.* **45**, 7731–7740 (2018).

48. M. Guillevic, L. Bazin, A. Landais, C. Stowasser, V. Masson-Delmotte, T. Blunier, F. Eynaud, S. Falourd, E. Michel, B. Minster, T. Popp, F. Prié, B. M. Vinther, Evidence for a three-phase sequence during Heinrich stadial 4 using a multiproxy approach based on Greenland ice core records. *Clim. Past* **10**, 2115–2133 (2014).

49. J. P. Severinghaus, R. Beaudette, M. A. Headly, K. Taylor, E. J. Brook, Oxygen-18 of O_2 records the impact of abrupt climate change on the terrestrial biosphere. *Science* **324**, 1431–1434 (2009).

50. A. Seltzer, C. Buizert, D. Baggenstos, E. J. Brook, J. Ahn, J.-W. Yang, J. P. Severinghaus, Does $\delta^{18}O$ of O_2 record meridional shifts in tropical rainfall? *Clim. Past* **13**, 1323–1338 (2017).

51. Y. L. Yung, W. B. DeMore, J. P. Pinto, Isotopic exchange between carbon dioxide and ozone via $O(^1D)$ in the stratosphere. *Geophys. Res. Lett.* **18**, 13–16 (1991).

52. J. Wen, M. Thiemens, Multi-isotope study of the $O(^1D) + CO_2$ exchange and stratospheric consequences. *J. Geophys. Res.* **98**, 12753–12758 (1993).

53. E. L. Fleming, C. H. Jackman, R. S. Stolarski, A. R. Douglas, A model study of the impact of source gas changes on the stratosphere for 1850-2100. *Atmos. Chem. Phys.* **11**, 8515–8541 (2011).

54. E. D. Young, L. Y. Yeung, I. E. Kohl, On the $\Delta^{17}O$ budget of atmospheric O_2. *Geochim. Cosmochim. Acta* **135**, 102–125 (2014).

55. J. B. Burkholder, S. P. Sander, J. P. D. Abbatt, J. R. Barker, C. Cappa, J. D. Crounse, T. S. Dibble, R. E. Huie, C. E. Kolb, M. J. Kurylo, V. L. Orkin, C. J. Percival, D. M. Wilmouth, P. H. Wine, *Chemical Kinetics and Photochemical Data for Use in Atmospheric Studies*, Evaluation Number 19, JPL Publication 19–5 (Jet Propulsion Laboratory, 2020).

56. H. F. Goessling, S. Bathiany, Why CO_2 cools the middle atmosphere – A consolidating model perspective. *Earth Sys. Dyn.* **7**, 697–715 (2016).

57. N. Butchart, The Brewer-Dobson circulation. *Rev. Geophys.* **52**, 157–184 (2014).

58. L. Geng, L. T. Murray, L. J. Mickley, P. Lin, Q. Fu, A. J. Schauer, B. Alexander, Isotopic evidence of multiple controls on atmospheric oxidants over climate transitions. *Nature* **546**, 133–136 (2017).

59. S. Albani, N. M. Mahowald, L. N. Murphy, R. Raiswell, J. K. Moore, R. F. Anderson, D. McGee, L. I. Bradtmiller, B. Delmonte, P. P. Hesse, P. A. Mayewski, Paleodust variability since the Last Glacial Maximum and implications for iron inputs to the ocean. *Geophys. Res. Lett.* **43**, 3944–3954 (2016).

60. L. N. Murphy, A. C. Clement, S. Albani, N. M. Mahowald, P. Swart, M. M. Arienzo, Simulated changes in atmospheric dust in response to a Heinrich stadial. *Paleoceanography* **29**, 30–43 (2014).

61. A. J. Broccoli, K. A. Dahl, R. J. Stouffer, The response of the ITCZ to Northern Hemisphere cooling. *Geophys. Res. Lett.* **33**, L24703 (2006).

62. W. Chen, D. Zhu, P. Ciais, C. Huang, N. Viovy, M. Kageyama, Response of vegetation cover to CO_2 and climate changes between the Last Glacial Maximum and pre-industrial period in a dynamic global vegetation model. *Quat. Sci. Rev.* **218**, 293–305 (2019).

63. A. Schmittner, Decline of the marine ecosystem caused by a reduction in the Atlantic overturning circulation. *Nature* **434**, 628–633 (2005).

64. O. Aumont, C. Ethé, A. Tagliabue, L. Bopp, M. Gehlen, PISCES-v2: An ocean biogeochemical model for carbon and ecosystem studies. *Geosci. Model Dev.* **8**, 2465–2513 (2015).

65. S. P. Harrison, M. F. Sanchez-Goñi, Global patterns of vegetation response to millennial-scale variability and rapid climate change during the last glacial period. *Quat. Sci. Rev.* **29**, 2957–2980 (2010).

66. F. Pöppelmeier, A. Jeltsch-Thömmes, J. Lippold, F. Joos, T. F. Stocker, Multi-proxy constraints on Atlantic circulation dynamics since the last ice age. *Nat. Geosci.* **16**, 349–356 (2023).

67. D. S. Goll, M. Bauters, H. Zhang, P. Ciais, Y. Balkanski, R. Wang, H. Verbeeck, Atmospheric phosphorus deposition amplifies carbon sinks in simulations of a tropical forest in Central Africa. *New Phytol.* **237**, 2054–2068 (2022).

68. R. Wang, D. Goll, Y. Balkanski, D. Hauglustaine, O. Boucher, P. Ciais, I. Janssens, J. Penuelas, B. Guenet, J. Sardans, L. Bopp, N. Vuichard, F. Zhou, B. Li, S. Piao, S. Peng, Y. Huang, S. Tao, Global forest carbon update due to nitrogen and phosphorus deposition from 1850 to 2100. *Glob. Change Biol.* **23**, 4854–4872 (2017).

69. M. Hein, K. Sand-Jensen, CO_2 increases oceanic primary production. *Nature* **388**, 526–527 (1997).

70. P. Schippers, M. Lürling, M. Scheffer, Increase of atmospheric CO_2 promotes phytoplankton productivity. *Ecol. Lett.* **7**, 446–451 (2004).

71. E. M. Shoenfelt, G. Winckler, F. Lamy, R. F. Anderson, B. C. Bostick, Highly bioavailable dust-borne iron delivered to the Southern Ocean during glacial periods. *Proc. Natl. Acad. Sci. U.S.A.* **115**, 11180–11185 (2018).

72. H. Saini, K. J. Meissner, L. Menviel, K. Kvale, Impact of iron fertilisation on atmospheric CO_2 during the last glaciation. *Clim. Past* **19**, 1559–1584 (2023).

73. M. Wang, Q. Fu, S. Solomon, R. H. White, B. Alexander, Stratospheric ozone in the Last Glacial Maximum. *J. Geophys. Res.* **125**, e2020JD032929 (2020).

74. K. A. Wendt, A. D. Häuselmann, D. Fleitmann, A. E. Berry, X. Wang, A. S. Auler, H. Cheng, R. Lawrence Edwards, Three-phased Heinrich Stadial 4 recorded in NE Brazil stalagmites. *Earth Planet. Sci. Lett.* **510**, 94–102 (2019).

75. E. Barkan, B. Luz, High precision measurements of $^{17}O/^{16}O$ and $^{18}O/^{16}O$ ratios in H_2O. *Rapid Commun. Mass Spectrom.* **19**, 3737–3742 (2005).

76. C. Buizert, K. M. Cuffey, J. P. Severinghaus, D. Baggenstos, T. J. Fudge, E. J. Steig, B. R. Markle, M. Winstrup, R. H. Rhodes, E. J. Brook, T. A. Sowers, G. D. Clow, H. Cheng, R. L. Edwards, M. Sigl, J. R. McConnell, K. C. Taylor, The WAIS Divide deep ice core WD2014 chronology – Part 1: Methane synchronization (68-31 ka B.P.) and the gas age-ice age difference. *Clim. Past* **11**, 153–173 (2015).

77. H. Craig, L. I. Gordon, Deuterium and oxygen-18 variations in the ocean and the marine atmosphere. in *Stable Isotopes in Oceanographic Studies and Paleotemperatures*, E. Tongiorgi, Ed. (Laboratorio di Geologia Nucleare, 1965), pp. 9–130.

78. B. Luz, E. Barkan, Proper estimation of marine gross O_2 production with $^{17}O/^{16}O$ and $^{18}O/^{16}O$ ratios of dissolved O_2. *Geophys. Res. Lett.* **38**, L19606 (2011).

79. F. Joos, S. Gerber, I. C. Prentice, B. L. Otto-Bliesner, P. J. Valdes, Transient simulations of Holocene atmospheric carbon dioxide and terrestrial carbon since the Last Glacial Maximum. *Global Biogeochem. Cycles* **18**, e2003GB002156 (2004).

80. S. von Caemmerer, *Biochemical Models of Leaf Photosynthesis* (CSIRO Publishing, 2000).

81. K. M. Grant, E. J. Rohling, M. Bar-Matthews, A. Ayalon, M. Medina-Elizalde, C. Bronk Ramsey, C. Satow, A. P. Roberts, Rapid coupling between ice volume and polar temperature over the past 150,000 years. *Nature* **491**, 744–747 (2012).

82. F. Hourdin, M.-A. Foujols, F. Codron, V. Guemas, J.-L. Dufresne, S. Bony, S. Denvil, L. Guez, F. Lott, J. Ghattas, P. Braconnot, O. Marti, Y. Meurdesoif, L. Bopp, Impact of the LMDZ atmospheric grid configuration on the climate and sensitivity of the IPSL-CM5A coupled model. *Climate Dynam.* **40**, 2167–2192 (2013).

83. G. Krinner, N. Viovy, N. de Noblet-Ducoudré, J. Ogée, J. Polcher, P. Friedlingstein, P. Ciais, S. Sitch, I. C. Prentice, A dynamic global vegetation model for studies of the coupled atmosphere-biosphere system. *Global Biogeochem. Cycles* **19**, (2005).

84. G. Madec, R. Bourdallé-Badie, P.-A. Bouttier, C. Bricaud, D. Bruciaferri, D. Calvert, D., J. Chanut, E. Clementi, A. Coward, D. Delrosso, C. Ethé, S. Flavoni, T. Graham, J. Harle, D. Iovino, D. Lea, C. Lévy, T. Lovato, N. Martin, S. Masson, S. Mocavero, J. Paul, C. Rousset, D. Storkey, A. Storto, M. Vancoppenolle, NEMO ocean engine (Version v3.6), in Notes Du Pôle De Modélisation De L'institut Pierre-simon Laplace (IPSL), Zenodo, https://doi.org/10.5281/zenodo.3248739.

85. T. Fichefet, M. A. Morales Maqueda, Sensitivity of a global sea ice model to the treatment of ice thermodynamics and dynamics. *J. Geophys. Res.* **102**, 12609–12646 (1997).

86. S. Valcke, The OASIS3 coupler: A European climate modelling community software. *Geosci. Model Dev.* **6**, 373–388 (2013).

87. Y. Meurdesoif, A. Caubel, R. Lacroix, J. Dérouillat, M. H. Nguyen, XIOS tutorial, Zenodo, https://doi.org/10.5281/zenodo.20560336.

88. G. Madec, M. Imbard, A global ocean mesh to overcome the North Pole singularity. *Climate Dynam.* **12**, 381–388 (1996).

89. J.-L. Dufresne, M.-A. Foujols, S. Denvil, A. Caubel, O. Marti, O. Aumont, Y. Balkanski, S. Bekki, H. Bellenger, R. Benshila, S. Bony, L. Bopp, P. Braconnot, P. Brockmann, P. Cadule,

F. Cheruy, F. Codron, A. Cozic, D. Cugnet, N. de Noblet, J.-P. Duvel, C. Ethé, L. Fairhead, T. Fichefet, S. Flavoni, P. Friedlingstein, J.-Y. Grandpeix, L. Guez, E. Guilyardi, D. Hauglustaine, F. Hourdin, A. Idelkadi, J. Ghattas, S. Joussaume, M. Kageyama, G. Krinner, S. Labetoulle, A. Lahellec, M.-P. Lefebre, F. Lefevre, C. Lévy, Z. X. Li, J. Lloyd, F. Lott, G. Madec, M. Mancip, M. Marchand, S. Masson, Y. Meurdesoif, J. Mignot, I. Musat, S. Parouty, J. Polcher, C. Rio, M. Schulz, D. Swingedouw, S. Szopa, C. Talandier, P. Terray, N. Viovy, N. Vuichard, Climate change projections using the IPSL-CM5 Earth System Model: From CMIP3 to CMIP5. *Climate Dynam.* **40**, 2123–2165 (2013).

90. B. S. Ferster, A. Simon, A. Fedorov, J. Mignot, E. Guilyardi, Slowdown and recovery of the Atlantic Meridional Overturning Circulation and a persistent North Atlantic Warming Hole induced by Arctic sea ice decline. *Geophys. Res. Lett.* **49**, e2022GL097967 (2022).

91. A.-C. Sarr, Y. Donnadieu, C. T. Bolton, J.-B. Ladant, A. Licht, F. Fluteau, M. Laugié, D. Tardif, G. Dupont-Nivet, Neogene South Asian monsoon rainfall and wind histories diverged due to topographic effects. *Nat. Geosci.* **15**, 314–319 (2022).

92. M. Kageyama, S. P. Harrison, M.-L. Kapsch, M. Lofverstrom, J. M. Lora, U. Mikolajewicz, S. Sherriff-Tadano, T. Vadsaria, A. Abe-Ouchi, N. Bouttes, D. Chandan, L. J. Gregoire, R. F. Ivanovic, K. Izumi, A. N. LeGrande, F. Lhardy, G. Lohmann, P. A. Morozova, R. Ohgaito, A. Paul, W. Richard Peltier, C. J. Poulsen, A. Quiquet, D. M. Roche, X. Shi, J. E. Tierney, P. J. Valdes, E. Volodin, J. Zhu, The PMIP4 Last Glacial Maximum experiments: Preliminary results and comparison with the PMIP3 simulations. *Clim. Past* **17**, 1065–1089 (2021).

93. E. J. Gowan, X. Zhang, S. Khosravi, A. Rovere, P. Stocchi, A. L. C. Hughes, R. Gyllencreutz, J. Mangerud, J.-I. Svendsen, G. Lohmann, A new global ice sheet reconstruction for the past 80000 years. *Nat. Commun.* **12**, 1199 (2021).

94. M.-N. Woillez, G. Levavasseur, A.-L. Daniau, M. Kageyama, D. H. Urrego, M.-F. Sánchez-Goñi, V. Hanquiez, Impact of precession on the climate, vegetation and fire activity in southern Africa during MIS4. *Clim. Past* **10**, 1165–1182 (2014).

95. P. Le Mézo, L. Beaufort, L. Bopp, P. Braconnot, M. Kageyama, From monsoon to marine productivity in the Arabian Sea: Insights from glacial and interglacial climates. *Clim. Past* **13**, 759–778 (2017).

96. J. K. Moore, S. C. Doney, J. A. Kleypas, D. M. Glover, I. Y. Fung, An intermediate complexity marine ecosystem model for the global domain. *Deep Sea Res.* **49**, 403–462 (2001).

97. N. M. Mahowald, T. D. Jickells, A. R. Baker, P. Artaxo, C. R. Benitez-Nelson, G. Bergametti, T. C. Bond, Y. Chen, D. D. Cohen, B. Herut, N. Kubilay, R. Losno, C. Luo, W. Maenhaut, K. A. McGee, G. S. Okin, R. L. Siefert, S. Tsukuda, Global distribution of atmospheric phosphorous sources, concentrations and deposition rates, and anthropogenic impacts. *Global Biogeochem. Cycles* **22**, e2008GB003240 (2008).

98. C. Luo, N. M. Mahowald, N. Meskhidze, Y. Chen, R. L. Siefert, A. R. Baker, A. M. Johansen, Estimation of iron solubility from observations and a global aerosol model. *J. Geophys. Res.* **110**, D23307 (2005).

99. L. Bopp, K. E. Kohfeld, C. Le Quéré, O. Aumont, Dust impact on marine biota and atmospheric CO_2 during glacial periods. *Paleoceanography* **18**, 1046 (2003).

100. V. Gkinis, S. B. Simonsen, S. L. Buchardt, J. W. C. White, B. M. Vinther, Water isotope diffusion rates from the NorthGRIP ice core for the last 16,000 years—Glaciological and paleoclimatic implications. *Earth Planet. Sci. Lett.* **405**, 132–141 (2014).

101. G. D. Farquhar, J. Lloyd, J. A. Taylor, L. B. Flanagan, J. P. Syvertsen, K. T. Hubick, S. C. Wong, J. R. Ehleringer, Vegetation effects on the isotope composition of oxygen in atmospheric CO_2. *Nature* **363**, 439–443 (1993).

102. W. J. Li, H. A. J. Meijer, The use of electrolysis for accurate $\delta^{17}O$ and $\delta^{18}O$ isotope measurements in water. *Isot. Environ. Health Stud.* **34**, 349–369 (1998).

103. D. Crisp, H. Dolman, T. Tanhua, G. A. McKinley, J. Hauck, A. Bastos, S. Sitch, S. Eggleston, V. Aich, How well do we understand the land-ocean-atmosphere carbon cycle? *Rev. Geophys.* **60**, e2021RG000736 (2022).

104. P. Ciais, Y. Yao, T. Gasser, A. Baccini, Y. Wang, R. Lauerwald, S. Peng, A. Bastos, W. Li, P. A. Raymond, J. G. Canadell, G. P. Peters, R. J. Andres, J. Chang, C. Yue, A. J. Dolman, V. Haverd, J. Hartmann, G. Laruelle, A. G. Konings, A. W. King, Y. Liu, S. Luyssaert, F. Maignan, P. K. Patra, A. Peregon, P. Regnier, J. Pongratz, B. Poulter, A. Shvidenko, R. Valentini, R. Wang, G. Broquet, Y. Yin, J. Zscheischler, B. Guenet, D. S. Goll, A.-P. Ballantyne, H. Yang, C. Qiu, D. Zhu, Empirical estimates of regional carbon budgets imply reduced global soil heterotrophic respiration. *Natl. Sci. Rev.* **8**, nwaa145 (2021).

105. P. Friedlingstein, M. W. Jones, M. O'Sullivan, R. M. Andrew, D. C. E. Bakker, J. Hauck, C. Le Quéré, G. P. Peters, W. Peters, J. Pongratz, S. Sitch, J. G. Canadell, P. Ciais, R. B. Jackson, S. R. Alin, P. Anthoni, N. R. Bates, M. Becker, N. Bellouin, L. Bopp, T. T. T. Chau, F. Chevallier, L. P. Chini, M. Cronin, K. I. Currie, B. Decharme, L. M. Djeutchouang, X. Dou, W. Evans, R. A. Feely, L. Feng, T. Gasser, D. Gilfillan, T. Gkritzalis, G. Grassi, L. Gregor, N. Gruber, O. Gürses, I. Harris, R. A. Houghton, G. C. Hurtt, Y. Iida, T. Ilyina, I. T. Luijkx, A. Jain, S. D. Jones, E. Kato, D. Kennedy, K. K. Goldewijk, J. Knauer, J. I. Korsbakken, A. Körtzinger, P. Landschützer, S. K. Lauvset, N. Lefèvre, S. Lienert, J. Liu, G. Marland, P. C. McGuire, J. R. Melton, D. R. Munro, J. E. M. S. Nabel, S.-I. Nakaoka, Y. Niwa, T. Ono, D. Pierrot, B. Poulter, G. Rehder, L. Resplandy, E. Robertson, C. Rödenbeck, T. M. Rosan, J. Schwinger, C. Schwingshackl, R. Séférian, A. J. Sutton, C. Sweeney, T. Tanhua, P. P. Tans, H. Tian, B. Tilbrook, F. Tubiello, G. R. van der Werf, N. Vuichard, C. Wada, R. Wanninkhof, A. J. Watson, D. Willis, A. J. Wiltshire, W. Yuan, C. Yue, X. Yue, S. Zaehle, J. Zeng, Global Carbon Budget 2021. *Earth Syst. Sci. Data* **14**, 1917–2005 (2022).

106. A. C. F. King, T. K. Bauska, E. J. Brook, M. Kalk, C. Nehrbass-Ahles, E. W. Wolff, I. Strawson, R. H. Rhodes, M. B. Osman, Reconciling ice core CO_2 and land-use change following New World-Old World contact. *Nat. Commun.* **15**, 1735 (2024).

107. D. A. Stolper, W. W. Fischer, M. L. Bender, Effects of temperature and carbon source on the isotopic fractionations associated with O_2 respiration for $^{17}O/^{16}O$ and $^{18}O/^{16}O$ ratios in *E. coli*. *Geochim. Cosmochim. Acta* **240**, 152–172 (2018).

108. J. E. Heidenreich III, M. H. Thiemens, A non-mass-dependent isotope effect in the production of ozone from molecular oxygen. *J. Chem. Phys.* **78**, 892–895 (1983).

109. C. Janssen, J. Guenther, K. Mauersberger, D. Krankowsky, Kinetic origin of the ozone isotope effect: A critical analysis of enrichments and rate coefficients. *Phys. Chem. Chem. Phys.* **3**, 4718–4721 (2001).

110. S. M. Anderson, D. Hülsebusch, K. Mauersberger, Surprising rate coefficients for four isotopic variants of $O+O_2+M$. *J. Chem. Phys.* **107**, 5385–5392 (1997).

111. A. Schanz, K. Hocke, N. Kämpfer, Daily ozone cycle in the stratosphere: Global, regional and seasonal behaviour modelled with the Whole Atmosphere Community Climate Model. *Atmos. Chem. Phys.* **14**, 7645–7663 (2014).

112. G. Hancock, P. L. Tyley, The near-UV photolysis of ozone: Quantum yields of $O(^1D)$ between 305 and 329 nm at temperatures from 227–298 K, and the room temperature quantum yield of $O(^3P_2)$ between 303 and 310 nm, measured by resonance enhanced multiphoton ionisation. *Phys. Chem. Chem. Phys.* **3**, 4984–4990 (2001).

113. Y. Matsumi, F. J. Comes, G. Hancock, A. Hofzumahaus, A. J. Hynes, M. Kawasaki, A. R. Ravishankara, Quantum yields for production of $O(^1D)$ in the ultraviolet photolysis of ozone: Recommendation based on evaluation of laboratory data. *J. Geophys. Res.* **107**, 4024 (2002).

114. S. Madronich, S. Flocke, "The role of solar radiation in atmospheric chemistry," in *Environmental Photochemistry* (Springer, 1999), pp. 1–26.

115. Q. Fu, R. H. White, M. Wang, B. Alexander, S. Solomon, A. Gettelman, D. S. Battisti, P. Lin, The Brewer Dobson Circulation during the Last Glacial Maximum. *Geophys. Res. Lett.* **47**, e2019GL086271 (2020).

116. C. Risi, A. Landais, R. Winkler, F. Vimeux, Can we determine what controls the spatio-temporal distribution of d-excess and ^{17}O-excess in precipitation using the LMDZ general circulation model? *Clim. Past* **9**, 2173–2193 (2013).

117. M. Jung, C. Schwalm, M. Migliavacca, S. Walther, G. Camps-Valls, S. Koirala, P. Anthoni, S. Besnard, P. Bodesheim, N. Carvalhais, F. Chevallier, F. Gans, D. S. Goll, V. Haverd, P. Köhler, K. Ichii, A. K. Jain, J. Liu, D. Lombardozzi, J. E. M. S. Nabel, J. A. Nelson, M. O'Sullivan, M. Pallandt, D. Papale, W. Peters, J. Pongratz, C. Rödenbeck, S. Sitch, G. Tramontana, A. Walker, U. Weber, M. Reichstein, Scaling carbon fluxes from eddy covariance sites to globe: Synthesis and evaluation of the FLUXCOM approach. *Biogeosciences* **17**, 1343–1365 (2020).

118. IAEA/WMO, Global Network of Isotopes in Precipitation, The GNIP Database (2023); https://nucleus.iaea.org/wiser.

119. B. Luz, E. Barkan, Net and gross oxygen production from O_2/Ar, $^{17}O/^{16}O$ and $^{18}O/^{16}O$ ratios. *Aquat. Microb. Ecol.* **56**, 133–145 (2009).

120. D. P. Nicholson, R. H. R. Stanley, E. Barkan, D. M. Karl, B. Luz, P. D. Quay, S. C. Doney, Evaluation triple oxygen isotope estimates of gross primary production at the Hawaii Ocean Time-series and Bermuda Atlantic Time-series Study sites. *J. Geophys. Res.* **117**, C05012 (2012).

121. S. Piao, S. Sitch, P. Ciais, P. Friedlingstein, P. Peylin, X. Wang, A. Ahlström, A. Anav, J. G. Canadell, N. Cong, C. Huntingford, M. Jung, S. Levis, P. E. Levy, J. Li, X. Lin, M. R. Lomas, M. Liu, Y. Luo, Y. Ma, R. B. Myneni, B. Poulter, Z. Sun, T. Wang, N. Viovy, S. Zaehle, N. Zeng, Evaluation of terrestrial carbon cycle models for their response to climate variability and to CO_2 trends. *Glob. Chang. Biol.* **19**, 2117–2132 (2013).

122. G. D. Farquhar, S. von Caemmerer, J. A. Berry, A biochemical model of photosynthetic CO_2 assimilation in leaves of C_3 species. *Planta* **149**, 78–90 (1980).

123. N. Vuichard, P. Messina, S. Luyssaert, B. Guenet, S. Zaehle, J. Ghattas, V. Bastrikov, P. Peylin, Accounting for carbon and nitrogen interactions in the global terrestrial ecosystem

model ORCHIDEE (trunk version, rev 4999): Multi-scale evaluation of gross primary production. *Geosci. Model Dev.* **12**, 4751–4779 (2019).
